# A genetically tractable branch of environmental *Pedobacter* from the phylum Bacteroidota represents a hotspot for natural product discovery

**DOI:** 10.1038/s41598-025-03955-z

**Published:** 2025-06-20

**Authors:** Yang Liu, Luis Linares-Otoya, Christian Kersten, Michael Marner, Sanja Mihajlovic, Mohamed H. Abdeldayem, Sandra Semmler, Molly C. Bletz, Miguel Vences, Marius Spohn, Celine M. Zumkeller, Till F. Schäberle

**Affiliations:** 1 Institute for Insect Biotechnology with Focus on Natural Product Research, Justus- Liebig-University Giessen, Ohlebergsweg 12, 35392 Giessen, Germany; 2 Natural Product Department, Fraunhofer-Institute for Molecular Biology and Applied Ecology (IME-BR), Ohlebergsweg 12, 35392 Giessen, Germany; 3 Institute of Pharmaceutical and Biomedical Sciences, Johannes Gutenberg University Mainz, 55128 Mainz, Germany; 4 Department of Environmental Conservation, University of Massachusetts Amherst, Amherst, MA USA; 5 Zoological Institute, Technische Universität Braunschweig, Mendelssohnstr. 4, 38106 Braunschweig, Germany; 6 German Center for Infection Research (DZIF), Partner Site Giessen-Marburg- Langen, Ohlebergsweg 12, 35392 Giessen, Germany; 7 Present Address: Department of Molecular Biology, Princeton University, Princeton, USA

**Keywords:** Bacteroidota, Lipopeptides, Natural products, NRPS, *Pedobacter*, Biosynthetic gene clusters (BGCs), Dehydrovaline, Microbiology, Biological techniques, Bioinformatics, Genetic engineering, Genetic techniques, Genomic analysis, Isolation, separation and purification, Mass spectrometry, Metabolomics, Microbiology techniques, Sequencing

## Abstract

Emerging global challenges, such as antimicrobial resistance, have shifted the focus of natural product discovery from well-characterized microbial producers to underexplored taxonomic groups. Here, we computationally and experimentally characterize the biosynthetic potential of the genus *Pedobacter*, a Bacteroidota taxon known for antibiotic production that harbors numerous uncharacterized secondary metabolite (SM)-encoding biosynthetic gene clusters (BGCs). Through phylogenomic analysis of the genus *Pedobacter*, we identify a distinct clade enriched in lipopeptide-associated BGCs, most of which lack known chemical products. By developing de novo genetic tools and integrating metabolomics, we linked specific secondary metabolites to their corresponding BGCs. Using synthetic and analytical chemistry as proof of concept, we isolated and structurally characterized twelve linear lipopeptides (cryopeptins A–N), containing rare dehydrovalines from *Pedobacter cryoconitis* PAMC 27485. We demonstrate that all cryopeptins, despite their structural heterogeneity, are biosynthesized by a single multi-domain non-ribosomal peptide synthetase (NRPS) gene cluster. Mechanistically, we propose that this BGC drives chemical diversity through combinatorial fatty acid incorporation and iterative amino acid assembly, resulting in variable peptide chain lengths. This work highlights the biosynthetic versatility of *Pedobacter* and outlines methods for genetic manipulation in this genus to systematically access its cryptic natural product repertoire.

**Supplementary Information:**

The online version contains supplementary material available at 10.1038/s41598-025-03955-z.

## Introduction

Secondary metabolites (SMs) are small molecules that often exhibit ecologically or pharmacologically relevant bioactivities^1,2^. These compounds have evolved in bacteria to possess complex structural features in order to achieve potent and specific molecular interactions with their targets^3–5^. Given the structural complexity of SMs, these molecules are hard to mimic by synthetic chemistry and, therefore, serve as a primary source for the discovery of novel lead structures^4,6^.

Traditionally, actinobacteria, bacilli, and specific proteobacteria were major bacterial SM contributors. However, declining discovery rates prompt exploration of yet underexplored (bacterial) sources. Increasing capabilities to read, rate, and modify bacterial genomes are the keystone for a renaissance of SM discovery. Furthermore, the fact that most of the putative SM-encoding biosynthetic gene clusters (BGCs) have not yet been assigned to any chemical product indicates a high biosynthetic potential within non-traditional SM producer taxa^7–10^. Among these recently recognized taxa are the Bacteroidota, in which the number of compounds isolated and characterized represent only a minor proportion of the predicted potential^11^. Moreover, its encoded chemical diversity is predicted to differ from traditional natural products (NPs) -producing taxa^5,6^.

Our previous work analyzed representative genomes from the phylum Bacteroidota concerning their overall biosynthetic potential and identified the genus *Pedobacter* as a BGC hotspot^11^. Also, in 2021, Figueiredo et al. highlighted the *Pedobacter* to be the genus with the highest BGC number within the family *Sphingobacteriaceae*^12^. A recent pangenome study undermines this impression, suggesting a unique but uneven distribution of BGCs within the 41 *Pedobacter* genomes analyzed. *Pedobacter* species are widely distributed in many habitats, such as terrestrial^13^ and marine environments^14^or associated with higher organisms^15^. Although *Pedobacter* strains have been studied for their potential to produce industrially relevant enzymes^16,17^ and their relevance as antimicrobial resistance gene reservoirs^18,19^their potential for NPs discovery has been less explored. Members from this genus are known to produce antibiotics with potent activity against multidrug-resistant pathogens such as the cyclic lipodepsipeptides Pedopeptins and Isopedopeptins (from now on referred to as (iso)pedopeptins (molecules) or pedopeptin-like (BGCs)). (Iso)pedopeptins are the sole bioactive specialized metabolite class identified from *Pedobacter* to this date^20–22^.

Here, 143 *Pedobacter* genomes (including 21 project strains) were analyzed by genome mining, resulting in the identification of a *Pedobacter* branch enriched in yet uncharacterized non-ribosomal peptide synthetase (NRPS) gene clusters. To complement the traditional cultivation approaches, we developed a genetic toolkit for *Pedobacter* that enabled linking the natural products to their corresponding BGC in combination with metabolomics. This combined genetic-metabolomic approach led to the isolation of 12 lipopeptides that were fully characterized using nuclear magnetic resonance (NMR) experiments, Marfey´s analysis, and total synthesis.

## Results and discussion

### A Pedobacter branch is enriched in unique multimodular NRPS gene clusters

**Fig. 1:**
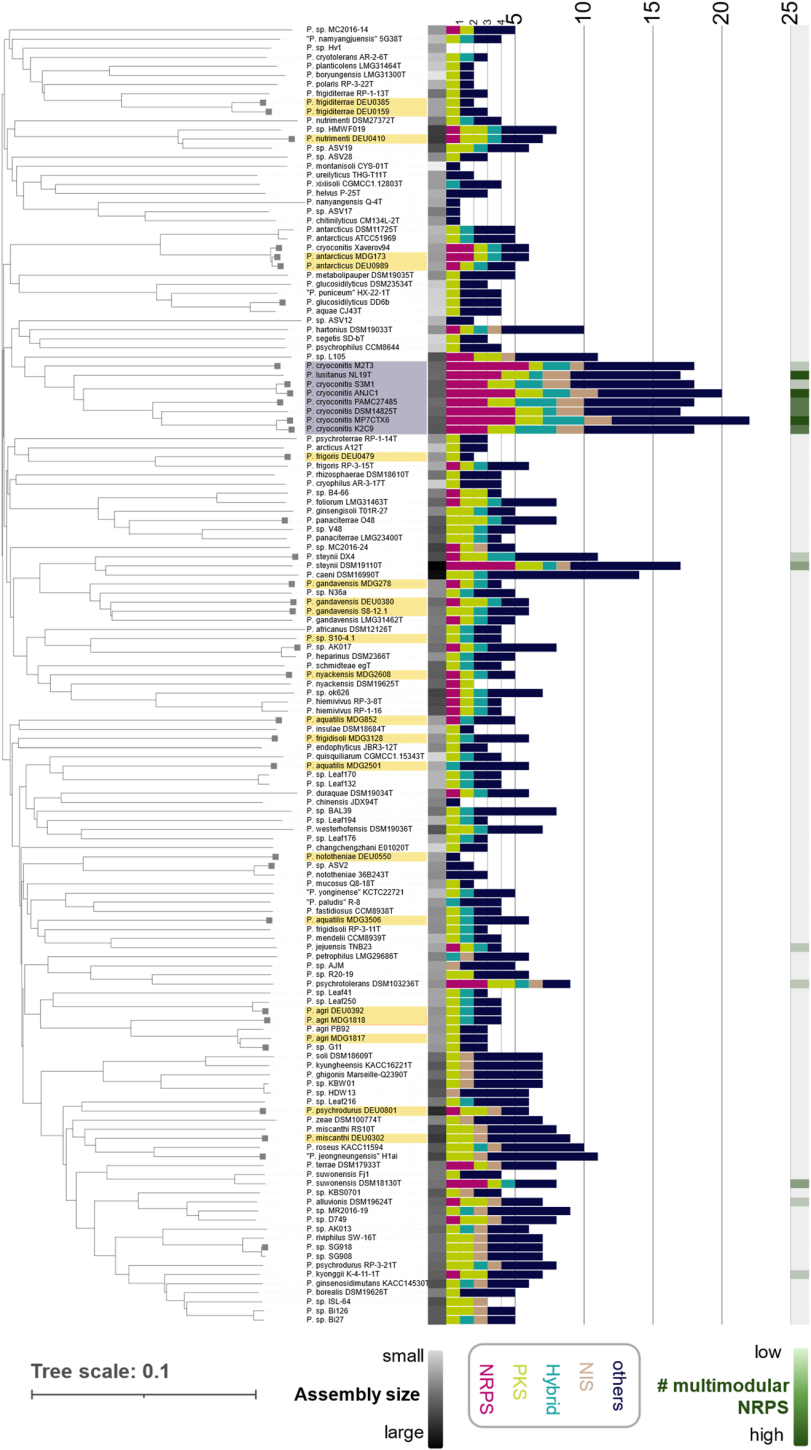
Bioinformatics analysis of 143 *Pedobacter* genomes indicates a BGC hotspot of the *P. cryoconitis* branch. A consensus tree was calculated using the Type Strain Genome Server^25^ based on the Genome BLAST Distance Phylogeny approach. Colors indicate project strains from amphibian specimens (yellow) and the *P. cryoconitis* branch (grey). The genome size (black heatmap), the number and types of biosynthetic gene clusters (bar charts), and the presence of multimodular NRPS gene clusters (green heatmap) are illustrated for each strain. Values were determined by antibiotics & Secondary Metabolite Analysis Shell (antiSMASH) v6.1.1^27^. The tree was annotated using the interactive tree of life (iTOL v6^28^) tool. The species names are based on NCBI entries. Species affiliations of some strains remained inconclusive (marked with a square) if compared with different tools (OrthoANI^24^, TYGS^25^ and GTDB^26^). The supplementary table also lists species affiliations based on TYGS for these strains.

To comprehensively rate the biosynthetic potential of the genus *Pedobacter*, we extracted 149 genomes from the National Center for Biotechnology Information (NCBI) reference genome database. This dataset was complemented with 21 project strain genomes^23^ from amphibian specimens sampled either in Germany (ISO country-code DEU) or in Madagascar (ISO country-code MDG) (details under BioProject accession number PRJNA1019955). After data curation (Material and Methods), we constructed a whole-genome sequence-based phylogenetic tree, including 143 *Pedobacter* genome sequences (Fig. 1). Some species affiliations published by NCBI were inconclusive when evaluated using different genome-based classification tools^24–26^ (Fig. 1, grey squares). Supplementary Table 1 contains suggested species affiliations for these strains based on TYGS^25^. We found the 21 *Pedobacter* strains from amphibian specimens (yellow shade) broadly distributed across the genus *Pedobacter*.

Next, we assessed and rated the biosynthetic potential of the strains by BGC annotation analysis using antibiotics & Secondary Metabolite Analysis Shell (antiSMASH) 6.1^27^ (Supplementary Table 1). In summary, a *Pedobacter* genome featured 5.7 BGCs on average. The overall biosynthetic diversity is similar to the numbers reported by Covas et al. (This study vs. Covas et al.); While cluster types like NRPS (11% vs. 9%), Ribosomally synthesized and post-translationally modified peptides (RIPPs) (27% vs. 27%), Terpenes (22% vs. 22%) and Others (8% vs. 7%) are very similar, notable differences are observed in Polyketide synthases (PKS) (19% vs. 12%) and NRPS-PKS hybrids (12% vs. 17%). This discrepancy can be explained by the different size and composition of the genomic datasets. As indicated by Covas et al.^29^, there is no overall linear positive correlation between genome assembly size and the number of BGCs (*R*^2^ = 0.29; Fig. S1-3). This differentiates *Pedobacter* from other bacterial genera, such as, e.g., *Chitinophagia*^11^, *Nocardia*^30^, or *Amycolatopsis*^31^, showing a positive correlation between the BGC number of each strain and the respective genome size. The accumulation of clusters in only a few genomes already hints towards an uneven distribution of BGCs within the genus. To identify strains with a high BGC load, the determined total BGC number was assigned to the strains and set to the taxonomic context of the genus based on the calculated phylogenetic tree (Fig. 1). The eight strains exhibiting the highest BGC load (≥ 17 BGCs) cluster together in the phylogenetic tree (Fig. 1, grey shade), including the two type strains, *P. cryoconitis* DSM 14825^32^ and *P. lusitanus* NL19^T^^33^. This is in accordance with the analysis performed by Covas et al., which also identified *P. cryoconitis* and *P. lusitanus* as hotspots of biosynthetic potential. Our analysis now extends this hotspot - the “*P. cryoconitis* branch” by six additional strains. As shown in Fig. 1, all *P. cryoconitis* strains - except the type strains DSM 14825^T^ - exhibit taxonomic discrepancies. According to TYGS^25^ analysis, these strains should be reassigned to *Pedobacter sp.* However, for consistency, we retained the NCBI naming throughout the manuscript. These eight strains show an average genome size of 6.13 ± 0.14 Mbps, encoding 18.25 ± 1.6 BGCs. All other *Pedobacter* strains (*n* = 135) instead have an average genome size of 5.42 Mbps and a comparatively low BGC load of 5.1 ± 2.8 BGCs. Despite their close relationship, their BGC composition is still rather heterogeneous (cosine similarity score ranging from 0.5 to 1, Fig. S4B). Only *P. cryoconitis* K2C9 and *P. cryoconitis* MP7CTX6 share their complete BGC equipment. The *P. cryoconitis* branch accounts for 18% of all BGCs and a remarkably enriched amount of NRPS BGCs. Together, they carry 34 NRPS gene clusters (on average 4.25 ± 1.1 per strain), accounting for 43% of all NRPS gene clusters detected within the dataset.

The biosynthetic potential of *Pedobacter* in the production of terpenes and NRPS-independent siderophore (NIS) SMs was previously reported^33^. In this study, we focused on NRPS-derived SMs instead. NRPS biosynthetic machineries provide a tremendous structural complexity of peptides exhibiting diverse biological functions and a wide range of physio-chemical properties^34^.

**Fig. 2:**
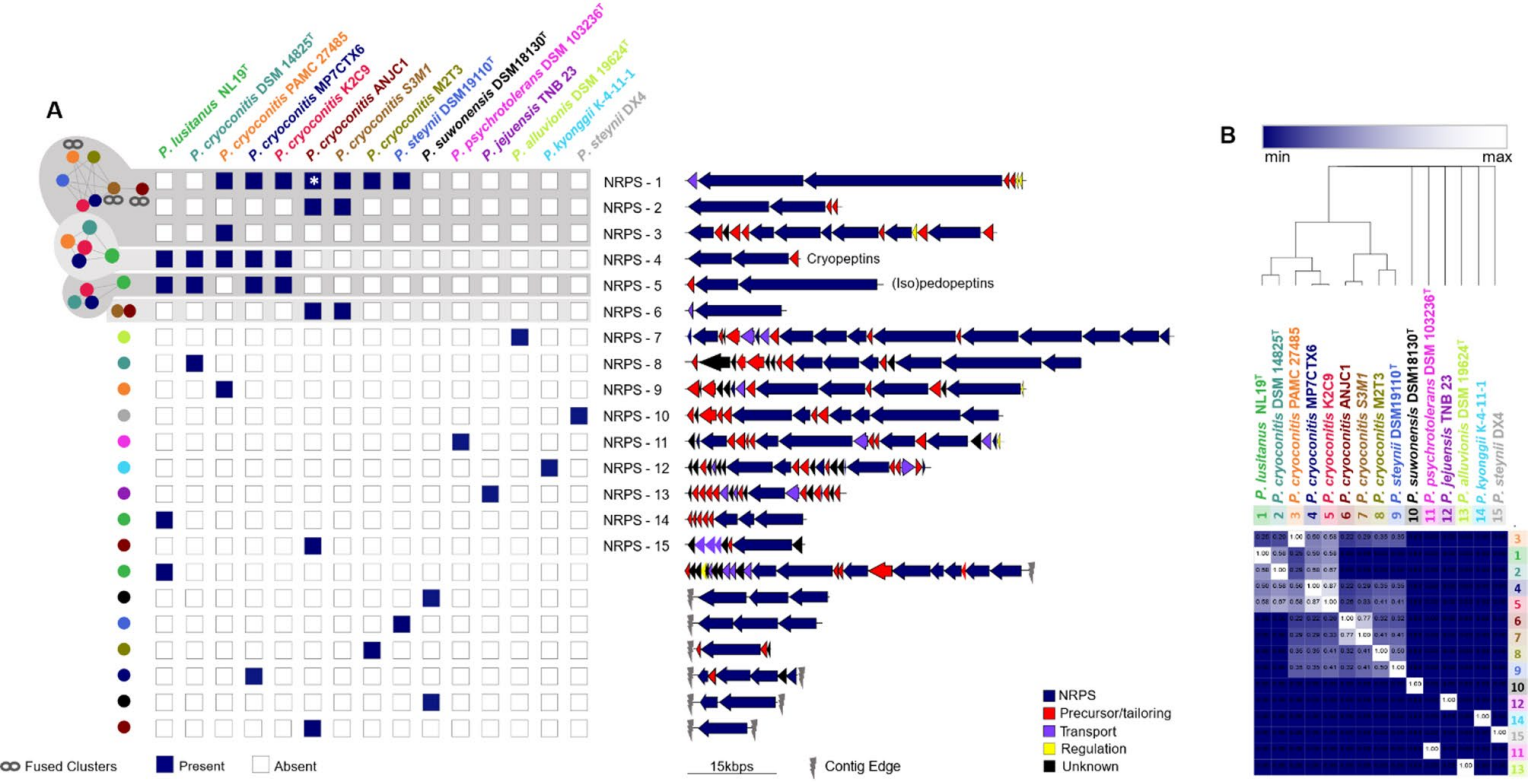
BGC Similarity of multimodular NRPS from the genus *Pedobacter* reveals a high diversity. A BiG-SCAPE^35^ analysis of 34 multimodular NRPS gene clusters from 15 strains was performed to analyze distribution and diversity (Strain-specific colored nodes and networks, left side). The presence of NRPS clusters was manually curated (blue and white boxes), thereby revealing the presence of fused clusters (chain sign, NRPS 1, 2, 3) and broken clusters (white star, NRPS-1). The architecture of multimodular NRPS clusters is highlighted on the left side (colored arrows), numbered from 1 to 15. Incomplete NRPS clusters at contig edges (grey flash) are not numbered. B Heat map illustrating the cosine similarity of the multimodular NRPS composition from blue (minimum similarity) to white (maximum similarity). The strains are clustered based on similarity, as highlighted by the tree^37^. Strains 10-15 all encode unique multimodular NRPS clusters (similarity score: 0). Strains belonging to the *P. cryoconitis* branch and *P. steynii* DSM 19110^T^ form a separate branch that share some of the multimodular NRPS between each other.

Our in-depth analysis revealed the annotation of NRPS gene clusters within the genomes of 41 strains. Of these, 26 strains exclusively contained monomodular NRPS gene clusters while a rather exclusive amount of 15 strains carried 34 multimodular NRPS gene clusters. Most of these multimodular NRPS gene clusters are again encoded within the genomes of the *P. cryoconitis* branch (Fig. 1, green heat map). Another seven strains carrying multimodular NRPS gene clusters are distributed over the genus. To determine the diversity of the 34 detected multimodular NRPS gene clusters, we used BiG-SCAPE^35^ (Biosynthetic Gene Similarity Clustering and Prospecting Engine) to analyze their sequential and compositional similarity and to assign them to gene cluster families (GCFs). At a cutoff of 0.6, 22 GCFs were generated. Three networks included GCFs from more than two strains, covering 16 BGCs in total (Fig. 2, NRPS-1, -4, and − 5). Corason^35^ alignment (Fig. S1-6) of these GCFs showed that the GCF defining NRPS-1 actually contained three nodes with fused NRPSs (Fig. 2A-chain symbol), while NRPS-1 is only partially covered in strain *P. cryoconitis* ANJC1 (Fig. 2A-star). NRPS-1 GCF also comprises a duplet harbored by strains *P. cryoconitis* S3M1 and ANJC1 and another singleton in *P. cryoconitis* PAMC 27485. NRPS-1 is most conserved and found in 7 of the 15 analyzed strains. Notably, 6 out of 8 strains of the *P. cryoconitis* branch encode this BGC, including *P. steynii* DSM 19110^T^, which does not belong to the same taxonomic group. NRPS-4 is a smaller yet unassigned BGC harbored by a third of the investigated strains, while NRPS-5 is present in four strains. Both, NRPS-4 and − 5 are only found in members of the *P. cryoconitis* branch. Two GCFs were duplicates (NRPS-2 and − 6), all encoded by *P. cryoconitis* ANJC1 and S3M1, respectively. The rest of the detected BGCs remained as singletons (*n* = 17).

To assign the detected clusters to known BGCs, BigSCAPE^35^ was used to include and calculate the similarity of all reference BGCs available from the Minimum Information about a Biosynthetic Gene cluster (MIBiG^36^) v 2.1 repository. Intriguingly, no deposited reference cluster was correlated to our data, suggesting a compositional uniqueness of the analyzed *Pedobacter* NRPS BGCs. Manual inclusion of the known Pedopeptin BGC, however, confirmed its presence in the described producer *P. lusitanus* NL19^T^, and similar pedopeptin-like clusters in *P. cryoconitis* DSM 14825^T^, *P. cryoconitis* K2C9 and *P. cryoconitis* MP7CTX6 (Fig. 2, NRPS-5, Fig. S1-6 C).

Finally, we generated a cosine similarity score heatmap to visualize the similarity of the multimodular NRPS content of the strains^37^ (Fig. 2B). The strains outside of the *P. cryoconitis* branch all encode unique multimodular NRPSs, resulting in their similarity score of zero for all strains. The strains of the *P. cryoconitis* branch, highly enriched in multimodular NRPSs, form a distinct group within the genus. Within this branch, two separate groups become obvious: One includes the five strains *P. cryoconitis* PAMC 27485, *P. cryoconitis* DSM 14825^T^, *P. cryoconitis* K2C9, *P. cryoconitis* MP7CTX6 and *P. lusitanus* NL19^T^ (similarity score range of 0.3–0.9). The strains PAMC 27485 and DSM 14825^T^ share the lowest similarity because only NRPS-4 is shared between them. The group is formed by *P. cryoconitis* ANJC1 and *P. cryoconitis* S3M1, showing a high similarity score of 0.8. The wide range of similarities highlights the BGC heterogeneity within the *P. cryoconitis* branch.

### Targeted deletion of NRPS-4 confirms its role in producing the target lipopeptide family

Our in-silico analysis highlighted a *Pedobacter* branch possessing a great diversity of unique BGCs that differ from those in the MIBiG^36^ database (Fig. S4A). This prompted a yet uncharted chemical space of SMs potentially produced by this *Pedobacter* branch. To link BGCs to their corresponding metabolites, we sought to disrupt the putative BGC and perform comparative metabolomics to identify the associated metabolites. To this end, we initially evaluated the metabolome of representative *Pedobacter* under diverse culture conditions, identified candidate NRPS-like metabolites via tandem mass spectrometry (MS/MS) analysis, and subsequently used this information to computationally identify expressed NRPS gene clustersthat could be experimentally validated by genetic engineering.

We selected the strains *P. cryoconitis* PAMC 27485 and *P. cryoconitis* DSM 14825^T^ as representative strains from this branch. Although closely related, they show a distinct BGC composition (overall BGC similarity score of 0.75). We cultivated both strains in different media (MYE, TSB, R2A, LB, NB, and POM) and applied different extraction methods (ethyl acetate or butanol liquid-liquid extraction (LLE) and C18-based SPE) (Fig. S5) to maximize the SM production and its recovery. Obtained crude extracts were subjected to ultrahigh performance liquid chromatography-electrospray ionization tandem mass spectrometry (UPLC-HR-ESI-MS/MS) measurement and the acquired data was visualized by molecular networking (Fig. S5). The curated network comprised 1974 parent ions, excluding culture media-derived ions. *P. cryoconitis* DSM 14825^T^ (30%, *n* = 589) or *P. cryoconitis* PAMC 27485 (36%, *n* = 702). To determine whether known metabolites and or its derivatives were present in the *P. cryoconitis* extracts, we applied the Global Natural Products Social Molecular Networking (GNPS) automated dereplication tool^38^. This revealed no database match. However, a manual inspection for known metabolites from *Pedobacter* with a particular focus on NRPS-like molecules enabled the dereplication of the Lipopolysaccharide-binding antibiotics (iso)pedopeptin in extracts of strain *P. cryoconitis* DSM 14825^T^ when cultivated in R2A and using C18 or butanol extraction. The presence of (iso)pedopeptin was connected to the double-charged ions *m/z* 550.3, 559.3 and 566.3 [M + 2 H]^2+^. (Iso)pedopeptins were not detected in extracts of *P. cryoconitis* PAMC 27485 (Fig. S9). These results align with our BGC analysis that identified a pedopeptin-like BGC in the *P. cryoconitis* DSM 14825^T^ genome, while not in PAMC 27485^39^ (Fig. 2, NRPS-5, Fig. S1-6 C).

Additionally, we identified a cluster of peptide-like compounds with m/z values ranging between 670 and 754, produced by both *P. cryoconitis* strains in 6 tested media (Fig. S5). Further analysis of exact m/z 699.420 and m/z 713.434 [M + H] + and MS/MS fragmentation pattern revealed two known peptides isolated from *Pedobacter* strains in a previous study^40^. MS/MS analysis suggested the new derivatives were structural variations in their fatty acids side chains (Fig. S5, cloud a-j). Despite the structural diversity observed here and previously, the common structural features suggest that the biosynthesis of these peptides could be encoded in a single NRPS BGC. Motivated by this finding, we targeted this peptide family for further structural and biosynthetic characterization.

**Fig. 3:**
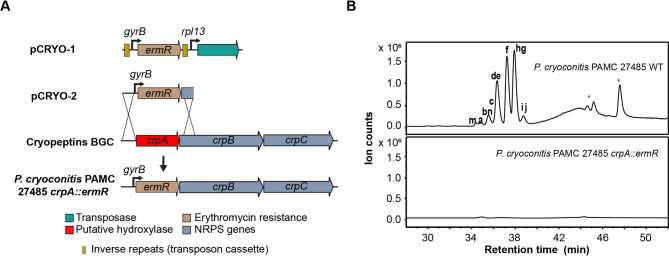
Genetic manipulation strategy (A) for identifying the BGC-associated SM by comparative metabolomics (**B**). (**A**) Two strategies were applied to exchange the target gene *crpA* (red arrow) with an erythromycin resistance gene (brown); pCRYO1 encodes a transposase gene (light blue) for exchange by transposition, while pCRYO2 is a suicide plasmid resulting in targeted deletion by homologous recombination. (**B**) Extracted ion chromatogram of *P. cryoconitis* WT and *P. cryoconitis crpA::ermR* extracts (full diagram in Fig. S10). Cryopeptin peaks are labelled from a-n, stars indicate compounds with similar masses that are not structurally related to the cryopeptins.

Based on the analysis of the multimodular NRPS composition (Fig. 2A), we investigated NRPS-4, the only cluster present in both strains, in more detail (Fig. S1-4). The module number (*n* = 5) and the adenylation domain specificity (X-val-X-val-X, only mentioned if the same specificity in all strains) matched the structural features of the target peptides. Besides *P. cryoconitis* PAMC 27485 and *P. cryoconitis* DSM 14825^T^, NRPS-4 is also detected in three more strains of the *P. cryoconitis* branch: *P. lusitanus* NL19^T^, *P. cryoconitis* K2C9 and *P. cryoconitis* MP7CTX6. The five modules are encoded within two NRPS genes (*crpB* and *crpC*) (Fig. 3A and S1-4) expectedly involved in peptide formation, as well as a C-starter domain that is predictively involved in the acylation of the first incorporated amino acid. A third gene (*crpA*) was annotated as a metallo beta-lactamase gene and is conserved throughout all strains carrying this BGC (Fig. S1-4).

To initially test a genetic manipulation strategy, we first screened the strains *P. cryoconitis* PAMC 27485 and *P. cryoconitis* DSM 14825^T^ for sensitivity against different antibiotics and found that erythromycin (100 µg/mL) and chloramphenicol (150 µg/mL) inhibited the growth of both wild-type strains indicating that their resistance genes are suitable selection markers. Since no replicable plasmid has been described for *Pedobacter*, we initially optimized the gene transfer technique using a transposon system that does not rely on intrinsic strain DNA recombination levels to integrate the resistance marker in the chromosome. We built a random transposon insertion plasmid (pCRYO1 (Fig. S2)) based on a HIMAR transposon pSAMbt system, initially designed for *Bacteroides thetaiotaomicron* in which we introduced the constitutive *P. cryoconitis* promoters of *gyrB* and *rpl13* and used erythromycin as a resistance marker^41^ (Fig. 3A). We obtained erythromycin-resistant clones by conjugation and electroporation and confirmed the presence of the *ermR* gene by colony PCR (Material and Methods).

Having optimized a gene transfer method, we aimed to perform a targeted deletion of genes in the NRPS-4 BGC via allelic exchange through homologous recombination. For this, we constructed a suicide plasmid pCRYO2 (Fig. S3-1) by exchanging the transposon cassette and transposase of pCRYO1 with an *ermR* gene. To inactivate the *P. cryoconitis* PAMC 27485 BGC by gene deletion, 2 kbp regions flanking *crpA* were placed upstream and downstream of *ermR* in pCRYO2. After transfer of this plasmid in the wild type by conjugation, a double crossover event resulting in mutant strain PAMC 27485* crpA::ermR* was seen in 5% of screened conjugants, while the remaining recombinants had single crossover insertions (Fig. S3-2).

To determine whether this *crpA* deletion indeed stopped the production of the assigned putative encoded peptides, PAMC 27485 *crpA::ermR* and WT strains were cultured, extracted, and analyzed (Fig. 3B and Fig. S6). The extracts were subjected to Ultra-performance liquid chromatography High-Resolution Mass Spectrometry (UPLC-HR-ESI-MS), and the acquired MS/MS data was used to build a molecular network based on fragmentation patterns of the metabolites present in extracts using the GNPS molecular networking tool. Our results clearly showed that the production of the targeted peptide metabolite cluster (weight range of 670–754 Da) was abolished in PAMC 27485 *crpA::ermR*, while present in the WT strain (Fig. 3B and Fig. S6). Additionally, three other clusters (Fig. S6, orange cluster 1,2,4) appeared in the WT extracts but not the ∆*crpA* mutant. Detailed MS/MS analysis of cluster 4 (m/z 483.806, 490.811, 497.821 [M + 2 H]^2+^) revealed heptapeptide derivatives structurally related to the previously identified pentapeptide family. These were also predicted by MS/MS from *Pedobacter* strains investigated by Bjerketorp et al.^40^(Fig. S5, Fig. S6).

### The cryopeptins: a structurally diverse lipopeptide family

**Fig. 4:**
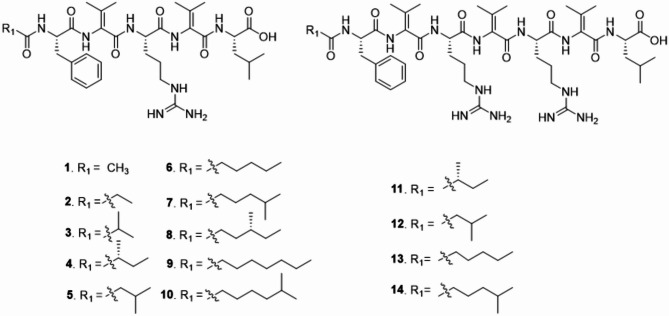
Chemical structure of the cryopeptins 1–14 (**A-N**). The cryopeptins are linear lipopeptides consisting of either a pentapeptide backbone (*L-*Phe-Dhv-*L-*Arg-Dhv-*L-*Leu; cryopeptins A-J) or a heptapeptide backbone (*L-*Phe-Dhv-*L-*Arg-Dhv-*L-*Arg-Dhv-*L-*Leu; cryopeptins K-N). They differ concerning their *N*-terminal lipid tail (R_1_, R_2_).

Next, to chemically characterize the NRPS-4 assigned lipopeptidic chemical products, we cultivated *P. cryoconitis* PAMC 27485 at a larger scale. We isolated 12 new structurally related lipopeptides, here named cryopeptins (Fig. 4). The structures of those 12 compounds were elucidated by NMR analysis, and the stereochemistry was determined by comparing^1^H NMR spectra of natural and chemically synthesized compounds as well as Marfey’s analysis.

The major secondary metabolite was the linear peptide cryopeptin D (**4**), with a molecular formula of C_36_H_56_N_8_O_7_ indicated by UPLC-HR-ESI-MS at *m/z* 713.4343 [M + H]^+^ (calcul. C_36_H_57_N_8_O_7_, *m/z* 713.4345 [M + H]^+^). The peptidic nature of cryopeptin D (**4**) was evident from three *α*-amino acid protons (δ_H_ 4.50, δ_H_ 4.20, δ_H_ 3.93) in the^1^H NMR spectrum and the correlation with the amide-carbonyl groups of δ_C_ 170.65, δ_C_ 171.10, δ_C_ 175.80. The two additional amide-carbonyl groups resonated at δ_C_ 165.09, δ_C_ 164.44, together with 5 amide protons resonating at δ_H_ 9.19, δ_H_ 8.96, δ_H_ 8.15, δ_H_ 7.85, δ_H_ 7.27 indicated two more amino acids without *α*-amino acid proton. Extensive elucidation of **(**homonuclear correlation spectroscopy) COSY, heteronuclear single quantum correlation (HSQC) and heteronuclear Multiple-Bond Correlation (HMBC) NMR datasets enabled to determine the amino acid residues as Phe, Leu, Arg, and two dehydro-Val. The remaining NMR signals established the starting unit of **4** as 2-methylbutanoyl (Table S5, Fig. S14). To determine the stereo-center of the 2-methyl group, we chemically synthesized *L-*Ile-Dhv-*L-*Arg-Dhv-*L-*Phe-2*S*-methylbutanoyl (see SI: Chemical Synthesis). Comparison of both, native (**4**) and synthetic (**4.4.** Fig. S36-38) NMR spectra revealed a 2*S* configuration of the natural product (Table S10). The absolute stereochemistry of Phe, Leu, Arg of **4** was determined by using advanced Marfey’s analysis. Retention time comparison with reference *D* and *L-* amino acids indicated *L-*Phe, *L-*Arg, and *L-*Leu, respectively (Fig. S31-S33). Cryopeptin D (**4**) shared the same core structure with a previously described linear lipopentapeptide^40^. Here, it was accordingly named cryopeptin E (**5**), possessing a 2-methylbutanoyl starter unit (Table S5, Fig. S15). In accordance with the molecular network (Fig. S5), seven additional molecules of the cryopeptins network cluster, together with the two reported compounds cryopeptins C (**3**) (Fig. S13) and E^40^ (**5**), were structurally characterized in the same way. These compounds were identified with molecular formula of C_33_H_50_N_8_O_7_ (**1**, cryopeptin A *m/z* 671.3884 [M + H]^+^, Table S4, Fig. S11), C_34_H_52_N_8_O_7_ (**2**, cryopeptin B *m/z* 685.4032 [M + H]^+^, Table S4, Fig. S12), C_37_H_58_N_8_O_7_ (**6**, cryopeptin F *m/z* 727.4506 [M + H]^+^, Table S6, Fig. S16), C_38_H_60_N_8_O_7_ (**7**, cryopeptin G *m/z* 741.4664 [M + H]^+^, Table S6, Fig. S17; **8**, cryopeptin H *m/z* 741.4658 [M + H]^+^, Table S6, Fig. S18), C_39_H_62_N_8_O_7_ (**9**, cryopeptin I *m/z* 755.4811 [M + H]^+^, Table S7, Fig. S19; **10**, cryopeptin J *m/z* 755.4814 [M + H]^+^, Table S7, Fig. S20). Cryopeptins A-J share the same amino acid skeleton, the only difference exists in the starting unit of the fatty acid, which is also indicated by the observed UPLC-HR-ESI-MS mass ions with 14 Da (-CH_2_-) differences from each other.

Additionally, four cryopeptins K-N (**11**–**14**) consisting of 7 amino acid residues were isolated. The molecular formula of cryopeptin K (**11**) was determined to be C_47_H_75_N_13_O_9_ (*m/z* 483.7987 [M + 2 H]^2+^). The substructure of 2-methylbutanoyl, Arg, Phe-Dhv, and Dhv-Arg-Dhv-Leu residues in cryopeptin K (**11**) was unambiguously elucidated by NMR spectra (Table S8, Fig. S21). In combination with the MS/MS fragmentation pattern, the order of each substructure was clarified as 2-methylbutanoyl-Dhv-Phe-Arg-Dhv-Arg-Dhv-Leu (Fig. S21-7). The absolute configuration of the 2-methyl group in the fatty acid chain was assumed as *S* configuration by comparing the NMR data with the synthetic *L-*Ile-Dhv-*L-*Arg-Dhv-*L-*Phe-2*S*-methylbutanoyl (Table S10). Cryopeptin L (**12**) shared the same molecular formula C_47_H_75_N_13_O_9_ (*m/z* 483.7988 [M + 2 H]^2+^) with **11.** Comparing the NMR spectra and the MS/MS fragmentation pattern (Fig. S22-7) with **11**, the only difference is the starting unit, i.e., 3-methylbutanoyl (Table S8, Fig. S22). Cryopeptins K and L shared the same molecular weight with a reported compound *m/z* 483.799 [M + 2 H]^2+^, which was before only indicated by the MS/MS pattern^40^.

The remaining two new peptides were elucidated as cryopeptins M (**13**) and N (**14**) with the molecular formula of C_48_H_77_N_13_O_9_ and C_49_H_79_N_13_O_9_ indicated by *m/z* 490.8065 [M + 2 H]^2+^ and *m/z* 497.8147 [M + 2 H]^2+^, respectively. The NMR data revealed the fatty acid chain of cryopeptin M (**13**, Table S9, Fig. S23) as a hexanoyl, and of cryopeptin N (**14**, Table S9, Fig. S24) as a 5-methylhexanoyl.

The cryopeptins are linear lipopeptides containing the non-proteinogenic amino acid dehydrovaline. This amino acid is a rare building block, and its occurrence is yet limited to linear peptides. For instance, dehydrovaline is found in cytotoxic yakuamides from the sponge *Ceratopsion* sp^42^. and in the myxobacterial antibiotic myxovalargin^43^. However, screening against bacterial and fungal indicator strains^44^ revealed no growth inhibitory effects of cryopeptin D (**4**) and E (**5**) (Minimal inhibitory concentration > 128 µg/ml, Table S11). In addition, **4** and **5** underwent screening against the human cysteine proteases cathepsin B and L, and rhodesain, the cysteine protease of *Trypanosoma brucei rhodesiense* according to the procedure described in^45^. However, no protease inhibitory activity was observed at the tested concentration (20 µM).

### Cryopeptin biosynthesis involves iterative use of NRPS modules and dehydrovaline formation

**Fig. 5:**
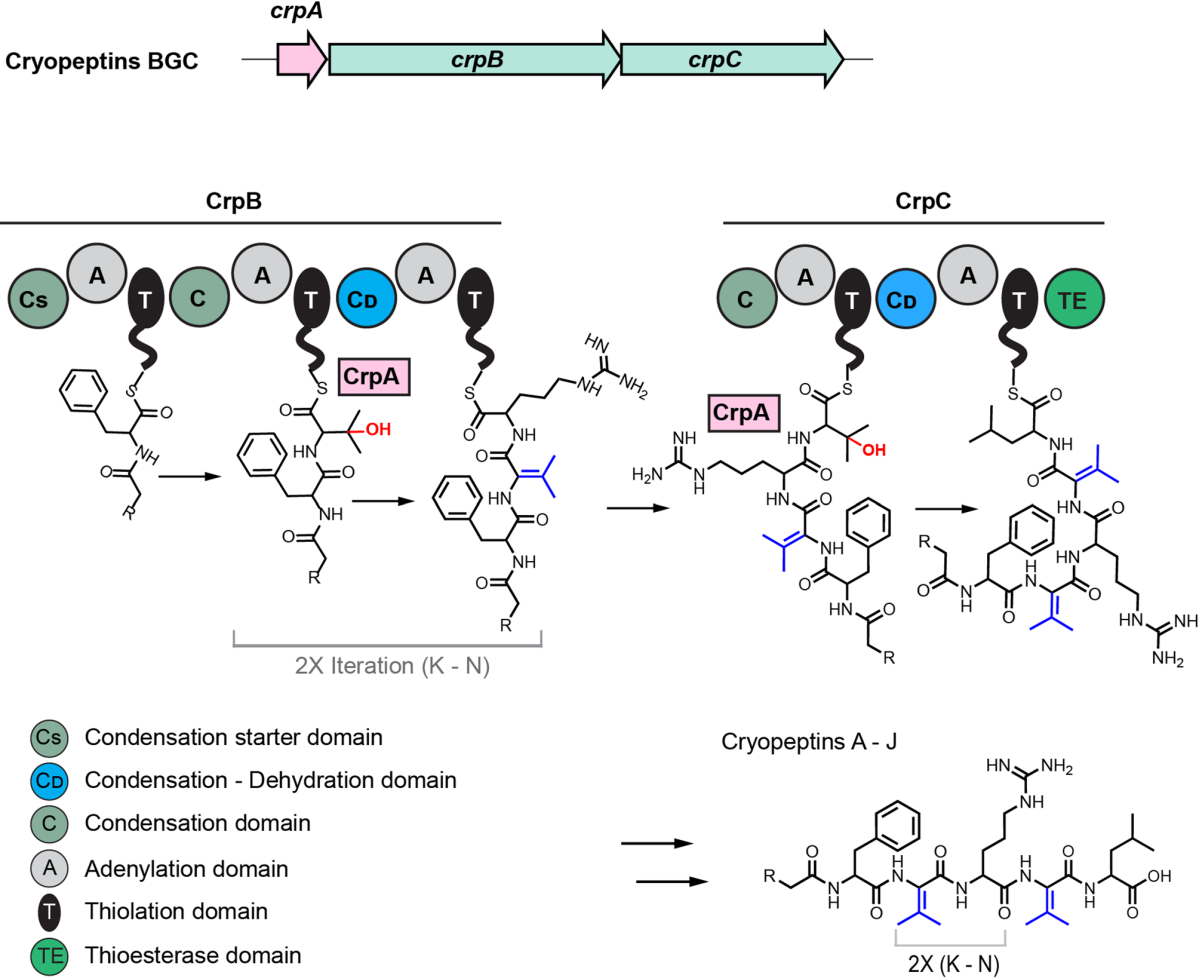
Proposed biosynthesis of the cryopeptins. The BGC consists of two NRPS genes (*crpB*, *crpC*). CrpB contains a condensation starter domain responsible for the attachment of the fatty acid side chain. We propose that CrpA hydroxylates valine as an intermediate step toward dehydrovaline formation. An iterative assembly mode of CrpB could explain the presence of heptapeptidic cryopeptins.

Based on the bioinformatic BGC analysis and determined cryopeptin structures, a cryopeptin biosynthetic pathway is proposed. The conserved features (Fig. S1-4) found in all five assigned cryopeptin BGCs include *crpA*, which codes for a putative metallo-beta lactamase, and the two NRPS genes *crpB* and *crpC* (Fig. 5). The predicted assembly line constituted by CrpB and CrpC matched the determined lipo-pentapeptidic structures of the cryopeptins in their multimodularity. *P. cryoconitis* DSM 14825^T^ and *P. cryoconitis* PAMC 27485 share the same A-domain substrate specificity (X-val-x-val-val), which is why we would expect the same amino acid composition. All other cryopeptin BGC encoding strains were not investigated for their cryopeptin production and structure. While *P. lusitanus* NL19^T^ shares its A-domain specificity with the DSM 14825^T^ and PAMC 27485 (as predicted by antiSMASH^27^), MP7CTX6 (val-val-x-val-tyr) and K2C9 (x-val-tyr-val-val) slightly differ from this prediction (Fig.S1-4). Whether these differences in A-domain specificity translate into variations in the actual amino acid composition of the produced compounds remains to be investigated. Furthermore, the stereochemistry of the Phe, Leu, and Arg residues was conclusively determined as the *L-*configuration using Marfey’s analysis, corroborating the in-silico analysis that does not predict any epimerization domains within the BGC (Fig. S1-4).

The role of CrpA, however, is less straightforward and deducible. CrpA belongs to the metallolactamase protein family, whose members have been identified in NRPS gene clustersmostly as hydroxylases. This is the case of leucine hydroxylase (MIBiG ID: BGC0000893^36^, CmlA, Coverage: 99% Identity: 38%from FR900359 (MIBiG ID: BGC0002125^36^, FrsH, Coverage: 99% Identity: 38%) BGC. However, no leucine is hydroxylated in the case of cryopeptins. This leads us to hypothesize that CrpA could hydroxylate valine as an intermediate step toward dehydrovaline formation. Supporting this hypothesis, we found that the condensation domains C3 and C5 belong to the subclass - “modified AA”, according to NaPDoS analysis^46^ (Fig. S1-5). Such condensation domains seem to be involved in amino acid dehydration processes, similar to the condensation reaction in the methoxyvinylglycine biosynthesis^47^. Therefore, these condensation domains are likely involved in dehydrovaline formation from hydroxyvaline. Another uncommon aspect of cryopeptin biosynthesis is the repeated Dhv-Arg motive in the heptapeptidic cryopeptins, resulting in an additional structural diversification of the cryopeptins. The biosynthetic mechanism of this repeated Dhv-Arg-attachment could not be directly deduced from the NRPS assembly line structure. However, we propose an iterative activity of module 2 and module 3 encoded in *crpB* (Fig. 5). For example, such iteratively used modules were recently exemplified in the solanimycin biosynthesis, where a dehydroalanine residue is iteratively loaded (five times) in the nascent peptide by the NRPS protein SolG^48^.

## Conclusion

The cryopeptins, despite lacking detectable biological activities in our screenings, were revealed through a combination of metabolic and genomic networking and the developed genetic toolkit. Besides detecting and linking the respective BGC to the corresponding metabolite(s), genetic tools, as established here, can find applications in various scenarios. For example, high-throughput utilization of transcriptional reporters could aid in comprehending the temporospatial BGC regulation of these organisms under diverse physicochemical conditions^49,50^. Alternatively, BGC expression could be directly increased by replacing the BGC regulatory regions with a strong constitutive promoter^51^; thus bypassing the intricate regulatory networks. Such an approach could also activate BGCs before silent under laboratory conditions to discover new metabolites^52^. Furthermore, the generation of knock-out mutants could aid functional, microbiological studies providing new insights into the genus *Pedobacter*.

The integrative metabologenomics approach has the potential to reveal further the novel chemistry encoded by this unexplored BGC-rich branch and related Bacteroidota taxa. This will contribute to understanding the ecological roles of these strains and the biosynthesized metabolites and enable biotechnological applications of natural products of interest.

## Material and methods

### Strains, media and culture conditions are described in the SI

#### Creation and curation of the *Pedobacter* assembly dataset

149 *Pedobacter* assembly genomes matching our selection criteria (NCBI: txid84567, filter: RefSeq annotation) were obtained from NCBI in gbff file format on 28.11.2022. The dataset was complemented by 21 *Pedobacter* assemblies retrieved from amphibian specimens (Biosample accession 37505411–37505431) (*n* = 171). To ensure data quality, genome assemblies were subject to quality control steps using CheckM2^53^, TYGS^25^OrthoANI^24^and Genome Taxonomy Data Base (GTDB-Tk)^26^. Firstly, we retained only assemblies meeting the minimum information about a metagenome -assembled genome (MIMAG) evaluation^54^ criteria for high-quality genomes, demonstrating 90–100% completeness and 0–5% contamination according to CheckM2 analysis (*n* = 168). Redundant genomes were identified and removed based on orthoANI scores greater than 99.9% and TYGS digital DNA-DNA hybridization (dDDH) values (d0, d4, d6 = 100%).For the remaining genomes, GTDB-Tk was used to confirm taxonomic affiliation. The final dataset consisted of 143 genome assemblies, all confirmed to belong to the genus *Pedobacter.* A whole-genome sequence-based phylogenetic tree was retrieved from TYGS and visualized using the interactive tree of life (iTOL v6^28^) tool. Metadata such as assembly size and N50 were monitored using Geneious (v11.1.5).

#### Detection and clustering of biosynthetic gene clusters (BGCs) and antibiotic resistance genes

Standalone antiSMASH v6.1.1^19^ was used to detect BGCs in the *Pedobacter* genome dataset. Genome assemblies were provided as fasta files, genes were predicted using the integrated Prodigal Pipeline, and BGC detection was limited to clusters larger than 5 kb to minimize the number of incomplete BGCs (--minlength 5000 –genefinding-tool prodigal). Only contigs larger than five kbps were inspected, averaging 72 contigs (1-1481 contigs) per genome. We observed no correlation between the number of detected BGCs and the number of contigs per genome (*R*^2^ = 0.0165; see Fig. S1-1), warranting the robustness of the data. BGC numbers were correlated to the N50 values to detect broken NRPS clusters (Fig. S1-2) and the genome assembly size (Fig. S1-3). Assemblies showing a possible overestimation of BGCs (low N50 values but high numbers of BGCs, Fig. S1-2), underwent a second round of BGC detection after contigs < 10 kb (“Filter Assembled Contigs by Length - v1.2.0” on kbase^42^). After manual curation of the respective antiSMASH outputs, all antiSMASH files were analyzed to extract multimodular NRPS BGCs. These BGCs (n = 32) served as input for BiG-SCAPE to construct a similarity network. BiG-SCAPE was run with default parameters at a cutoff of 0.6, flagging –MIBiG, –mix to include MIBiG reference clusters and a similarity network independent of BGC-type. The Generated Network was visualized with Cytoscape (v3.8.2) using the yFiles organic Layout. The cluster amount and types, specifically for multimodular NRPS and the assembly sizes were extracted and visualized as a heatmap in iTOL^28^.

#### Development of genetic tools for Pedobacter cryoconitis strains

To genetically modify *P. cryoconitis*, we first screened different antibiotics (streptomycin, ampicillin, kanamycin, apramycin, chloramphenicol, and erythromycin) against *P. cryoconitis* PAMC 27485 and *P. cryoconitis* DSM 14825^T^. Only erythromycin (100 µg/ml) and chloramphenicol (150 µg/ml) were effective and selected as markers. A transposon plasmid, pCRYO-01 was modified based on pSAM bt^30^ by assembling PCR-amplified regions upstream of the *P. cryoconitis* PAMC 27485 *gyrB* (AMQ00279.1, 228 bps) and *rpl13* genes (AMQ00867.1, 178 bps) with the erythromycin resistance (*ermR*) and the transposase genes via Gibson Assembly. PCR reactions were carried out using Q5 polymerase (New England Biolabs) as specified by the manufacturer. Conjugation experiments were performed using *E. coli* WM3064/pCRYO-01 and *P. cryoconitis* (overnight cultures) on LB, POM, and R2A (with 5 mM final MgCl_2_) agar with different donor-recipient ratios (1:1, 2:1, 3:1) for 48 h. Cells were scraped off, serially diluted, and plated on R2A agar plates with erythromycin (50, 100, 150, and 200 µg/ml). Erythromycin-resistant colonies were tested for *ermR* presence and plasmid backbone absence by colony PCR. A 3:1 donor-recipient ratio at 23 °C yielded the highest number of transformants. Dilution of the inoculum at 10^− 1^ or 10^− 2^ was optimal for single colonies, with more transformants observed in *P. cryoconitis* PAMC 27485 compared to *P. cryoconitis* DSM 14825^T^. For electroporation, *P. cryoconitis* PAMC 27485 cells (OD 0.4) were made competent by washing 3-times with ice-cold 10% glycerol (centrifugation at 5000 rpm). Aliquots of 50 µl were mixed with pCRYO1. After electroporation (12.5 kV/cm), cells were cultured without antibiotics for 5 h, then plated on R2A with 150 µg/ml erythromycin. Colonies appeared within 3 days, with 100% carrying the transposon cassette, though efficiency was lower than with conjugation. Electroporation of competent cells stored at -80 °C was also possible.

#### Generation of cryopeptin BGC knock-out strain

To experimentally link the production of cryopeptin to its BGC we targeted *crpA* (WP_068400414.1) for deletion by allelic exchange. A suicide plasmid was constructed by digesting pCRYO1 using *Not*I - *Bam*HI and introducing the regions 2 kb upstream and downstream of the *crpA* gene flanking ermR (pCRYO2) using Gibson assembly. Using the previously described conjugation protocol, pCRYO2 was transferred to *P. cryoconitis* PAMC 27485. Initial screening of one hundred conjugants showed that 95 colonies were single crossover recombinants and five were double crossover recombinants. In these colonies, the *crpA* gene was not detectable by PCR (*P. cryoconitis* PAMC 27485 *crpA::ermR*).

## Electronic supplementary material

Below is the link to the electronic supplementary material.

Supplementary Material 1

Supplementary Material 2

## Data Availability

Sequence data that support the findings of this study have been deposited at the National Center for Biotechnology with the primary accession number: PRJNA1019955.

## Materials availability

Plasmids generated in this study are not commercially available.
