# Supplementary material for: A genetically tractable branch of environmental Pedobacter from the phylum Bacteroidota represents a hotspot for natural product discovery
Source: Sci Rep. 2025 Jun 20;15:20106. doi: 10.1038/s41598-025-03955-z (PMC12181424; doi:10.1038/s41598-025-03955-z)
Supplement: Supplementary file 1 — Supplementary Material 1 [file 41598_2025_3955_MOESM1_ESM.docx]

Supplementary Information

A genetically tractable branch of environmental *Pedobacter* from the phylum Bacteroidota represents a hotspot for natural product discovery

Yang Liu^1,§^, Luis Linares-Otoya^1,+,§^, Christian Kersten^3^, Michael Marner^1,2^, Sanja Mihajlovic^2^, Hamdi M. Abdeldayem^1^, Sandra Semmler^2^, Molly C. Beltz^4^, Miguel Vences^5^, Marius Spohn^1,2^, Celine M. Zumkeller^1,2,^* and Till F. Schäberle^1,2,6,*^

1. Institute for Insect Biotechnology with Focus on Natural Product Research; Justus-Liebig-University Giessen, Ohlebergsweg 12, 35392, Giessen, Germany
2. Natural Product Department; Fraunhofer-Institute for Molecular Biology and Applied Ecology (IME), Ohlebergsweg 12, 35392 Giessen, Germany
3. Institute of Pharmaceutical and Biomedical Sciences, Johannes Gutenberg University Mainz, 55128 Mainz, Germany
4. University of Massachusetts Amherst, Department of Environmental Conservation, Amherst, MA, USA
5. Technische Universität Braunschweig, Zoological Institute, Mendelssohnstr. 4, 38106 Braunschweig, Germany
6. German Center for Infection Research (DZIF); Partner Site Giessen-Marburg-Langen, Ohlebergsweg 12, 35392 Giessen, Germany

Lead contact: T.F.S., till.f.schaeberle@agrar.uni-giessen.de

+ current address: Department of Molecular Biology, Princeton University, Princeton, USA.

§ These authors contributed equally to this work.

* Correspondence: Till.F.Schaeberle@agrar.uni-giessen.de (T. F. S.), celine.zumkeller@ime.fraunhofer.de (C.M.Z.)

Contents

1. **Extended Material and Methods**
2. General Analytical Experimental Procedures.
3. Microbial Cultivation.
4. Extraction and Isolation.
5. Marfey’s Analysis.
6. Chemical Synthesis.

- Preparation of S-(2-acetamidoethyl) (R)-2-((tert-butoxycarbonyl)amino)-3-methylbutanethioate (**3.10**)
- Preparation of S-(2-acetamidoethyl) (S)-2-((tert-butoxycarbonyl)amino)-3-methylbutanethioate (**3.11**)
- Preparation of (R)-1-((2-acetamidoethyl)thio)-3-methyl-1-oxobutan-2-aminium chloride (**3.6**)
- Preparation of (S)-1-((2-acetamidoethyl)thio)-3-methyl-1-oxobutan-2-aminium chloride (**3.7**)
- Total synthesis of compound **4** to determine the *R* or *S* configuration of methylbutanoyl residue
- Preparation of 2CT-*L*-leucine-dehydro-valine--arginine(Pbf)-dehydro-valine-l-phenylalanine-NH_2_ (**4.14**)
- Preparation of (2-((S)-5-guanidino-2-(3-methyl-2-((S)-2-((S)-2-methylbutanamido)-3-phenylpropanamido)but-2-enamido)pentanamido)-3-methylbut-2-enoyl)-l-leucine (**4.4**)
- **Scheme S1.** Synthesis of SNAc-Valines
- **Scheme S2**. Synthesis of **4.14** and **4.4**.

1. **Tables**

**Table S 1 Bacterial strains and sources**

**Table S 2 Primers used in this study**

**Table S 3 Plasmids used and generated in this study**

**Table S 4 NMR data of cryopeptins 1-3**

**Table S 5 NMR data of cryopeptins 4-5**

**Table S 6 NMR data of cryopeptins 6-8**

**Table S 57 NMR data of cryopeptins 9-10**

**Table S 68 NMR data of cryopeptins 11-12**

**Table S 9 NMR data of cryopeptins 13-14**

**Table S 710 NMR data comparison in MeOD for synthetic (4.4) and natural compound 4**

**Table S 811 Bioactivity screening. Minimum Inhibitory Concentration (MIC) of 4 and 5**

1. **Figure**

**Figure S1-1.** Plot showing the number of antiSMASH detected BGCs plotted against the number of contigs per respective genome.

**Figure S1-2.** Plot showing the N50 values plotted against the BGC amount. Low N50 genomes containing many BGCs were manually curated to remove broken cluster fragments. Three strains contained broken NRPS clusters, resulting in a lower final BGC count (red circles).

**Figure S1-3.** Plot showing the relationship between the number of detected BGCs and the total assembly size of each respective genome.

**Figure S1-4**. Corason Alignment of BiG-SCAPE identified GCF NRPS – 4 – Cryopeptin. Corason was run at default settings with *crpA* (red) selected as the query gene.

**Figure S1-5 Corason Alignments of multimodular NRPS BGCs present in multiple strains.** Corason was run in default settings, query genes are marked in red. BGC-containing strains are labeled at the ends of the BGCs. NRPS-1 and NRPS-2 (A, B) Show fragments of clusters resulting from the fused, collectively detected BGCs by antiSMASH (marked with *). (C) Corason Alignment of Pedopeptin and putative Isopedopeptin clusters. The Pedopeptin BGC of *P. lusitanus* NL19 is less similar to the others. Coloured genes indicate more modifying genes in the *P. cryoconitis* strains.

**Figure S2**. pCRYO1 transposon mutagenesis plasmid map. Transposase, Ampicillin (*ampR*) and Erythromycin (*ermE*) resistance Genes are represented by yellow arrows. Used primers are indicated in green. Plasmid maintenance and transfer elements are colored blue.

**Figure S3**. Plasmid map of pCRYO2 used for *crpA* deletion. Ampicillin (*ampR*) and Erythromycin (*ermE*) resistance genes are represented by yellow arrows. Region homologous to *P. cryoconitis* cryopeptin BGC are colored red. Plasmid maintenance and transfer elements are colored blue. Used primers are indicated in green.

**Figure S3-2**. PCR of conjugant *P.cryoconitis* PAMC 27485 after allelic exchange *crpA::ermR* . PCR testing the loss of *crpA* in *P.cryoconitis* PAMC 27485 (primers target internal region of *crpA*)

**Figure S4**: A BiG-SCAPE analysis of anti-SMASH detected BGCS in the 8 strains associated to the *P. cryoconitis* clade (Cutoffs for GCFs: 0.6). The node shape indicates the BGC type, and the node color is the strain in the BGC that was detected. Numbers at nodes describe the Big-SCAPE-determined gene cluster family. Besides the shown clusters, 48 singletons were determined. No BGC automatically clusters with any known MiBiG Reference cluster. B: Heat Map illustrating the cosine similarity of the overall BGC composition in the *P. cryoconitis* branch. The calculated tree is based on that similarity and highlights the similar BGC composition in the groups containing for one K2C9 and MP7CTX6 and for another ANJC1 and S3M1.

**Figure S5**. Molecular networking analysis of a *P. cryoconitis* PAMC_27485 and *P. cryoconitis* DSM 14825**^T^** extracts using GNPS. Both strains were cultured in 6 different media (NB, LB, R2A, POM, MYE, TSB) and extracted using 3 different methods (butanol and ethyl acetate liquid-liquid extraction and C18 resin solid phase extraction. Blue and red circles indicate ions that are unique for *P. cryoconitis* PAMC_27485 and *P. cryoconitis* DSM 14825**^T^** respectively. Pink circles indicated ions that are common for both strains. Black circles represent ions found in any of the culture media. Cryopeptin containing clusters are squared.

**Figure S6**. Molecular network analysis of a *P. cryoconitis* PAMC 27485 Wild type and ∆*crpA* strains extracts. Highlighted in orange ions that are present only in WT and not in the deletion mutant. Cryopeptin-containing clusters are numbered and zoomed. Heptapeptidic cryopeptins (K-N) were identified in cluster 4 ([M+2H]^2+^). Cryopeptins A, C, D, E, F, G, H ([M+H]^+^) were identified in cluster 2. Cluster 1 and 3 ions were not structurally characterized. Black circles represent ions that are common for both WT and ∆*crpA* strains.

**Figure S7**. Molecular network containing heptapeptidic cryopeptins from both *P. cryoconitis* PAMC_27485 and *P. cryoconitis* DSM 14825**^T^** extracts. Both strains were cultured in 6 different media (NB, LB, R2A, POM, MYE, TSB) and extracted using 3 different methods (butanol and ethyl acetate liquid-liquid extraction and C18 resin solid phase extraction. Blue and orange circles indicate ions that are unique for *P. cryoconitis* PAMC_27485 and *P. cryoconitis* DSM 14825**^T^** respectively. Purple circles indicated ions that are common for both strains. Structurally characterized cryopeptins are indicated with arrows.

**Figure S8**. Molecular network containing pentapeptidic cryopeptins from both *P. cryoconitis* PAMC 27485 and *P. cryoconitis* DSM 14825**^T^** extracts. Both strains were cultured in 6 different media (NB, LB, R2A, POM, MYE, TSB) and extracted using 3 different methods (butanol and ethyl acetate liquid-liquid extraction and C18 resin solid phase extraction. Blue and red circles indicate ions that are unique for *P. cryoconitis* PAMC_27485 and *P. cryoconitis* DSM 14825**^T^** respectively. Purple circles indicated ions that are common for both strains. Structurally characterized cryopeptins are indicated with arrows.

**Figure S9**. UPLC-HR-ESI-MS analysis of a *P. cryoconitis* DSM 14825**^T^** extract. Extracted ion chromatograms of pedopeptin C and isopedopeptins A and B showing the respective compound peaks with their *m/z* value [M+2H]^2+^. All Δ *m/z* ≤ 0.051

**Figure S10.** UPLC-HR-ESI-MS analysis of a *P. cryoconitis* PAMC 27485 wild type (blue) and Δ*crpA* wild extracts. Extracted ion chromatograms of crypeptins A-N are shown.

**Figure S11-1.** ^1^H NMR spectrum of **1** (DMSO-*d*_6_,700 MHz)

**Figure S11-1-1.** ^1^H NMR spectrum of **1** with assignments (expansion)

**Figure S11-2.** ^13^C NMR spectrum of **1** (DMSO-*d*_6_, 176 MHz)

**Figure S11-2-1.** ^13^C NMR spectrum of **1** with expansion

**Figure S11-2-2.** ^13^C NMR spectrum of **1** with expansion

**Figure S11-3.** COSY spectrum of **1**

**Figure S11-4.** Multiplicity-edited HSQC spectrum of **1**

**Figure S11-5.** HMBC spectrum of **1**

**Figure S11-5-1.** HMBC spectrum of **1** with expansion

**Figure S11-5-2.** HMBC spectrum of **1** with expansion

**Figure S11-6.** UPLC-HR-ESI-MS spectrum of **1**

**Figure S12-1.** ^1^H NMR spectrum of **2** (DMSO-*d*_6_,700 MHz)

**Figure S12-1-1.** ^1^H NMR spectrum of **2** with expansion

**Figure S12-2.** ^13^C NMR spectrum of **2** (DMSO-*d*_6_, 176 MHz)

**Figure S12-2-1.** ^13^C NMR spectrum of **2** with expansion

**Figure S12-2-2.** ^13^C NMR spectrum of **2** with expansion

**Figure S12-3.** COSY spectrum of **2**

**Figure S12-3-1.** COSY spectrum of **2** with expansion

**Figure S12-4.** Multiplicity-edited HSQC spectrum of **2**

**Figure S12-5.** HMBC spectrum of **2**

**Figure S12-5-1.** HMBC spectrum of **2** with expansion

**Figure S12-5-2.** HMBC spectrum of **2** with expansion

**Figure S12-6.** UPLC -HR-ESI-MS spectrum of **2**

**Figure S13-1.** ^1^H NMR spectrum of **3** (DMSO-*d*_6_,700 MHz)

**Figure S13-1-1.** ^1^H NMR spectrum of **3** with expansion

**Figure S13-2.** ^13^C NMR spectrum of **3** (DMSO-*d*_6_, 176 MHz)

**Figure S13-2-1.** ^13^C NMR spectrum of **3** with expansion

**Figure S13-2-2.** ^13^C NMR spectrum of **3** with expansion

**Figure S13-3.** COSY spectrum of **3**

**Figure S13-4.** Multiplicity-edited HSQC spectrum of **3**

**Figure S13-5.** HMBC spectrum of **3**

**Figure S13-5-1.** HMBC spectrum of **3** with expansion

**Figure S13-5-2.** HMBC spectrum of **3** with expansion

**Figure S13-6.** UPLC-HR-ESI-MS spectrum of **3**

**Figure S14-1.** ^1^H NMR spectrum of **4** (DMSO-*d*_6_,700 MHz)

**Figure S14-1-1.** ^1^H NMR spectrum of **4** with expansion

**Figure S14-2.** ^13^C NMR spectrum of **4** (DMSO-*d*_6_, 176 MHz)

**Figure S14-2-1.** ^13^C NMR spectrum of **4** with expansion

**Figure S14-2-2.** ^13^C NMR spectrum of **4** with expansion

**Figure S14-3.** COSY spectrum of **4**

**Figure S14-4.** Multiplicity-edited HSQC spectrum of **4**

**Figure S14-5.** HMBC spectrum of **4**

**Figure S14-5-1.** HMBC spectrum of **4** with expansion

**Figure S14-5-2.** HMBC spectrum of **4** with expansion

**Figure S14-6.** UPLC-HR-ESI-MS spectrum of **4**

**Figure S15-1.** ^1^H NMR spectrum of **5** (DMSO-*d*_6_,700 MHz)

**Figure S15-1-1.** ^1^H NMR spectrum of **5** with expansion

**Figure S15-2.** ^13^C NMR spectrum of **5** (DMSO-*d*_6_, 176 MHz)

**Figure S15-2-1.** ^13^C NMR spectrum of **5** with expansion

**Figure S15-2-2.** ^13^C NMR spectrum of **5** with expansion

**Figure S15-3.** COSY spectrum of **5.**

**Figure S15-4.** Multiplicity-edited HSQC spectrum of **5**

**Figure S15-5.** HMBC spectrum of **5**

**Figure S15-5-1.** HMBC spectrum of **5** with expansion

**Figure S15-5-2.** HMBC spectrum of **5** with expansion

**Figure S15-6.** UPLC-HR-ESI-MS spectrum of **5**

**Figure S16-1.** ^1^H NMR spectrum of **6** (DMSO-*d*_6_,700 MHz)

**Figure S16-1-1.** ^1^H NMR spectrum of **6** with expansion

**Figure S16-2.** ^13^C NMR spectrum of **6** (DMSO-*d*_6_, 176 MHz)

**Figure S16-2-1.** ^13^C NMR spectrum of **6** with expansion

**Figure S16-2-2.** ^13^C NMR spectrum of **6** with expansion

**Figure S16-3.** COSY spectrum of **6**

**Figure S16-4.** Multiplicity-edited HSQC spectrum of **6**

**Figure S16-5.** HMBC spectrum of **6**

**Figure S16-5-1.** HMBC spectrum of **6** with expansion

**Figure S16-6.** UPLC-HR-ESI-MS spectrum of **6**

**Figure S17-1.** ^1^H NMR spectrum of **7** (DMSO-*d*_6_,700 MHz)

**Figure S17-1-1.** ^1^H NMR spectrum of **7** with expansion

**Figure S17-2.** ^13^C NMR spectrum of **7** (DMSO-*d*_6_, 176 MHz)

**Figure S17-2-1.** ^13^C NMR spectrum of **7** with expansion

**Figure S17-2-2.** ^13^C NMR spectrum of **7** with expansion

**Figure S17-3.** COSY spectrum of **7**

**Figure S17-4.** Multiplicity-edited HSQC spectrum of **7**

**Figure S17-5.** HMBC spectrum of **7**

**Figure S17-5-1.** HMBC spectrum of **7** with expansion

**Figure S17-5-2.** HMBC spectrum of **7** with expansion

**Figure S17-6.** UPLC-HR-ESI-MS spectrum of **7**

**Figure S18-1.** ^1^H NMR spectrum of **8** (DMSO-*d*_6_,700 MHz)

**Figure S18-1-1.** ^1^H NMR spectrum of **8** with expansion

**Figure S18-2.** ^13^C NMR spectrum of **8** (DMSO-*d*_6_, 176 MHz)

**Figure S18-2-1.** ^13^C NMR spectrum of **8** with expansion

**Figure S18-2-2.** ^13^C NMR spectrum of **8** with expansion

**Figure S18-3.** COSY spectrum of **8.**

**Figure S18-4.** Multiplicity-edited HSQC spectrum of **8**

**Figure S18-5.** HMBC spectrum of **8**

**Figure S18-5-1.** HMBC spectrum of **8** with expansion

**Figure S18-5-2.** HMBC spectrum of **8** with expansion

**Figure S18-6.** UPLC-HR-ESI-MS spectrum of **8**

**Figure S19-1.** ^1^H NMR spectrum of **9** (DMSO-*d*_6_,700 MHz)

**Figure S19-1-1.** ^1^H NMR spectrum of **9** with expansion

**Figure S19-2.** ^13^C NMR spectrum of **9** (DMSO-*d*_6_, 176 MHz)

**Figure S19-2-1.** ^13^C NMR spectrum of **9** with expansion

**Figure S19-2-2.** ^13^C NMR spectrum of **9** with expansion

**Figure S19-3.** COSY spectrum of **9**

**Figure S19-4.** Multiplicity-edited HSQC spectrum of **9**

**Figure S19-5.** HMBC spectrum of **9**

**Figure S19-5-1.** HMBC spectrum of **9** with expansion

**Figure S19-5-2.** HMBC spectrum of **9** with expansion

**Figure S19-6.** UPLC-HR-ESI-MS spectrum of **9**

**Figure S20-1.** ^1^H NMR spectrum of **10** (DMSO-*d*_6_,700 MHz)

**Figure S20-1-1.** ^1^H NMR spectrum of **10** with expansion

**Figure S20-2.** ^13^C NMR spectrum of **10** (DMSO-*d*_6_, 176 MHz)

**Figure S20-2-1.** ^13^C NMR spectrum of **10** with expansion

**Figure S20-2-2.** ^13^C NMR spectrum of **10** with expansion

**Figure S20-3.** COSY spectrum of **10**

**Figure S20-4.** Multiplicity-edited HSQC spectrum of **10**

**Figure S20-5.** HMBC spectrum of **10**

**Figure S20-5-1.** HMBC spectrum of **10** with expansion

**Figure S20-5-2.** HMBC spectrum of **10** with expansion

**Figure S20-6.** UPLC-HR-ESI-MS spectrum of **10**

**Figure S21-1.** ^1^H NMR spectrum of **11** (DMSO-*d*_6_,700 MHz)

**Figure S21-1-1.** ^1^H NMR spectrum of **11** with expansion

**Figure S21-2.** ^13^C NMR spectrum of **11** (DMSO-*d*_6_, 176 MHz)

**Figure S21-2-1.** ^13^C NMR spectrum of **11** with expansion

**Figure S21-2-2.** ^13^C NMR spectrum of **11** with expansion

**Figure S21-3.** COSY spectrum of **11.**

**Figure S21-4.** Multiplicity-edited HSQC spectrum of **11**

**Figure S21-5.** HMBC spectrum of **11**

**Figure S21-5-1.** HMBC spectrum of **11** with expansion

**Figure S21-5-2.** HMBC spectrum of **11** with expansion

**Figure S21-6.** UPLC-HR-ESI-MS spectrum of **11**

**Figure S21-7.** MS/MS fragmentation pattern of **11**

**Figure S22-1.** ^1^H NMR spectrum of **12** (DMSO-*d*_6_,700 MHz)

**Figure S22-1-2.** ^1^H NMR spectrum of **12** with expansion

**Figure S22-2.** ^13^C NMR spectrum of **12** (DMSO-*d*_6_, 176 MHz)

**Figure S22-2-1.** ^13^C NMR spectrum of **12** with expansion

**Figure S22-2-2.** ^13^C NMR spectrum of **12** with expansion

**Figure S22-3.** COSY spectrum of **12**

**Figure S22-4.** Multiplicity-edited HSQC spectrum of **12**

**Figure S22-5.** HMBC spectrum of **12**

**Figure S22-5-1.** HMBC spectrum of **12** with expansion

**Figure S22-5-2.** HMBC spectrum of **12** with expansion

**Figure S22-6.** UPLC-HR-ESI-MS spectrum of **12**

**Figure S22-7.** MS/MS fragmentation pattern of **11**

**Figure S23-1.** ^1^H NMR spectrum of **13** (DMSO-*d*_6_,700 MHz)

**Figure S23-1-1.** ^1^H NMR spectrum of **13** with expansion

**Figure S23-2.** ^13^C NMR spectrum of **13** (DMSO-*d*_6_, 176 MHz)

**Figure S23-2-1.** ^13^C NMR spectrum of **13** with expansion

**Figure S23-2-2.** ^13^C NMR spectrum of **13** with expansion

**Figure S23-3.** COSY spectrum of **13**

**Figure S23-4.** Multiplicity-edited HSQC spectrum of **13**

**Figure S23-5.** HMBC spectrum of **13**

**Figure S23-5-1.** HMBC spectrum of **13** with expansion

**Figure S23-5-2.** HMBC spectrum of **13** with expansion

**Figure S23-6.** UPLC-HR-ESI-MS spectrum of **13**

**Figure S24-1.** ^1^H NMR spectrum of **14** (DMSO-*d*_6_,700 MHz)

**Figure S24-1-1.** ^1^H NMR spectrum of **14** with expansion

**Figure S24-2.** ^13^C NMR spectrum of **14** (DMSO-*d*_6_, 176 MHz)

**Figure S24-2-1.** ^13^C NMR spectrum of **14** with expansion

**Figure S24-2-2.** ^13^C NMR spectrum of **14** with expansion

**Figure S24-3.** COSY spectrum of **14.**

**Figure S24-4.** Multiplicity-edited HSQC spectrum of **14**

**Figure S24-5.** HMBC spectrum of **14**

**Figure S24-5-1.** HMBC spectrum of **14** with expansion

**Figure S24-5-2.** HMBC spectrum of **14** with expansion

**Figure S24-6.** UPLC-HR-ESI-MS spectrum of **14**

**Figure S25.** Structures of compounds **1**-**5**

**Figure S26**. Key HMBC correlations of compounds **1**-**5**

**Figure S27**. Structures of compounds **6**-**10**

**Figure S28**. Key HMBC correlations of compounds **6**-**10**

**Figure S29**. Structures of compounds **11**-**14**

**Figure S30.** Key HMBC correlations of compounds **11**-**14**

**Figure S31**. UPLC-HR-ESI-MS analysis of *D*/*L*-FDVA-Leu

**Figure S32**. UPLC-HR-ESI-MS analysis of *D*/*L*-FDVA-Phe

**Figure S33**. UPLC-HR-ESI-MS analysis of *D*/*L*-FDVA-Arg

**Figure S34.** Structures of *R*- and *S*-NAc-valines

**Figure S35-1-1.** ^1^H NMR spectra of **3.10** in CDCl_3_.

**Figure S35-1-2.** ^13^C NMR spectra of **3.10** in CDCl_3_.

**Figure S35-2-1.** ^1^H NMR spectra of **3.11** in CDCl_3_.

**Figure S35-2-2.** ^13^C NMR spectra of **3.11** in CDCl_3_.

**Figure S35-3-1.** ^1^H NMR spectra of **3.6** in DMSO-*d*_6_.

**Figure S35-3-2.** ^13^C NMR spectra of **3.6** in DMSO-*d*_6_.

**Figure S35-4-1.** ^1^H NMR spectra of **3.7** in DMSO-*d*_6_.

**Figure S35-4-2.** ^13^C NMR spectra of **3.7** in DMSO-*d*_6_.

**Figure S36-1-1.** ^1^H NMR spectra of **4.4** in MeOD.

**Figure S36-1-2.** ^13^C NMR spectra of **4.4** in MeOD.

**Figure S36-2-1.** HMBC spectra of **4.4** in MeOD.

**Figure S36-2-2.** HMBC spectra (expansion) of **4.4** in MeOD

**Figure S37.** MS/MS fragmentation of **4.4** and fragmentation pattern.

**Figure S38**. Original NMR spectra of natural, isolated compound **4** in MeOD (600 MHz, 150 MHz).

# Extended Material and Methods

## General Analytical Experimental Procedures.

The 1D and 2D NMR spectra were recorded in CD_3_OD, or DMSO‑*d*_6_ using Bruker Avance Neo 700 MHz spectrometers equipped with a Prodigy cryoprobe (Brucker, Ettlingen, Germany). The UPLC-HR-ESI-MS data were recorded on MicrOTOF Q, an Agilent Infinity 1290 UPLC system (Agilent, Santa Clara, CA, USA) equipped with an Acquity UPLC BEH C18 1.7 μm (2.1 × 100 mm) column and an Acquity UPLC BEH C18 1.7 μm VanGuard Pre-Column (2.1 × 5 mm; both columns purchased from Waters, Eschborn, Germany) coupled to a DAD detector and a micrOTOFQ II mass spectrometer (Bruker Daltonics, Bremen, Germany) with an electrospray ionization source was employed. The UPLC system was operated using a gradient (A: H_2_O, 0.1% formic acid; B: acetonitrile, 0.1% formic acid; flow: 600 μL/min), and the column oven temperature was set to 45 °C. Method 1: 0 - 0.8 min: 95% A; 0.8 -18.70 min: 95% A - 4.75% A; 18.80 – 23 min: 5% A; 23.10 – 25 min: 95% A. Method 2: 0 -10 min: 95% A; 10 - 40 min: 95% A - 5% A; 40 – 50 min: 5% A; 50 – 60 min: 95% A. MS data was acquired over a range from 100–3000 m/z in positive mode. Auto MS/MS fragmentation was achieved with rising. Collision energy (35–50 keV over a gradient from 500–2000 m/z) with a frequency of 4 Hz for all ions over a threshold of 100. HPLC was performed using a Shimadzu HPLC system (Shimadzu Deutschland GmbH, Duisburg, Germany) for analysis (EC 250/4.6 Nucleodur C18 GravitySB‑, 5 μm; Macherey-Nagel, Düren, Germany), and for semi-preparative purification (VP 250/10 Nucleodur C18 Gravity-SB, 5 μm; Macherey-Nagel, Düren, Germany). Generated analytical data were analysed using the software Data Analysis. For molecular network generation MS/MS spectrums were converted to the mzXML format and transferred to the GNPS server ^1^. A minimum cosine score of 0.7 and a minimum of three matched fragment ions was chosen for molecular network generation. Cytoscape^2^ 3.8 software package was used for network visualization.

## Microbial Cultivation:

Both strains *P. cryoconitis* PAMC 27485 (Polar and Alpine Microbial Collection (PAMC)- Korea Polar Research Institute (KOPRI) laboratory (South Korea)) and *P. cryoconitis* DSM 14825^T^ German Collection of Microorganisms and Cell Cultures (DSMZ, Germany) were cultivated in six different media MYE (glucose 10 g, yeast extract 3 g, malt extract 3 g, peptone 5 g, distill water 1 L), TSB (Peptone from Casein 17 g, Peptone from Soy 3 g, D-Glucose 2.5g, NaCl 5 g, di-Potassium hydrogen phosphate 2.5 g, distill water 1 L), R2A (Yeast extract 0.5 g, Proteose Peptone 0.5 g, Casein hydrolysate 0.5 g, D-Glucose 0.5 g, Starch 0.5 g, Sodium pyruvate 0.3 g, di-Potassium hydrogen phosphate 0.3 g, Magnesium sulphate heptahydrate 0.05 g, distill water 1 L, pH 7.2), LB (peptone 10 g, yeast extract 5 g, distill water 1 L), NB (Peptone from Soy 5 g, Malt extract 3 g, NaCl 5 g) and POM (Peptone from Soy 23.8 g, Yeast extract 8.8 g, Ammonium chloride 0.7 g). Autoclaving at 121 °C for 15 minutes was performed to sterilize all media. LB supplemented with a diaminopimelic acid (DAP) (0.3 mM) was used as *E. coli* WM3064 cultivation medium. Ampicillin (100 µg/mL) was used if required.

Small scale cultivations were performed in 300 ml EK flasks with 150 ml medium (NB, LB, R2A, TSB, POM, MYE) at 20 °C and 120 rpm. *P. cryoconitis* PAMC_27485::∆*crpA* and WT strains were cultured in R2A medium for 2 days at 20 ºC and 120 rpm. Large-scale fermentation (60 L) of *P. cryoconitis* PAMC 27485 was performed in 2 L flasks which contained 1 L of NB medium and were incubated at 20 °C and 120 rpm for 4 days.

## Extraction and Isolation.

The small-scale cultivations were split into 3 equal parts of 50 mL; each part was extracted using a different method. Three extraction methods were used: liquid-liquid extraction using ethyl acetate (1:1 solvent to sample ratio) and butanol (2:1 solvent to sample ratio). Two rounds of extraction for each sample were conducted, and the organic phase was collected from the separating funnel to round bottom flasks for vacuum-aided evaporation. Additionally, a solid phase extraction (SPE) using a reversed-phase C18 column (10 g C18 in each column, from Interchim) was used. Cell-free cultures were loaded into a C18 column, followed by a 2-minute washing step using 5% acetonitrile: Water acidified with 0.1% Formic acid water to exclude salts and sugars (25 mL/min). Elution steps started with 2 minutes of 80%, followed by 100% ACN acidified with 0.1% formic acid for 3 minutes. Combined organic extracts were dried using a rotary vacuum evaporator. Resulting crude extracts were then transferred to glass vials, weights were recorded, and stored at -20 ℃. As a negative control, axenic media samples were prepared in the same way. Samples (1mg/mL) were analyzed by HPLC-HR-ESI-MS/MS on a micrOTOF-Q mass spectrometer. *P. cryoconitis* PAMC_27485::∆*crpA* and WT strains cultures (supernatant) were extracted using a C18 SPE. The 60 L culture was extracted with amberlite XAD 16 (5L of amberlite XAD 16 20-60 mesh was used, from Sigma) and eluted with 20% MeOH (E1), 50% MeOH (E2), 80% MeOH (E3), 100% MeOH (E4) and 100% acetonitrile with 0.1% formic acid (E5). All of the elutions were analyzed by UPLC-HR-ESI-MS, then elution E2 and E3 were combined and named as E2, elutions E4 and E5 were combined and named as E4. Each obtained elutions E2 and E4 were fractionated by reversed phase flash chromatography (Interchim Puriflash 4125 chromatography system with Puriflash C18-AQ30 μm F0120 column) with an elution gradient starting from 5% acetonitrile/H_2_O with 0.1% formic acid to 100% acetonitrile with 0.1% formic acid over 60 min, respectively. 10 fractions were obtained from E2 after flash chromatography, fraction E2-F4 contained the target compounds guiding by molecular networking, further separated with flash once more (Interchim Puriflash 4125 chromatography system with Puriflash C18-AQ30 μm F0080 column), the subfractions E2-F4-10 and E2-F4-12 underwent with sephadex LH20, and obtained fractions E2-F4-10-SF2 and E2-F4-12-SF2 finally purified by HPLC to generate compounds **1**-**5 (3.5 mg, 2.6 mg, 2.2 mg, 26.4 mg, 29.2 mg, respectively)**. And fractions E2-F4-13 and E2-F4-14 directly purified by HPLC and yield compounds **6**-**8 (6.7 mg, 3.2 mg, 2.9 mg, respectively)**. Fractions E2-F7 went through sephadex LH 20, and the subfraction E2-F7-SF3 purified by HPLC and generated compounds **9**-**10 (1.9 mg and 3.1 mg)**. Elution E4 after flash chromatography obtained 4 subfractions, fraction E4-F2 submitted to sephadex LH 20 and the subfraction E4-F2-SF2 purified by HPLC to yield compounds **11**-**14 (2.7mg, 2.1mg, 1.8 mg, 1.6 mg, respectively)**.

## Marfey’s Analysis.

Preparing the reference amino acids: A 5 mM stock solution of each amino acid (*l*-/*d*-Leucine, *l*-/*d*-Arginine, *l*-/*d*-Phenylalanine; Sigma Aldrich) in H_2_O was prepared. 20 µL 1 M NaHCO_3_ and 50 µL 7 mM lFDVA (Sigma Aldrich) in acetone were added to each 50 µL amino acid stock solution. Those solutions were stirred at 40 °C for 3 h and then quenched by adding 20 µL 1 M HCl. After evaporation to dryness, the residues were dissolved in 40 µL DMSO and analyzed by UPLC-HR-ESI-MS. Preparing the sample: 0.1 µmol of compound 4 was dissolved in 200 µL 6 M DCl in D_2_O and stirred at 160 °C for 7 h. After concentrating the solution under reduced pressure, the residue was dissolved in 200 µL H_2_O. Then 100 µL 1 M NaHCO_3_ and 200 µL 7 mM lFDVA in acetone were added. After stirring for 3 h at 40 °C, the solution was quenched by adding 100 µL 1 M HCl. After evaporation to dryness, the residue was dissolved in 50 µL DMSO and analyzed by UPLC-HR-ESI-MS (see Figure S31, S32 and S33).

## Chemical Synthesis.

As mimics for testing the dehydrogenation process, both SNAc-Valine enantiomers, as shown in **Scheme S1** were synthesized, following standard peptide coupling and Bocdeprotection procedures. The EDC/HOAt-mediated coupling was carried out in solution and **3.10** and **3.11** were obtained with a 68% and 42% yield, respectively. The deprotection was achieved quantitatively for **3.6** and with 93% for **3.7**. This affords the *R*-enantiomer **3.6** with an overall yield of 68% and 39% for the *S*-enantiomer **3.7**.

**Scheme S1.** Synthesis of SNAc-Valines.

### Preparation of *S*-(2-acetamidoethyl) (*R*)-2-((tert-butoxycarbonyl)amino)-3-methylbutanethioate (3.10)

*N*-Boc-d-valine (511 mg, 2.35 mmol), EDC · HCl (496 mg, 2.59 mmol) and HOAt (358 mg, 2.63 mmol) were dissolved in anhydrous DCM. N-Acetlycysteamine (95%, 250 µL, 2.23 mmol) was added, followed by triethylamine (359 µL, 2.59 mmol). The mixture was stirred at room temperature for 25 h. After dilution with EA, the organic phase was washed three times with citric acid (10% w/v), once with sat. NaHCO_3_ solution and once with brine. The organic layer was dried over MgSO_4_ and concentrated *in vacuo*. Purification by semi preparative HPLC (32-45% AcCN + 0.1% FA, NUCLEODUR® C18 Gravity SB, 3 µm, 250 x 10 mm, flow rate: 3 mL/min) yielded **3.10** as a colorless syrup (506 mg, 1.59 mmol, 68%).

**^1^H-NMR** (CDCl_3_, 400 MHz): δ_H_ [ppm] = 5.93 (s, 1H, N*H*-Ac), 4.98 (d, 1H, *J* = 9.0 Hz, N*H-*Boc), 4.23 (dd, 1H, *J* = 8.9, 4.6 Hz, α-C*H* Val), 3.46 (ddd, 1H, *J* = 13.2, 13.2, 6.5 Hz, C*H*_2_-NH a), 3.38 (ddd, 1H, *J* = 13.1, 13.1, 6.2 Hz, C*H*_2_-NH b), 3.04 (ddd, 2H, *J* = 6.4, 6.4, 2.9 Hz, S-C*H*_2_), 2.29-2.19 (m, 1H, β-C*H* Val), 1.95 (s, 3H, C*H*_3_ Ac), 1.45 (s, 9H, C*H*_3_ Boc), 0.99 (d, 3H, *J* = 6.8 Hz, γ-C*H*_3_ a Val), 0.87 (d, 3H, *J* = 6.8 Hz, γ-C*H*_3_ a Val).

**^13^C-NMR** (CDCl_3_, 100 MHz): δ_C_ [ppm] = 201.9 (*C*O Val), 170.5 (*C*O Ac), 155.8 (*C*O Boc), 80.6 (*C*_quart_ Boc), 65.8 (α-*C*H Val), 39.5 (*C*H_2_-NH), 30.9 (β-*C*H Val), 28.50 (S-*C*H_2_), 28.45 (*C*H_3_ Boc), 23.3 (*C*H_3_ Ac), 19.5 (γ-*C*H_3_ a Val), 17.1 (γ-*C*H_3_ b Val).

Additional found signals: δ_H_ [ppm] = 8.08 (FA), 5.29 (DCM). δ_C_ [ppm] = /

**UPLC-HR-ESI-MS** *m/z* calcd for C_14_H_27_N_2_O_4_S: 319.1686 [M+H]^+^; found: 319.1688 [M+H]^+^

### Preparation of *S*-(2-acetamidoethyl) (*S*)-2-((tert-butoxycarbonyl)amino)-3-methylbutanethioate (3.11)

*N*-Boc-l-valine (512 mg, 2.36 mmol), EDC · HCl (499 mg, 2.60 mmol) and HOAt (358 mg, 2.63 mmol) were dissolved in anhydrous DCM. N-Acetlycysteamine (95%, 262 µL, 2.34 mmol) was added, followed by triethylamine (359 µL, 2.59 mmol). The mixture was stirred at room temperature for 22 h. After dilution with EA, the the organic phase was washed three times with citric acid (10% w/v), once with sat. NaHCO_3_ solution and once with brine. The organic layer was dried over MgSO_4_ and concentrated *in vacuo*. Purification by semi preparative HPLC (32-45% ACN + 0.1% FA, NUCLEODUR® C18 Gravity SB, 3 µm, 250 x 10 mm, flow rate: 3 mL/min) yielded **3.11** as a colorless syrup (312 mg, 0.980 mmol, 42%).

**^1^H-NMR** (CDCl_3_, 400 MHz): δ_H_ [ppm] = 6.00 (s, 1H, N*H*-Ac), 4.98 (d, 1H, *J* = 8.7 Hz, N*H*-Boc), 4.23 (dd, 1H, *J* = 8.9, 4.8 Hz, α-C*H* Val), 3.47 (ddd, 1H, *J* = 13.0, 13.0, 6.4 Hz, C*H*_2_-NH a), 3.39 (ddd, 1H, *J* = 13.0, 13.0, 6.3 Hz, C*H*_2_-NH b), 3.04 (ddd, 2H, *J* = 6.3, 6.3, 3.6 Hz, S-C*H*_2_), 2.30-2.19 (m, 1H, β-C*H* Val), 1.97 (s, 3H, C*H*_3_ Ac), 1.46 (s, 9H, C*H*_3_ Boc), 0.99 (d, 3H, *J* = 6.8 Hz, γ-C*H*_3_ a Val), 0.88 (d, 3H, *J* = 6.8 Hz, γ-C*H*_3_ a Val).

**^13^C-NMR** (CDCl_3_, 100 MHz): δ_C_ [ppm] = 201.9 (*C*O Val), 170.7 (*C*O Ac), 155.8 (*C*O Boc), 80.6 (*C*_quart_ Boc), 65.8 (α-*C*H Val), 39.6 (*C*H_2_-NH), 30.9 (β-*C*H Val), 28.5* (S-*C*H_2_), 28.5 (*C*H_3_ Boc), 23.2 (*C*H_3_ Ac), 19.5 (γ-*C*H_3_ a Val), 17.2 (γ-*C*H_3_ b Val).

Additional found signals: δ_H_ [ppm] = 8.07 (FA), 5.30 (DCM). δ_C_ [ppm] = /

**UPLC-HR-ESI-MS** *m/z* calcd for C_14_H_27_N_2_O_4_S: 319.1686 [M+H]^+^; found: 319.1687 [M+H]^+^

### Preparation of (*R*)-1-((2-acetamidoethyl)thio)-3-methyl-1-oxobutan-2-aminium chloride (3.6)

Compound **3.10** (480 mg, 1.51 mmol) was dissolved in 4 M HCl in 1,4-dioxane (30 mL) and stirred at room temperature for 70 min in a sealed flask. An argon stream removed residual HCl and the solvent was removed *in vacuo*. Product **3.6** was obtained as a colorless solid (386 mg, 1.52 mmol, quantitively) without further purification.

**^1^H-NMR** (DMSO-*d*_6_, 400 MHz): δ_H_ [ppm] = 8.63 (bd, 3H, N*H*_3_^+^), 8.18 (t, 1H, *J* = 5.8 Hz, N*H*-Ac), 4.08 (t, 1H, *J* = 5.1 Hz, α-C*H* Val), 3.22 (ddd, 2H, *J* = 6.3, 6.3, 6.3 Hz, C*H*_2_-NH) 3.08 (ddd, 1H, *J* = 13.4, 6.7, 6.7 Hz, S-C*H*_2_ a), 3.02 (ddd, 1H, *J* = 13.2, 6.5, 6.5 Hz, S-C*H*_2_ b), 2.26-2.13 (m, 1H, β-C*H* Val), 1.79 (s, 3H, C*H*_3_ Ac), 0.98 (d, 3H, *J* = 7.4 Hz, γ-C*H*_3_ a Val), 0.96 (d, 3H, *J* = 7.3 Hz, γ-C*H*_3_ a Val).

**^13^C-NMR** (DMSO-*d*_6_, 100 MHz): δ_C_ [ppm] = 196.0 (*C*O Val), 169.4 (*C*O Ac), 63.4 (α-*C*H Val), 37.8 (*C*H_2_-NH), 30.0 (β-*C*H Val), 28.4 (S-*C*H_2_), 22.5 (*C*H_3_ Ac), 18.1 (γ-*C*H_3_ a Val), 17.6 (γ-*C*H_3_ b Val).

Additional found signals: δ_H_ [ppm] = 4.49, 3.56 (1,4-dioxane). δ_C_ [ppm] = 66.4 (1,4-dioxane).

**UPLC-HR-ESI-MS** *m/z* calcd for C_9_H_19_N_2_O_2_S: 219.1162 [M+H]^+^; found: 219.1164 [M+H]^+^

### Preparation of (*S*)-1-((2-acetamidoethyl)thio)-3-methyl-1-oxobutan-2-aminium chloride (3.7)

The solution of Compound **3.11** (166 mg, 0.521 mmol) in 4 M HCl in 1,4-dioxane (10 mL) was stirred at room temperature for 60 min in a sealed flask. An argon stream removed residual HCl and the solvent was removed *in vacuo*. Product **3.7** was obtained as a colorless solid (123 mg, 0.483 mmol, 93%) without further purification.

**^1^H-NMR** (DMSO-*d*_6_, 400 MHz): δ_H_ [ppm] = 8.67 (bd, 3H, N*H*_3_^+^), 8.20 (t, 1H, *J* = 5.4 Hz, N*H*-Ac), 4.06 (t, 1H, *J* = 5.0 Hz, α-C*H* Val), 3.22 (ddd, 2H, *J* = 6.2, 6.2, 6.2 Hz, C*H*_2_-NH) 3.07 (ddd, 1H, *J* = 13.3, 6.5, 6.5 Hz, S-C*H*_2_ a), 3.01 (ddd, 1H, *J* = 13.4, 6.8, 6.8 Hz, S-C*H*_2_ b), 2.25-2.13 (m, 1H, β-C*H* Val), 1.79 (s, 3H, C*H*_3_ Ac), 0.98 (d, 3H, *J* = 6.6 Hz, γ-C*H*_3_ a Val), 0.96 (d, 3H, *J* = 6.7 Hz, γ-C*H*_3_ a Val).

**^13^C-NMR** (DMSO-*d*_6_, 100 MHz): δ_C_ [ppm] = 195.9 (*C*O Val), 169.4 (*C*O Ac), 63.5 (α-*C*H Val), 37.8 (*C*H_2_-NH), 30.0 (β-*C*H Val), 28.4 (S-*C*H_2_), 22.5 (*C*H_3_ Ac), 18.0 (γ-*C*H_3_ a Val), 17.6 (γ-*C*H_3_ b Val).

Additional found signals: δ_H_ [ppm] = 4.66. δ_C_ [ppm] = /

**UPLC-HR-ESI-MS** *m/z* calcd for C_9_H_19_N_2_O_2_S: 219.1162 [M+H]^+^; found: 219.1163 [M+H]^+^

### Total synthesis of compound 4 to determine the *R* or *S* configuration of methylbutanoyl residue

To disclose the stereogenic center of the side chain moiety, which is a methylbutanoyl residue with either an *R* or *S* configuration, linear pentapeptide **4.14** was prepared, after which the batch was split (Scheme S2). That allows for a quick and easy derivatization with different fatty acids. The easier obtainable *S*-methylbutanoic acid was the first fatty acid to be used for derivatization. After the coupling and subsequent cleavage of the peptide from the resin **4.4** was obtained with 17% overall yield.

**Scheme S2**. Synthesis of **4.14** and **4.4**.

### Preparation of 2CT-*L*-leucine-dehydro-valine-*L*-arginine(Pbf)-dehydro-valine-*L*-phenylalanine-NH_2_ (4.14)

H-*L*-Ile-2-chlorotrityl resin (n = 0.74 mmol/g, 2.106 g, 1.558 mmol) was swelled in DMF for 30 min. The solvent was removed and a solution of Fmoc-2,3-dehydrovaline-OH (1.577 g, 4.674 mmol) and HATU (1.720 g, 4.524 mmol) in DMF was added. DIPEA (1590 µL, 9.350 mmol) and more DMF followed. The mixture was agitated for 1 h, succeeded by Fmoc-deprotection. Fmoc-l-arginine(Pbf)-OH (3.037 g, 4.681 mmol) and HATU (1.721 g, 4.526 mmol) were dissolved in DMF and added to the drained resin, followed by DIPEA (1590 µL, 9.350 mmol) and more DMF. The mixture was agitated for 1 h. After Fmoc-deprotection, Fmoc-2,3-dehydrovaline-OH (1.577 g, 4.674 mmol) and HATU (1.718 g, 4.518 mmol) dissolved in DMF, were added, followed by DIPEA (1590 µL, 9.350 mmol) and more DMF. The mixture was agitated for 3 h, after which the coupling was repeated due to the incompleteness of the reaction. Fmoc-2,3-dehydrovaline-OH (0.571 g, 1.69 mmol) and HATU (0.624 g, 1.641 mmol) were added as a solution in DMF, followed by DIPEA (575 µL, 3.38 mmol) and more DMF. The mixture was agitated for 1 h, after which full conversion was observed as indicated by LC-MS. After Fmoc-deprotection, Fmoc-l-phenylalanine-OH (1.816 g, 4.688 mmol) and HATU (1.720 g, 4.524 mmol) dissolved in DMF, were added, followed by DIPEA (1590 µL, 9.350 mmol) and more DMF. The mixture was agitated for 2 h, after which the coupling was repeated. Fmoc-l-phenylalanine-OH (1.811 g, 4.675 mmol) and HATU (1.719 g, 4.521 mmol) dissolved in a bit DMF, were added to the resin, followed by DIPEA (1590 µL, 9.350 mmol) and more DMF. The mixture was agitated for 2 h. After Fmoc-deprotection the supernatant was drained, the resin was washed three times with DMF, two times each with isopropanol and *n*-heptane. It was sucked dry and half of resin **4.14** was taken for storage. This was dried *in vacuo* for 18 h, flushed with argon and stored at -8 °C. The rest of resin**4.14** was directly used in the next step.

### Preparation of (2-((*S*)-5-guanidino-2-(3-methyl-2-((*S*)-2-((*S*)-2-methylbutanamido)-3-phenylpropanamido)but-2-enamido)pentanamido)-3-methylbut-2-enoyl)-*L*-leucine (4.4)

Resin **4.14** (0.779 mmol) was swelled in DMF for 30 min. The solvent was drained, HATU (0.861 g, 2.26 mmol) dissolved in DMF was added, followed by *S*-methylbutanoic acid (255 µL, 2.34 mmol), DIPEA (795 µL, 4.68 mmol) and more DMF. The mixture was agitated for 1 h. The solvent was removed, and the resin was washed three times each with DMF, isopropanol and *n*-heptane. The cleavage cocktail, consisting of TFA/TIS/H_2_O (95:2.5:2.5), was added, coloring the mixture a dark violet. The resin was agitated for 30 min after which the supernatant was drained and the process was repeated once more. The combined filtrates were reduced under pressure and then dried further using lyophilization. Purification of 58% of the crude product using semi preparative HPLC (5-50-95% AcCN + 0.1% FA, NUCLEODUR® C18 Gravity SB, 3 µm, 250 x 10 mm, flow rate: 3 mL/min) yielded **4.4** as a colorless powder (54.5 mg, 0.0764 mmol, overall yield calculated to be: 17%).

**^1^H-NMR** (MeOD, 600 MHz): δ_H_ [ppm] = 7.28 (d, 4H, *J* = 4.6 Hz, C*H*_arom_ Phe), 7.24-7.20 (m, 1H, C*H*_arom_ Phe), 4.59 (t, 1H, *J* =7.9 Hz, α-C*H* Phe), 4.45 (dd, 1H, *J* = 9.4, 5.3 Hz, α-C*H* Leu), 4.34 (dd, 1H, *J* = 8.5, 5.7 Hz, α-C*H* Arg), 3.21 (t, 2H, *J* = 7.2 Hz, δ-*C*H_2_ Arg), 3.10 (dd, 1H, *J* = 13.7, 7.7 Hz, β-C*H*_2_ a Phe), 3.02 (dd, 1H, *J* = 13.7, 7.9 Hz, β-C*H*_2_ b Phe), 2.30-2.23 (m, 1H, C*H* *S*-MBA), 2.06-2.00 (m, 1H, β-C*H*_2_ a Arg), 2.07 (s, 3H, γ-C*H*_3_ a Dhv 1), 2.02 (s, 3H, γ-C*H*_3_ a Dhv 2), 1.90-1.82 (m, 1H, β-C*H*_2_ b Arg), 1.79 (s, 3H, γ-C*H*_3_ b Dhv 1), 1.77-1.69 (m, 3H, γ-C*H*_2_ Arg, γ-C*H* Leu), 1.69-1.60 (m, 2H, β-C*H*_2_ Leu), 1.60-1.52 (m, 1H, C*H_2_* a *S*-MBA), 1.47 (s, 3H, γ-C*H*_3_ b Dhv 2), 1.40-1.31 (m, 1H, C*H_2_* b *S*-MBA), 1.00 (d, 3H, *J* = 6.8 Hz, CH-C*H*_3_ *S*-MBA), 0.92 (d, 3H, *J* = 6.4 Hz, δ-C*H*_3_ a Leu), 0.91 (d, 3H, *J* = 6.4 Hz, δ-C*H*_3_ b Leu), 0.86 (t, 3H, *J* = 7.4 Hz, CH_2_-C*H*_3_ *S*-MBA).

**^13^C-NMR** (MeOD, 150 MHz): δ_C_ [ppm] = 179.9 (CO *S*-MBA), 176.9 (*C*OOH Leu), 173.44 (*C*O Arg), 173.38 (*C*O Phe), 168.0 (*C*O Dhv 1), 167.8 (*C*O Dhv 2), 158.7 (ζ-*C*_quart_ Arg), 141.5 (α-*C*_quart_ Dhv 1), 141.4 (α-*C*_quart_ Dhv 1), 138.0 (γ-*C*_quart_ Phe), 124.9 (β-*C*_quart_ Dhv 1), 124.6 (β-*C*_quart_ Dhv 2), 130.4, 129.6, 128.0 (*C*H_arom_ Phe), 56.7 (α-*C*H Phe), 54.9 (α-*C*H Arg), 52.9 (α-*C*H Leu), 43.1 (*C*H *S*-MBA), 42.11 (β-*C*H_2_ Leu), 42.06 (δ-*C*H_2_ Arg), 38.4 (β-*C*H_2_ Phe), 29.4 (β-*C*H_2_ Arg), 28.2 (*C*H_2_ *S*-MBA), 26.4 (γ-*C*H_2_ Arg), 25.9 (γ-*C*H Leu), 23.4 (δ-C*H*_3_ b Leu), 22.2 (δ-*C*H_3_ a Leu), 21.61 (γ-*C*H_3_ b Dhv 1), 21.57 (γ-*C*H_3_ b Dhv 2), 21.1 (γ-*C*H_3_ a Dhv 1), 20.9 (γ-*C*H_3_ a Dhv 2), 17.9 (CH-*C*H_3_ *S*-MBA), 12.3 (CH_2_-*C*H_3_ *S*-MBA).

Additional found signals: δ_H_ [ppm] = 8.16 (FA), 4.84 (H_2_O), 2.66 (DMSO), N*H* signals, COO*H* Leu and N*H*/N*H*_2_ Arg were not observed. δ_C_ [ppm] = 40.4 (DMSO).

Dhv 1 refers to the amino acid on the right side of *L*-leucine, and Dhv 2 to the amino acid on the right side of *L*-arginine. Differentiation is based on HMBC data.

**UPLC-HR-ESI-MS** *m/z* calcd for C_36_H_57_N_8_O_7_: 713.4345 [M+H]^+^; found: 713.4353 [M+H]^+^

## Screening of pure compounds

The minimum inhibitory concentrations (MIC) of pure compounds were determined as described by Orban et al.3 Pure compounds were screened against Escherichia coli ATCC35218, Mycobacterium smegmatis ATCC607, Staphylococcus aureus ATCC33592, Candida albicans FH2173 and Septoria tritici MUCL45407. Protease testings (human cysteine proteases cathepsin B and L, trypanosomal cysteine protease rhodesain) were performed at concentrations of 20 μM as described in Brinkmann et al.4.

## References

1. Wang, M. *et al.* Sharing and community curation of mass spectrometry data with Global Natural Products Social Molecular Networking. *Nat Biotechnol* **34**, 828–837 (2016).

2. Shannon, P. *et al.* Cytoscape: a software environment for integrated models of biomolecular interaction networks. *Genome Res* **13**, 2498–2504 (2003).

3. Orban, A. *et al.* 5’-Methoxyarmillane, a Bioactive Sesquiterpenoid Aryl Ester from the Fungus Armillaria ostoyae. *ChemBioChem* **25**, e202400168.

4. Brinkmann, S. *et al.* Identification, Characterization, and Synthesis of Natural Parasitic Cysteine Protease Inhibitors: Pentacitidins Are More Potent Falcitidin Analogues. *ACS Chem. Biol.* **17**, 576–589 (2022).

# Tables

## Table S1 Bacterial strains and sources

| Strains | Source |
| --- | --- |
| *Escherichia coli* WM3064 | W Metcalf (Unpublished) |
| *Pedobacter cryoconitis* DSM 14825^T^ | German Collection of Microorganisms and Cell Cultures (DSMZ, Germany) |
| *Pedobacter cryoconitis* PAMC 27485 | Polar and Alpine Microbial Collection (PAMC)- Korea Polar Research Institute (KOPRI) laboratory (South Korea) |
| *Pedobacter cryoconitis* PAMC 27485 ::∆*crpA* | This study |

## Table S2 Primers used in this study

| Primers | Primer sequence | Plasmid |
| --- | --- | --- |
| gyrBprom_f_pedtr | tttatcaaaacacgaaattcctttttttccaaaatgttaataaaaaataagattcaaaaataacc | pCRYO1 |
| gyrBprom_r_pedtr | cgaatcaggtattgttccttatgcgtgtattataatgttgtttttaattc | pCRYO1 |
| rpl3_r_pedtr | tatgccgtcttctgcttggcgcgcccttaatggcttaactgcgttttatc | pCRYO1 |
| rpl3_f_pedtr | tttactttgttcattttcttttttaaatttaacacccatcg | pCRYO1 |
| Erm_r_pedtr | ttaaatttaaaaaagaaaatgaacaaagtaaatataaaagatagtcaaaa | pCRYO1 |
| Erm_f_pedtr | ttataatacacgcataaggaacaatacctgattcggagac | pCRYO1 |
| ActivcassettePed_univ_r | aaatgttaataaaaaataagattcaaaaataaccaattatg | pCRYO2 |
| ActivcassettePed_univ_f | ttagccgttaaatattttataactattaaatagcgatac | pCRYO2 |
| ups_r_dMBL_crp | ttaatagttataaaatatttaacggctaagcaggataaaccttgcaaagg | pCRYO2 |
| ups_f_dMBL_crp | aggaacacttaacggctgacatgggctaaatggctgtcctctaaaagttc | pCRYO2 |
| dow_f_dMBL_crp | ttttgaatcttattttttattaacatttatgaaactctcatccaacacct | pCRYO2 |
| dow_r_dMBL_crp | ttaaatttaaaaaagaaaatgaacaaagtaaatataaaagatagtcaaaa | pCRYO2 |

## Table S3 Plasmids used and generated in this study

| Plasmids | Source |
| --- | --- |
| psamBT | Addgene ID112497 (Goodman et al., 2009) |
| pCRYO1 | This study |
| pCRYO2 | This study |

## Table S4. NMR data of cryopeptins 1 - 3 (DMSO-*d*_6_; *δ* in ppm, *J* in Hz; ^1^H at 700MHz, ^13^C at 176 MHz).

|  | **1** | | **2** | | **3** | |
| --- | --- | --- | --- | --- | --- | --- |
| position | *δ* ^13^C | *δ* ^1^H, multi. (*J*) | *δ* ^13^C | *δ* ^1^H, multi. (*J*) | *δ* ^13^C | *δ* ^1^H, multi. (*J*) |
| L-Leu |  |  |  |  |  |  |
| COOH | 176.00 |  | 175.92 |  | 175.79 |  |
| NH |  | 7.24, overlapping |  | 7.26, overlapping |  | 7.26, overlapping |
| α-CH | 52.77 | 3.87, dt (6.5, 7.1) | 52.68 | 3.89, dt (6.7, 3.9) | 52.47 | 3.92, dt (6.3, 7.4) |
| β-CH_2_ | 42.78 | 1.47, m  1.37, m | 42.63 | 1.46, m  1.39, m | 42.43 | 1.46, m  1.39, m |
| γ-CH | 24.53 | 1.65, m | 24.50 | 1.65, m | 24.46 | 1.65, m |
| δ-CH_3_ a | 22.73 | 0.86, d (6.6) | 22.94 | 0.85, d (6.6) | 22.95 | 0.86, d (6.6) |
| δ-CH_3_ b | 22.95 | 0.87, d (6.6) | 21.70 | 0.86, d (6.6) | 22.53 | 0.87, d (6.6) |
| Dhv 1 |  |  |  |  |  |  |
| CO | 164.22 |  | 164.36 |  | 164.43 |  |
| NH |  | 9.01, s |  | 8.97, s |  | 8.96, s |
| α-C_quart_ | 138.82 |  | 138.61 |  | 138.50 |  |
| β-C_quart_ | 124.46 |  | 124.50 |  | 124.51 |  |
| γ-CH_3_ a | 20.98 | 1.62, s | 20.86 | 1.61, s | 20.88 | 1.61, s |
| γ-CH_3_ b | 20.07 | 1.96, s | 20.60 | 1.95, s | 20.11 | 1.96, s |
| L-Arg |  |  |  |  |  |  |
| CO | 171.22 |  | 171.20 |  | 174.14 |  |
| NH |  | 7.62, d (6.7) |  | 7.71, d (6.2) |  | 7.82, d (6.8) |
| α-CH | 52.84 | 4.20, q (6.7) | 52.91 | 4.20, q (6.2) | 52.95 | 4.20, q (6.2) |
| β-CH_2_ | 28.91 | 1.93, overlapping  1.61, overlapping | 28.99 | 1.95, overlapping  1.62, overlapping | 28.99 | 1.95, overlapping  1.64, overlapping |
| γ-CH_2_ | 24.35 | 1.59, overlapping | 24.42 | 1.61, overlapping | 24.53 | 1.61, overlapping |
| δ-CH_2_ | 40.60 | 3.08, m  3.03, m | 40.62 | 3.07, m  3.03, m | 40.60 | 3.07, m  3.04, m |
| ε -NH |  | - |  |  |  | - |
| ζ - C_quart_ | 157.41 |  | 157.31 |  | 157.32 |  |
| η-NH |  | - |  |  |  | - |
| η-NH_2_ |  | - |  |  |  | - |
| Dhv2 |  |  |  |  |  |  |
| CO | 164.95 |  | 164.99 |  | 165.13 |  |
| NH |  | 9.26, s |  | 9.21, s |  | 9.21, s |
| α-C_quart_ | 137.00 |  | 136.85 |  | 136.28 |  |
| β-C_quart_ | 124.13 |  | 124.13 |  | 124.29 |  |
| γ-CH_3_ a | 20.89 | 1.45, s | 20.91 | 1.47, s | 20.84 | 1.50, s |
| γ-CH_3_ b | 20.25 | 1.94, s | 20.27 | 1.94, s | 20.30 | 1.93, s |
| L-Phe |  |  |  |  |  |  |
| CO | 170.82 |  | 170.74 |  | 170.74 |  |
| NH |  | 8.32, d (6.2) |  | 8.18, d (6.8) |  | 8.11, d (7.1) |
| α-CH | 54.68 | 4.46, dt (8.6, 6.2) | 54.57 | 4.46 dt (6.8, 9.1) | 54.33 | 4.48, dt (8.5, 6.7) |
| β-CH_2_ | 36.92 | 2.96 dd, (5.9, 13.8)  2.83, dd (9.1, 13.8) | 36.95 | 2.98, dd (5.9, 13.8)  2.85, dd (9.0, 13.8) | 37.03 | 2.99, dd (5.7, 13.8)  2.86, dd (9.4, 13.8) |
| γ-C_quart_ | 137.57 |  | 137.60 |  | 137.63 |  |
| CH_arom_ | 129.19  128.13  126.34 | 7.26, overlapping  7.26, overlapping  7.19, m | 129.17  128.09  126.31 | 7.26, overlapping  7.26, overlapping  7.19, m | 129.21  128.06  126.30 | 7.26, overlapping  7.26, overlapping  7.18, m |
| FA |  |  |  |  |  |  |
| 1 | 169.96 |  | 173.55 |  | 176.63 |  |
| 2 | 22.31 | 1.79, s | 28.09 | 2.08, ddd (3.3, 7.5, 15.0) | 33.52 | 2.39, dt (13.6, 6.8) |
| 2-Me |  |  | 9.59 | 0.9, t (6.6) | 19.47 | 0.85, d (6.8) |
| 3 |  |  |  |  | 19.12 | 0.92, d (6.8) |

## Table S5. NMR data of cryopeptins 4 - 5 (DMSO-*d*_6_; *δ* in ppm, *J* in Hz; ^1^H at 700MHz, ^13^C at 176 MHz).

|  | **4** | | **5** | |
| --- | --- | --- | --- | --- |
| position | *δ* ^13^C | *δ* ^1^H, multi. (*J*) | *δ* ^13^C | *δ* ^1^H, multi. (*J*) |
| L-Leu |  |  |  |  |
| COOH | 175.80 | - | 175.70 | - |
| NH |  | 7.27, overlapping | - | 7.26, overlapping |
| α-CH | 52.46 | 3.93, dt (6.2, 7.3) | 52.27 | 3.95, dt (6.2, 7.3) |
| β-CH_2_ | 42.42 | 1.45, m  1.40, m | 42.23 | 1.46, m  1.41, m |
| γ-CH | 24.45 | 1.65, m | 24.40 | 1.65, m |
| δ-CH_3_ a | 22.93 | 0.85, d (6.6) | 22.94 | 0.85, d (6.6) |
| δ-CH_3_ b | 22.51 | 0.87, d (6.6) | 22.43 | 0.86, d (6.6) |
| Dhv 1 |  |  |  |  |
| CO | 164.44 |  | 164.39 | - |
| NH |  | 8.96, s | - | 8.97, s |
| α-C_quart_ | 138.41 |  | 138.50 | - |
| β-C_quart_ | 124.52 |  | 124.46 | - |
| γ-CH_3_ a | 20.87 | 1.62, s | 20.95 | 1.62, s |
| γ-CH_3_ b | 20.11 | 1.95, s | 20.13 | 1.96, s |
| L-Arg |  |  |  |  |
| CO | 171.10 |  | 171.09 |  |
| NH |  | 7.85, d (6.6) | - | 7.82, d (6.4) |
| α-CH | 53.00 | 4.20, dt (6.6, 9.4) | 52.91 | 4.20, dt (6.4, 7.0) |
| β-CH_2_ | 29.02 | 1.94, overlapping  1.64, overlapping | 28.77 | 1.92, overlapping  1.63, overlapping |
| γ-CH_2_ | 24.53 | 1.61, overlapping | 22.56 | 1.60, overlapping |
| δ-CH_2_ | 40.63 | 3.07, m  3.04, m | 40.55 | 3.07, m  3.03, m |
| ε -NH |  | 9.60, s |  | - |
| ζ - C_quart_ | 157.30 |  | 157.30 |  |
| η-NH |  | - |  | - |
| η-NH_2_ |  | - |  | - |
| Dhv2 |  |  |  |  |
| CO | 165.09 |  | 165.14 |  |
| NH |  | 9.19, s | - | 9.26, s |
| α-C_quart_ | 136.68 |  | 136.42 |  |
| β-C_quart_ | 124.17 |  | 124.26 |  |
| γ-CH_3_ a | 20.91 | 1.48, s | 20.85 | 1.49, s |
| γ-CH_3_ b | 20.31 | 1.94, s | 20.30 | 1.94, s |
| L-Phe |  |  |  |  |
| CO | 170.65 |  | 170.85 |  |
| NH |  | 8.15, d (6.5) |  | 8.19, d (6.9) |
| α-CH | 54.35 | 4.50, dt (6.5, 9.4) | 54.45 | 4.52, dt (6.9, 9.5) |
| β-CH_2_ | 37.04 | 2.99, dd (6.5, 13.8)  2.87, dd (9.4, 13.8) | 36.99 | 2.98, dd (5.6, 13.8)  2.84, dd (9.4, 13.8) |
| γ-C_quart_ | 137.57 |  | 137.59 |  |
| CH_arom_ | 129.19  128.05  126.29 | 7.27, overlapping  7.27, overlapping  7.19, m | 129.16  128.10  126.29 | 7.26, overlapping  7.26, overlapping  7.18, m |
| FA |  |  |  |  |
| 1 | 179.06 |  | 172.14 |  |
| 2 | 40.79 | 2.19, q (6.9) | 44.27 | 1.94, overlapping  1.96, overlapping |
| 2-Me | 17.31 | 0.83, d (6.9) | - | - |
| 3 | 26.66 | 1.43, m  1.22, dt (6.9, 13.7) | 25.44 | 1.85, m |
| 4 | 11.68 | 0.75, t (7.8) | 22.22 | 0.77, d (6.6) |
| 5 |  |  | 22.12 | 0.72, d (6.6) |

## Table S6. NMR data of cryopeptins 6 - 8 (DMSO-*d*_6_; *δ* in ppm, *J* in Hz; ^1^H at 700MHz, ^13^C at 176 MHz).

|  | **6** | | **7** | | **8** | |
| --- | --- | --- | --- | --- | --- | --- |
| position | *δ* ^13^C | *δ* ^1^H, multi. (*J*) | *δ* ^13^C | *δ* ^1^H, multi. (*J*) | *δ* ^13^C | *δ* ^1^H, multi. (*J*) |
| L-Leu |  |  |  |  |  |  |
| COOH | 175.84 |  | 175.77 |  | 176.05 |  |
| NH |  | 7.27, overlapping |  | 7.28, overlapping |  | 7.26, overlapping |
| α-CH | 52.43 | 3.91, dt (5.7, 7.7) | 52.34 | 3.93, dt (6.3, 6.8) | 52.70 | 3.85, dt (6.0, 7.1) |
| β-CH_2_ | 42.43 | 1.45, m  1.38, m | 42.32 | 1.46, m  1.42, m | 42.83 | 1.45, m  1.36, m |
| γ-CH | 24.47 | 1.65, m | 24.44 | 1.66, m | 24.57 | 1.66, m |
| δ-CH_3_ a | 22.97 | 0.85, d (6.6) | 22.9 | 0.86, d (6.6) | 22.98 | 0.86, d (6.6) |
| δ-CH_3_ b | 22.55 | 0.86, d (6.6) | 22.50 | 0.87, d (6.6) | 22.75 | 0.88, d (6.6) |
| Dhv 1 |  |  |  |  |  |  |
| CO | 164.36 |  | 164.34 |  | 164.29 |  |
| NH |  | 8.99, s |  | 9.00, s |  | 9.01, s |
| α-C_quart_ | 138.72 |  | 138.74 |  | 138.81 |  |
| β-C_quart_ | 124.46 |  | 124.42 |  | 124.51 |  |
| γ-CH_3_ a | 21.01 | 1.61, s | 21.03 | 1.63, s | 20.97 | 1.61, s |
| γ-CH_3_ b | 20.14 | 1.96, s | 20.15 | 1.98, s | 20.29 | 1.96, s |
| L-Arg |  |  |  |  |  |  |
| CO | 171.17 |  | 171.12 |  | 171.24 |  |
| NH |  | 7.78, d (6.7) |  | 7.78, d (6.8) |  | 7.71, d (6.7) |
| α-CH | 52.87 | 4.20, dt (6.5, 7.0) | 52.83 | 4.21, dt (5.3. 6.4) | 52.70 | 4.21, dt (5.5. 6.4) |
| β-CH_2_ | 28.81 | 1.93, overlapping  1.63, overlapping | 28.73 | 1.93, overlapping  1.64, overlapping | 28.94 | 1.94, overlapping  1.63, overlapping |
| γ-CH_2_ | 24.29 | 1.59, overlapping | 24.49 | 1.61, overlapping | 24.29 | 1.60, overlapping |
| δ-CH_2_ | 40.69 | 3.08, m  3.03, m | 40.58 | 3.09, m  3.05, m | 40.61 | 3.08, m  3,02, m |
| ε -NH |  | - |  | - |  | - |
| ζ - C_quart_ | 157.30 |  | 157.25 |  | 157.36 |  |
| η-NH |  | - |  | - |  | - |
| η-NH_2_ |  | - |  | - |  | - |
| Dhv2 |  |  |  |  |  |  |
| CO | 165.12 |  | 165.13 |  | 165.01 |  |
| NH |  | 9.27, s |  | 9.28, s |  | 9.22, s |
| α-C_quart_ | 136.32 |  | 136.25 |  | 136.47 |  |
| β-C_quart_ | 124.31 |  | 124.33 |  | 124.28 |  |
| γ-CH_3_ a | 20.86 | 1.49, s | 20.85 | 1.50, s | 20.88 | 1.48, s |
| γ-CH_3_ b | 20.31 | 1.93, s | 20.30 | 1.94, s | 20.27 | 1.93, s |
| L-Phe |  |  |  |  |  |  |
| CO | 170.89 |  | 170.92 |  | 170.80 |  |
| NH |  | 8.20, d (7.0) |  | 8.21 d (6.7) |  | 8.21, d (6.8) |
| α-CH | 54.45 | 4.49, dt (8.4, 7.0) | 54.42 | 4.51, dt (8.0, 6.7) | 54.45 | 4.47, dt (8.2, 6.8) |
| β-CH_2_ | 37.02 | 2.98, dd (5.6, 13.8)  2.83, dd (9.4, 13.8) | 36.99 | 2.99, dd (5.4, 13.8)  2.84, dd (9.2, 13.8) | 36.97 | 2.97, dd (6.8, 13.8)  2.82, dd (9.1, 13.8) |
| γ-C_quart_ | 137.65 |  | 137.64 |  | 137.69 |  |
| CH_arom_ | 129.20  128.11  126.33 | 7.27, overlapping  7.25, overlapping  7.18, m | 129.19  128.10  126.22 | 7.27, overlapping  7.27, overlapping  7.20, m | 129.18  128.10  126.31 | 7.26, overlapping  7.26, overlapping  7.19, m |
| FA |  |  |  |  |  |  |
| 1 | 172.82 |  | 172.82 |  | 173.03 |  |
| 2 | 34.98 | 2.05, m | 35.26 | 2.04, m | 32.80 | 2.09, m  2.01, m |
| 3 | 24.78 | 1.38, m | 22.96 | 1.40, m | 31.76, | 1.40, m  1.20, m |
| 4 | 30.69 | 1.09, m | 37.79 | 1.01, m | 33.33, | 1.20, m |
| 5 | 21.84 | 1.19, m | 27.19 | 1.45, m | 28.55 | 1.23, m  1.04, m |
| 6 | 13.85 | 0.80, t (7.3) | 22.42 | 0.80, d (6.6) | 11.10, | 0.78, t (7.2) |
| 7 |  |  | 22.39 | 0.80, d (6.6) | 18.79, | 0.76, d (6.3) |

## Table S7. NMR data of cryopeptins 9 - 10 (DMSO-*d*_6_; *δ* in ppm, *J* in Hz; ^1^H at 700MHz, ^13^C at 176 MHz).

|  | **9** | | **10** | |
| --- | --- | --- | --- | --- |
| position | *δ* ^13^C | *δ* ^1^H, multi. (*J*) | *δ* ^13^C | *δ* ^1^H, multi. (*J*) |
| L-Leu |  |  |  |  |
| COOH | 176.08 |  | 175.96 |  |
| NH |  | 7.26, overlapping |  | 7.26, overlapping |
| α-CH | 52.88 | 3.83, dt (5.9, 7.3) | 52.76 | 3.86, dt (6.5, 7,0) |
| β-CH_2_ | 42.93 | 1.46, m  1.37, m | 42.78 | 1.41, m  1.37, m |
| γ-CH | 24.57 | 1.67, m | 24.53 | 1.66, m |
| δ-CH_3_ a | 22.94 | 0.86, d (6.6) | 22.94 | 0.86, d (6.6) |
| δ-CH_3_ b | 22.79 | 0.87, d (6.6) | 22.72 | 0.88, d (6.6) |
| Dhv 1 |  |  |  |  |
| CO | 164.23 |  | 164.24 |  |
| NH |  | 9.00, s |  | 9.00, s |
| α-C_quart_ | 138.70 |  | 138.67 |  |
| β-C_quart_ | 124.51 |  | 124.50 |  |
| γ-CH_3_ a | 20.90 | 1.61, s | 20.93 | 1.61, s |
| γ-CH_3_ b | 20.03 | 1.95, s | 20.05 | 1.96, s |
| L-Arg |  |  |  |  |
| CO | 171.19 |  | 171.16 |  |
| NH |  | 7.65, d (6.8) |  | 7.68, d (6.8) |
| α-CH | 52.75 | 4.22, dt (6.0, 6.5) | 52.76 | 4.22, dt (6.0, 6.4) |
| β-CH_2_ | 29.03 | 1.95, overlapping  1.63, overlapping | 28.95 | 1.93, overlapping  1.61, overlapping |
| γ-CH_2_ | 24.21 | 1.61, overlapping | 24.29 | 1.61, overlapping |
| δ-CH_2_ | 40.59 | 3.08, m  3.03, m | 40.55 | 3.07, m  3.03, m |
| ε -NH |  | - |  | - |
| ζ - C_quart_ | 157.35 |  | 157.40 |  |
| η-NH |  | - |  | - |
| η-NH_2_ |  | - |  | - |
| Dhv2 |  |  |  |  |
| CO | 164.92 |  | 164.97 |  |
| NH |  | 9.19, s |  | 9.22, s |
| α-C_quart_ | 136.57 |  | 136.50 |  |
| β-C_quart_ | 124.22 |  | 124.25 |  |
| γ-CH_3_ a | 20.84 | 1.49, s | 20.84 | 1.49, s |
| γ-CH_3_ b | 20.22 | 1.93, s | 20.03 | 1.93, s |
| L-Phe |  |  |  |  |
| CO | 170.72 |  | 170.78 |  |
| NH |  | 8.16, d (7.2) |  | 8.19, d (7.0) |
| α-CH | 54.38 | 4.48, dt (6.9, 9.4) | 54.41 | 4.49, dt (7.0, 8.0) |
| β-CH_2_ | 36.93 | 2.98, dd (5.6, 13.8)  2.83, dd (9.4, 13.8) | 36.94 | 2.98, dd (5.3, 13.7)  2.83, dd (9.7, 13.7) |
| γ-C_quart_ | 137.66 |  | 137.67 |  |
| CH_arom_ | 129.12  128.03  126.24 | 7.26, overlapping  7.26, overlapping  7.18, m | 129.12  128.04  126.25 | 7.26, overlapping  7.26, overlapping  7.18, m |
| FA |  |  |  |  |
| 1 | 172.72 |  | 172.77 |  |
| 2 | 35.00 | 2.05, m | 35.04 | 2.06, m |
| 3 | 25.06 | 1.38, m | 25.30 | 1.37, m |
| 4 | 28.36 | 1.11, m | 26.23 | 1.12, m |
| 5 | 28.38 | 1.18, m | 38.11 | 1.07, m |
| 6 | 31.11 | 1.18, m | 27.22 | 1.45, m |
| 7 | 21.99 | 1.23, m | 22.46 | 0.81, d (6.6) |
| 8 | 13.90 | 0.85, t (7.3) | 22.42 | 0.81, (6.6) |

## Table S8. NMR data of cryopeptins 11-12 (DMSO-*d*_6_; *δ* in ppm, *J* in Hz; ^1^H at 700MHz, ^13^C at 176 MHz).

|  | **11** | | **12** | |
| --- | --- | --- | --- | --- |
| position | δ ^13^C | δ ^1^H, multi. (*J*) | δ ^13^C | δ ^1^H, multi. (*J*) |
| L-Leu |  |  |  |  |
| COOH | 176.27 |  | 176.30 |  |
| NH |  | 7.13, d (7.4) |  | 7.14, d (7.3) |
| α-CH | 52.63 | 3.93, dt (6.3, 7.0) | 52.76 | 3.92, dt (6.0, 6.9) |
| β-CH_2_ | 42.82 | 1.43, m  1.36, m | 42.84 | 1.45, m  1.35, m |
| γ-CH | 24.38 | 1.62, overlapping | 24.40 | 1.64, overlapping |
| δ-CH_3_ a | 22.99 | 0.83, d (6.6) | 23.00 | 0.83, d (6.6) |
| δ-CH_3_ b | 22.63 | 0.85, d (6.6) | 22.65 | 0.85, d (6.6) |
| Dhv 1 |  |  |  |  |
| CO | 164.13 |  | 164.14 |  |
| NH |  | 8.93, s |  | 8.94, s |
| α-C_quart_ | 138.79 |  | 138.77 |  |
| β-C_quart_ | 124.38 |  | 124.41 |  |
| γ-CH_3_ a | 20.95 | 1.62, s | 20.95 | 1.62, s |
| γ-CH_3_ b | 20.28 | 1.95, s | 20.12 | 1.95, s |
| L-Arg 1 |  |  |  |  |
| CO | 171.55 |  | 171.20 |  |
| NH |  | 7.68, br. s |  | 7.63, br. s |
| α-CH | 52.93 | 4.16, d (6.4) | 52.95 | 4.16, d (6.9) |
| β-CH_2_ | 28.80 | 1.90, overlapping  1.61, overlapping | 28.84 | 1.90, overlapping  1.61, overlapping |
| γ-CH_2_ | 24.66 | 1.58, overlapping | 24.65 | 1.59, overlapping |
| δ-CH_2_ | 40.22 | 3.05. m | 40.22 | 3.05, m |
| ε -NH |  | - |  | - |
| ζ - C_quart_ | 157.36 |  | 157.39 |  |
| η-NH |  | - |  | - |
| η-NH_2_ |  | - |  | - |
| Dhv 2 |  |  |  |  |
| CO | 164.83 |  | 164.80 |  |
| NH |  | 9.13, s |  | 9.12, s |
| α-C_quart_ | 136.63 |  | 136.74 |  |
| β-C_quart_ | 124.26 |  | 124.22 |  |
| γ-CH_3_ a | 20.98 | 1.71, s | 20.98 | 1.70, s |
| γ-CH_3_ b | 20.12 | 1.97, s | 20.29 | 1.97, s |
| L-Arg 2 |  |  |  |  |
| CO | 171.56 |  | 171.58 |  |
| NH |  | 8.30, br. s |  | 8.27, br. s |
| α-CH | 53.25 | 4.25, br. s | 53.28 | 4.24, br. s |
| β-CH_2_ | 27.83 | 1.89, overlapping  1.69, overlapping | 27.80 | 1.89, overlapping  1.69, overlapping |
| γ-CH_2_ | 25.05 | 1.53, overlapping | 25.03 | 1.53, overlapping |
| δ-CH_2_ | 40.54 | 3.05, m | 40.56 | 3.05, m |
| ε -NH |  | - |  | - |
| ζ - C_quart_ | 157.28 |  | 157.31 |  |
| η-NH |  | - |  | - |
| η-NH_2_ |  | - |  | - |
| Dhv3 |  |  |  |  |
| CO | 166.01 |  | 166.14 |  |
| NH |  | 9.52, s |  | 9.56, s |
| α-C_quart_ | 135.12 |  | 135.03 |  |
| β-C_quart_ | 124.55 |  | 124.58 |  |
| γ-CH_3_ a | 20.52 | 1.56, s | 20.50 | 1.55, s |
| γ-CH_3_ b | 20.26 | 1.90, s | 20.26 | 1.90, s |
| L-Phe |  |  |  |  |
| CO | 171.22 |  | 171.34 |  |
| NH |  | 8.16, br. s |  | 8.18, br. s |
| α-CH | 54.11 | 4.56, br. s | 54.28 | 4.56, br. s |
| β-CH_2_ | 37.20 | 3.00, dd (4.7, 13.8)  2.86, dd (8.6, 13.8) | 37.14 | 2.99, dd (5.1, 13.8)  2.83, dd (9.2, 13.8) |
| γ-C_quart_ | 137.63 |  | 137.61 |  |
| CH_arom_ | 129.16  128.00  126.24 | 7.26, overlapping  7.26, overlapping  7.18, m | 129.12  128.05  126.26 | 7.26, overlapping  7.26, overlapping  7.18, m |
| FA |  |  |  |  |
| 1 | 175.93 |  | 171.98 |  |
| 2 | 40.92 | 2.16, m | 44.29 | 1.93, overlapping  1.90, overlapping |
| 3 | 26.42 | 1.42, m  1.20, m | 25.36 | 1.83, m |
| 4 | 11.65 | 0.73, t (7.4) | 22.23 | 0.76, d (6.5) |
| 5 | 17.29 | 0.81, d (6.8) | 22.06 | 0.70. d (6.5) |
| 6 |  |  |  |  |
| 7 |  |  |  |  |

## Table S9. NMR data of cryopeptins 13 - 14 (DMSO-*d*_6_; *δ* in ppm, *J* in Hz; ^1^H at 700MHz, ^13^C at 176 MHz).

|  | **13** | | **14** | |
| --- | --- | --- | --- | --- |
| position | δ ^13^C | δ ^1^H, multi. (*J*) | δ ^13^C | δ ^1^H, multi.(*J*) |
| L-Leu |  |  |  |  |
| COOH | 176.26 |  | 176.24 |  |
| NH |  | 7.14, d (7.1) |  | 7.15, d (7.6) |
| α-CH | 52.61 | 3.93, d (7.0) | 52.60 | 3.93, dt (6.1, 7.3) |
| β-CH_2_ | 42.72 | 1.45, m  1.36, m | 42.71 | 1.44, m  1.36, m |
| γ-CH | 24.38 | 1.64, overlapping | 24.38 | 1.64, overlapping |
| δ-CH_3_ a | 22.99 | 0.83, d (6.6) | 22.99 | 0.83, d (6.6) |
| δ-CH_3_ b | 22.60 | 0.85, d (6.6) | 22.60 | 0.85, d (6.6) |
| Dhv 1 |  |  |  |  |
| CO | 164.18 |  | 164.17 |  |
| NH |  | 8.93, s |  | 8.93, s |
| α-C_quart_ | 138.77 |  | 138.76 |  |
| β-C_quart_ | 124.40 |  | 124.39 |  |
| γ-CH_3_ a | 20.99 | 1.62, s | 20.96 | 1.62, s |
| γ-CH_3_ b | 20.14 | 1.96, s | 20.14 | 1.96, s |
| L-Arg 1 |  |  |  |  |
| CO | 171.19 |  | 171.18 |  |
| NH |  | 7.63, br. s |  | 7.63, br. s |
| α-CH | 52.95 | 4.16, br. s | 52.93 | 4.16, br. s |
| β-CH_2_ | 28.80 | 1.90, overlapping  1.62, overlapping | 28.81 | 1.90, overlapping  1.62, overlapping |
| γ-CH_2_ | 24.70 | 1.58, overlapping | 24.68 | 1.58, overlapping |
| δ-CH_2_ | 40.55 | 3.05, m | 40.54 | 3.05, m |
| ε -NH |  | - |  | - |
| ζ - C_quart_ | 157.39 |  | 157.38 |  |
| η-NH |  | - |  | - |
| η-NH_2_ |  | - |  | - |
| Dhv 2 |  |  |  |  |
| CO | 164.81 |  | 164.80 |  |
| NH |  | 9.12, s |  | 9.12, s |
| α-C_quart_ | 136.84 |  | 136.79 |  |
| β-C_quart_ | 124.20 |  | 124.19 |  |
| γ-CH_3_ a | 20.99 | 1.70, s | 20.98 | 1.70, s |
| γ-CH_3_ b | 20.26 | 1.97, s | 20.25 | 1.97, s |
| L-Arg 2 |  |  |  |  |
| CO | 171.61 |  | 171.60 |  |
| NH |  | 8.28, br. s |  | 8.27, br. s |
| α-CH | 53.33 | 4.23, br. s | 53.32 | 4.23, br. s |
| β-CH_2_ | 27.79 | 1.89, overlapping  1.69, overlapping | 27.79 | 1.88, overlapping  1.68, overlapping |
| γ-CH_2_ | 24.70 | 1.53, overlapping | 25.10 | 1.52, overlapping |
| δ-CH_2_ | 40.22 | 3.05, m | 40.21 | 3.05, m |
| ε -NH |  | - |  | - |
| ζ - C_quart_ | 157.32 |  | 157.30 |  |
| η-NH |  | - |  | - |
| η-NH_2_ |  | - |  | - |
| Dhv3 |  |  |  |  |
| CO | 166.12 |  | 166.11 |  |
| NH |  | 9.58, s |  | 9.58, s |
| α-C_quart_ | 134.95 |  | 134.90 |  |
| β-C_quart_ | 124.60 |  | 124.60 |  |
| γ-CH_3_ a | 20.48 | 1.55, s | 20.48 | 1.55, s |
| γ-CH_3_ b | 20.96 | 1.90, s | 20.27 | 1.90, s |
| L-Phe |  |  |  |  |
| CO | 171.43 |  | 171.47 |  |
| NH |  | 8.19, br. s |  | 8.20, br. s |
| α-CH | 54.31 | 4.54, br. s | 54.29 | 4.53, br. s |
| β-CH_2_ | 31.76 | 2.99, dd (4.7, 13.8)  2.83, dd (8.5, 13.8) | 37.14 | 2.99  2.83 |
| γ-C_quart_ | 137.61 |  | 137.55 |  |
| CH_arom_ | 129.13  128.06  126.18 | 7.26, overlapping  7.26, overlapping  7.18, m | 129.15  128.06  126.27 | 7.26, overlapping  7.26, overlapping  7.18, m |
| FA |  |  |  |  |
| 1 | 172.71 |  | 172.69 |  |
| 2 | 34.99 | 2.03, m | 35.25 | 2.01, m |
| 3 | 24.70 | 1.37, | 22.90 | 1.36, m |
| 4 | 30.70 | 1.08, m | 37.81 | 0.99, m |
| 5 | 21.79 | 1.19, m | 27.17 | 1.44, m |
| 6 | 13.78 | 0.80, t (7.3) | 22.32 | 0.79, d (6.6) |
| 7 |  |  | 22.38 | 0.79, d (6.6) |

## Table S10. NMR data comparison in MeOD for synthetic (4.4) and natural, isolated compound 4.

NMR data comparison for **4.4** in MeOD for synthetic one (600 MHz, 101 MHz) and natural, isolated compound **4** (600 MHz, 151 MHz). The data of the natural, isolated compound **4** is written in blue.

| **Position** | **δ ^13^C [ppm]** | | **δ ^1^H [ppm]; (m, ∫, *J*)** | |
| --- | --- | --- | --- | --- |
|  | synthetic | **4** | Synthetic | **4** |
| l-Leu | | | | |
| **COOH** | 176.9 | 176.2 | n.o. | - |
| **NH** | - | - | n.o. | - |
| **α-CH** | 52.9 | 52.3 | 4.45 (dd, 1H, *J* = 9.4, 5.3 Hz) | 4.48 (dd, *J* = 9.8, 5.0 Hz ) |
| **β-CH_2_** | 42.11 | 41.7 | 1.69-1.60 (m, 2H) | 1.68 (m), 1.63 (m) |
| **γ-CH** | 25.9 | 25.8 | 1.77-1.69 (m, 3H, overlay) | 1.73 (m) |
| **δ-CH_3_ a** | 22.2 | 22.0 | 0.92 (d, 3H, *J* = 6.4 Hz) | 0.90 (d, *J* = 6.4 Hz) |
| **δ-CH_3_ b** | 23.4 | 23.4 | 0.91 (d, 3H, *J* = 6.4 Hz) | 0.90 (d, *J* = 6.4 Hz) |
| Dhv 1 | | | | |
| **CO** | 168.0 | 167.9 | - | - |
| **NH** | - | - | n.o. | - |
| **α-C_quart_** | 141.5 | 141.3 | - | - |
| **β-C_quart_** | 124.9 | 124.9 | - | - |
| **γ-CH_3_ a** | 21.1 | 20.9 | 2.07 (s, 3H) | 2.08 (s) |
| **γ-CH_3_ b** | 21.61 | 21.6 | 1.79 (s, 3H) | 1.80 (s) |
| l-Arg | | | | |
| **CO** | 173.44 | 173.4 | - | - |
| **NH** | - | - | n.o. | - |
| **α-CH** | 54.9 | 54.9 | 4.34 (dd, 1H, *J* = 8.5, 5.7 Hz) | 4.30 (dd, *J* = 8.7, 5.4 Hz) |
| **β-CH_2_** | 29.4 | 29.2 | 2.06-2.00 (m, 1H)  1.90-1.82 (m, 1H) | 2.04 (m)  1.86 (m) |
| **γ-CH_2_** | 26.4 | 26.5 | 1.77-1.69 (m, 3H, overlay) | 1.71 (m), 1.69 (m) |
| **δ-CH_2_** | 42.06 | 42.0 | 3.21 (t, 2H, *J* = 7.2 Hz) | 3.21 (t, *J* = 7.1 Hz) |
| **ε-NH** | - | - | n.o. | - |
| **ζ-*C*_quart_** | 158.7 | 158.6 | - | - |
| **η-NH** | - | - | n.o. | - |
| **η-NH_2_** | - | - | n.o. | - |
| Dhv 2 | | | | |
| **CO** | 167.8 | 168.0 | - | - |
| **NH** | - | - | n.o. | - |
| **α-C_quart_** | 141.4 | 141.7 | - | - |
| **β-C_quart_** | 124.6 | 124.5 | - | - |
| **γ-CH_3_ a** | 20.9 | 21.1 | 2.02 (s, 3H) | 2.02 (s) |
| **γ-CH_3_ b** | 21.57 | 21.7 | 1.47 (s, 3H) | 1.45 (s) |
| l-Phe | | | | |
| **CO** | 173.38 | 173.4 | - | - |
| **NH** | - | - | n.o. | - |
| **α-CH** | 56.7 | 56.8 | 4.59 (t, 1H, *J* =7.9 Hz) | 4.58 (t, *J* =7.9 Hz) |
| **β-CH_2_** | 38.4 | 38.4 | 3.10 (dd, 1H, *J* = 13.7, 7.7 Hz)  3.02 (dd, 1H, *J* = 13.7, 7.9 Hz) | 3.09 (dd, 1H, *J* = 13.7, 7.8 Hz)  3.01 (dd, 1H, *J* = 13.7, 7.8 Hz) |
| **γ-C_quart_** | 138.0 | 137.9 | - | - |
| **CH_arom_** | 130.4, 129.6, 128.0 | 130.4, 129.7, 128.0 | 7.28 (d, 4H, *J* = 4.6 Hz)  7.24-7.20 (m, 1H) | 7.29 (overlay)  7.22 (m) |
| SMB | | | | |
| **CO** | 179.9 | 180.0 | - | - |
| **CH** | 43.1 | 43.1 | 2.30-2.23 (m, 1H) | 2.27 (m) |
| **CH_3_** | 17.9 | 17.9 | 1.00 (d, 3H, *J* = 6.8 Hz) | 1.0 (d, *J* = 6.9 Hz) |
| **CH_2_** | 28.2 | 28.2 | 1.60-1.52 (m, 1H)  1.40-1.31 (m, 1H) | 1.56 (m)  1.36 (m) |
| **CH_3_** | 12.3 | 12.4 | 0.86 (t, 3H, *J* = 7.4 Hz) | 0.86 (t, *J* = 7.4 Hz) |

## Table S11. Bioactivity screening. Minimum Inhibitory Concentration (MIC) of 4 and 5

| **MIC**  **(µg/mL)** | ***Escherichia coli*** | ***Myobacterium smegmatis*** | ***Staphylococcus aureus*** | ***Candida  albicans*** | ***Septoria tritici*** |
| --- | --- | --- | --- | --- | --- |
|  | **ATCC 35218** | **ATCC607** | **ATCC 33592** | **FH2173** | **MUCL45407** |
| cryopeptin D (**4**) | >128 | >128 | >128 | >128 | >128 |
| cryopeptin E (**5**) | >128 | >128 | >128 | >128 | >128 |

# Figures

| **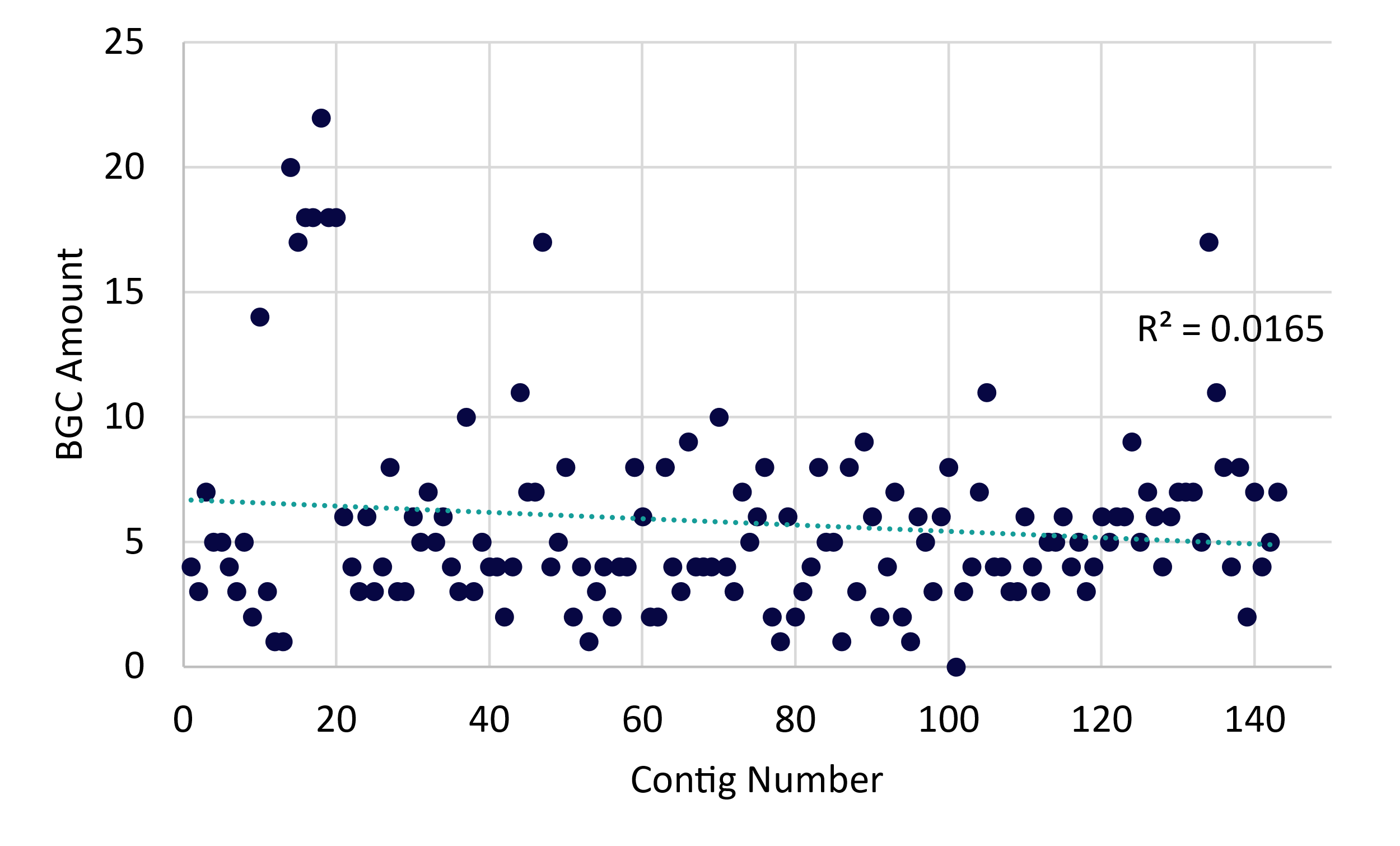** |
| --- |

## Figure S1-1. Plot showing the number of antiSMASH detected BGCs plotted against the number of contigs per respective genome.

| 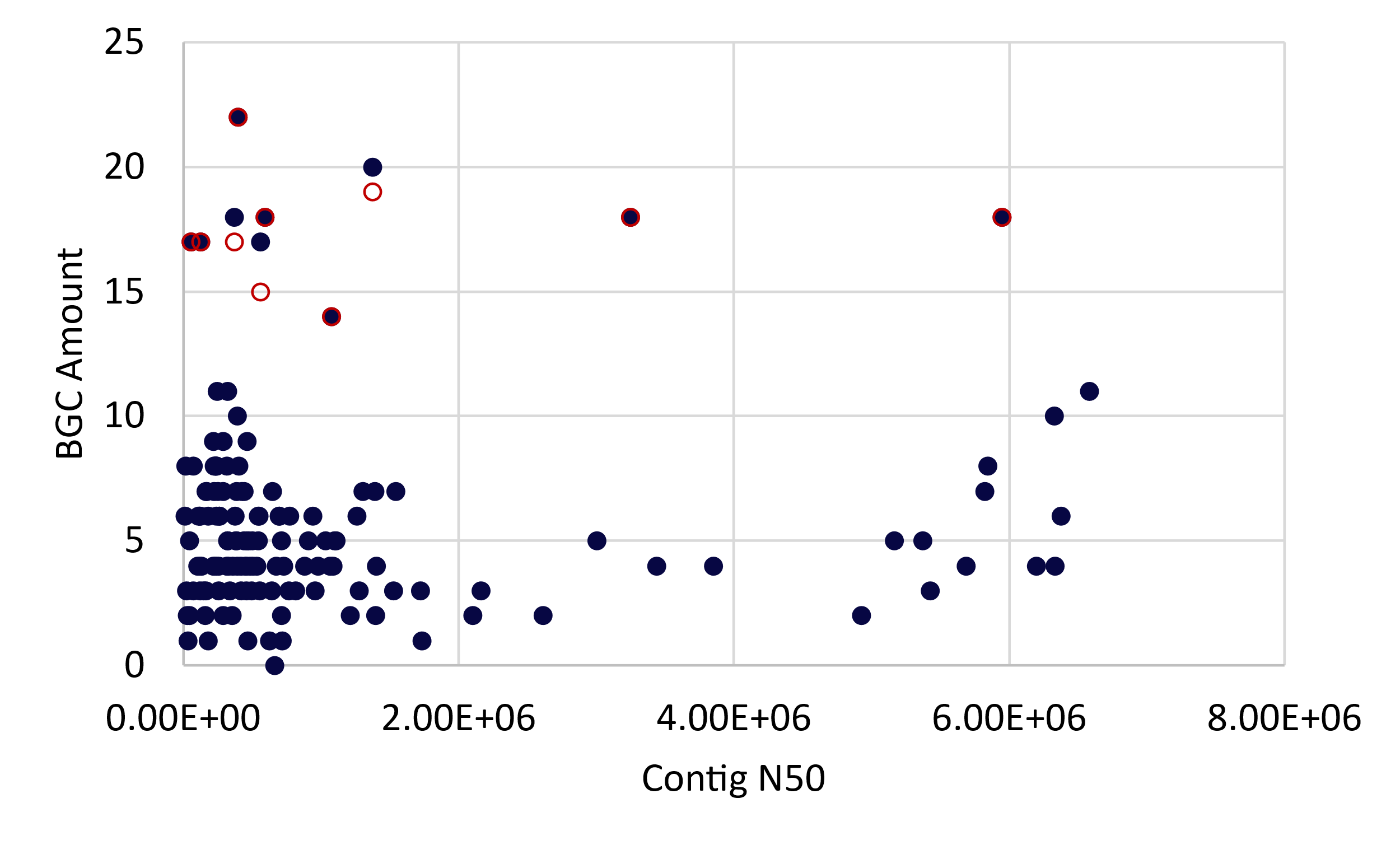 |
| --- |

## Figure S1-2. Plot showing the N50 values plotted against the BGC amount. Low N50 genomes containing many BGCs were manually curated to remove broken cluster fragments. Three strains contained broken NRPS clusters, resulting in a lower final BGC count (red circles).

| **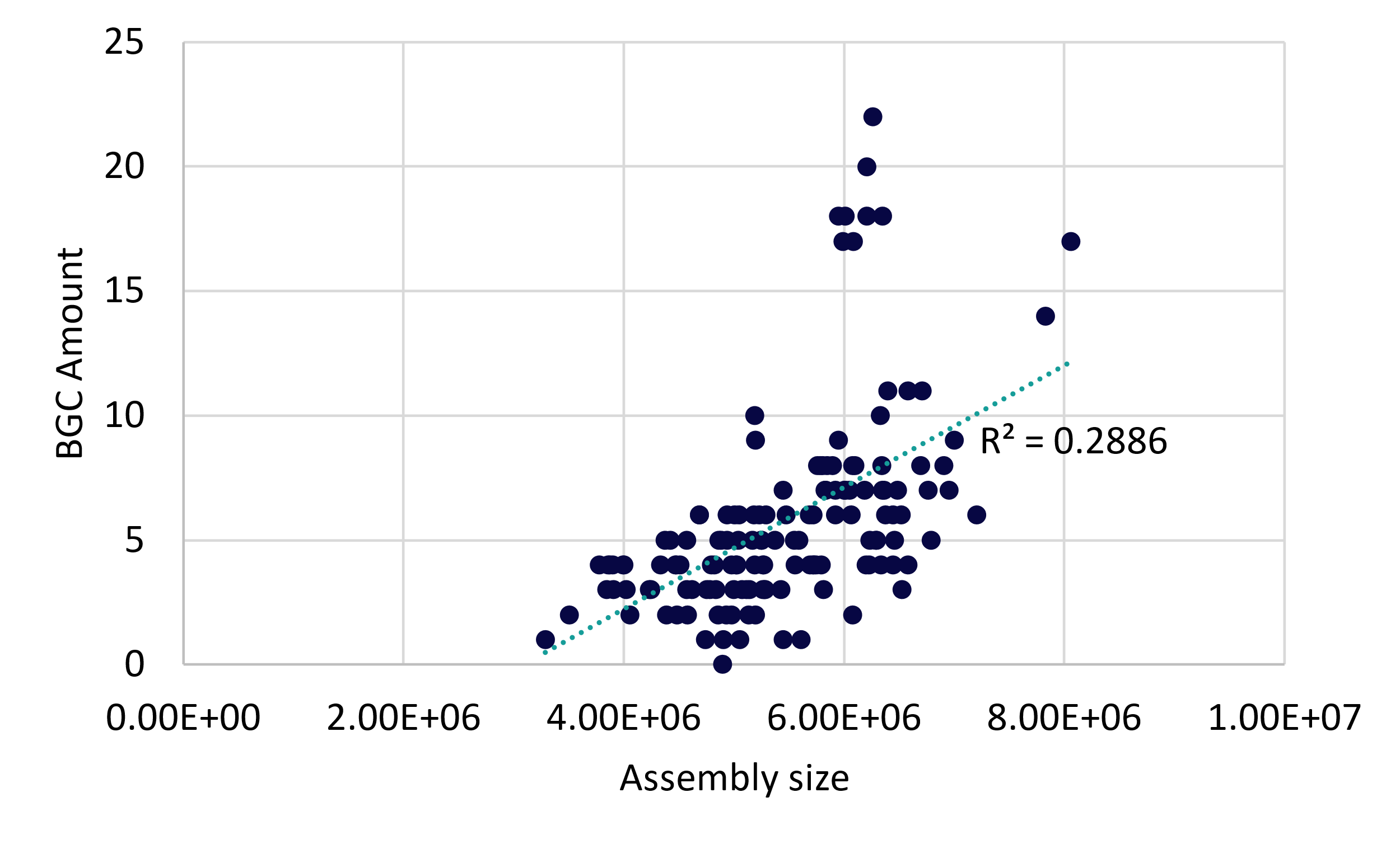** |
| --- |

## Figure S1-3. Plot showing the relationship between the number of detected BGCs and the total assembly size of each respective genome.

| 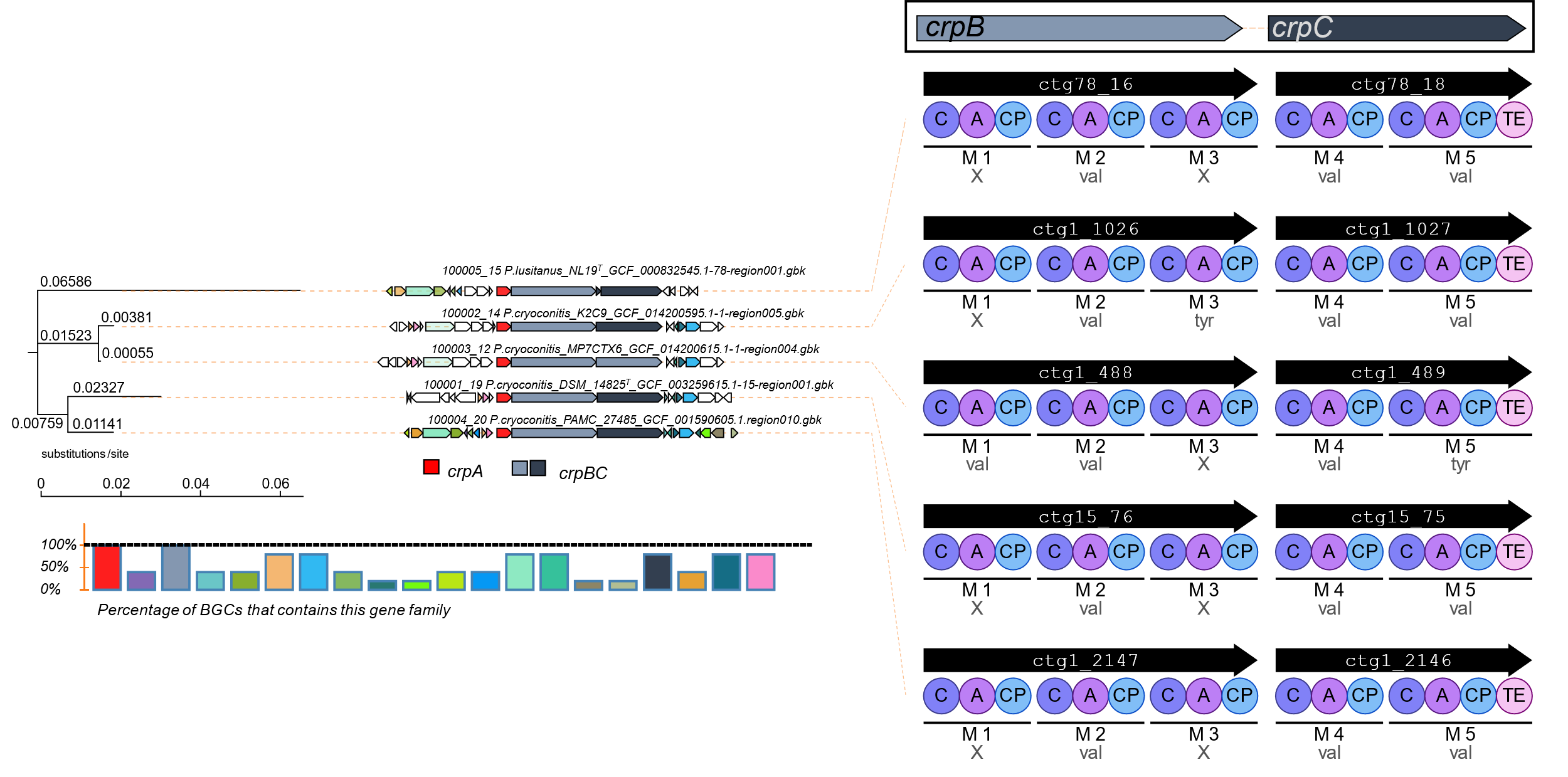 |
| --- |

## Figure S1-4. Corason Alignment of BiG-SCAPE identified GCF NRPS - 4 Cryopeptin. Corason was run in default settings, with *crpA* (red) selected as the query gene. Homologous genes predicted by Corason have the same color. The lower left panel of the Corason output indicates the presence of the individual genes of the Cryopeptin BGC in the different genomes (100% - present in all strains). The right side shows the antiSMASH predicted a-domain specificities of the different Cryopeptin BGCs. The *crpC* gene of *P. cryoconitis* MP7CTX6 has a predicted difference (indicated by the color (=*crpB)*) in a-domain specificity (tyr instead of val) but shares the same number of modules (n=2; M4, M5) and domain classes (C4: LCL and C5: modAA as predicted by NaPDoS).

| 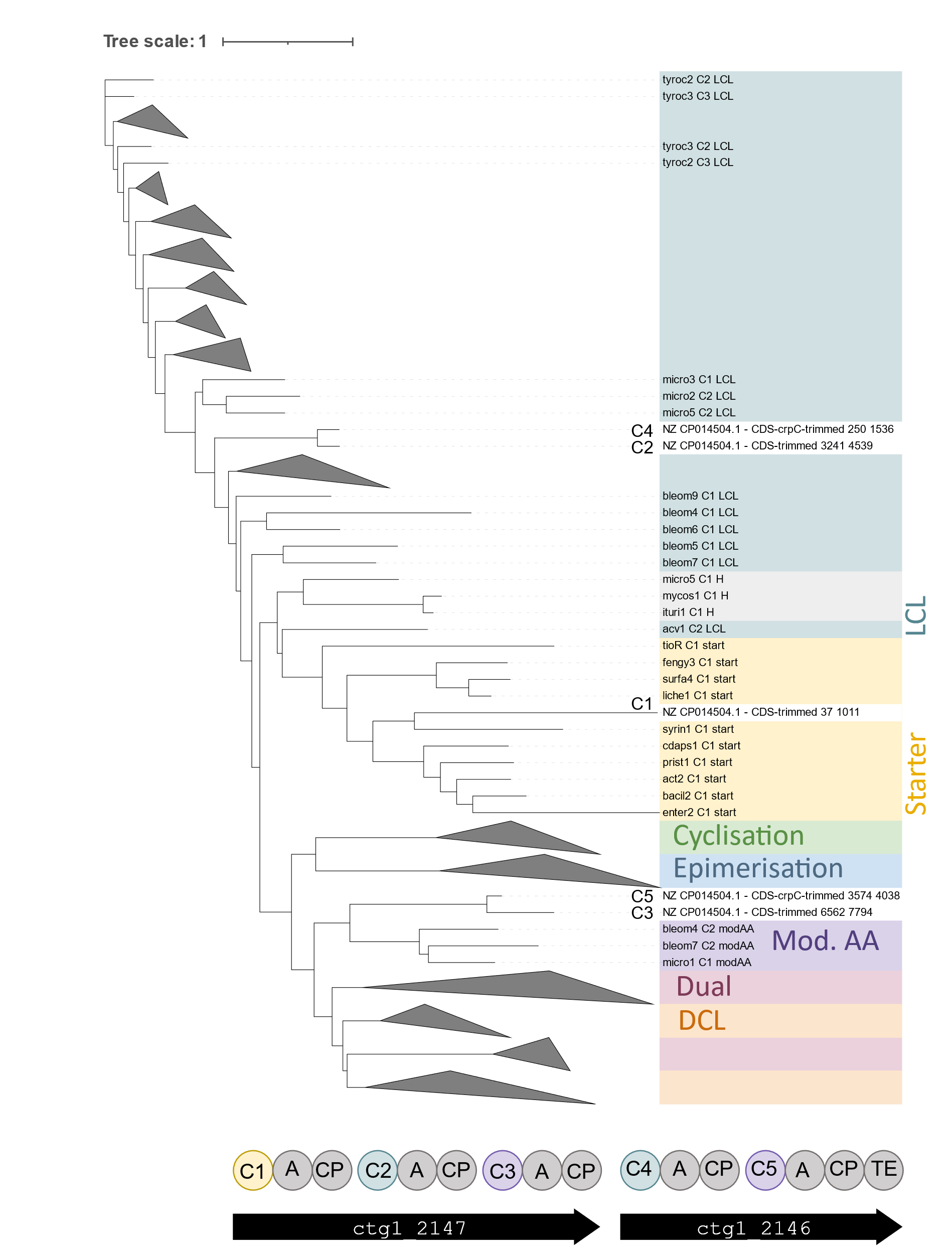 |
| --- |

## Figure S1-5. NaPDoS analysis of all C-domains predicted for the *crp* BGC. Colored clades indicate the different domain classes. Some clades are collapsed to reduce the figure size (iTOL). Below the tree the NRPS genes *crpB* (2147) and *crpC* (2146) are indicated including the different domains. C-domains are numbered and colored depending on the domain class that they are most closely related to. C1 clusters with the “starter” domain class, while C2 and C5 are predicted to be LCL-type domain classes. C3 and C5 are most closely related to the “modified AA” class, which is known to be involved in modifications of the incorporated amino acids such as dehydration processes.

| **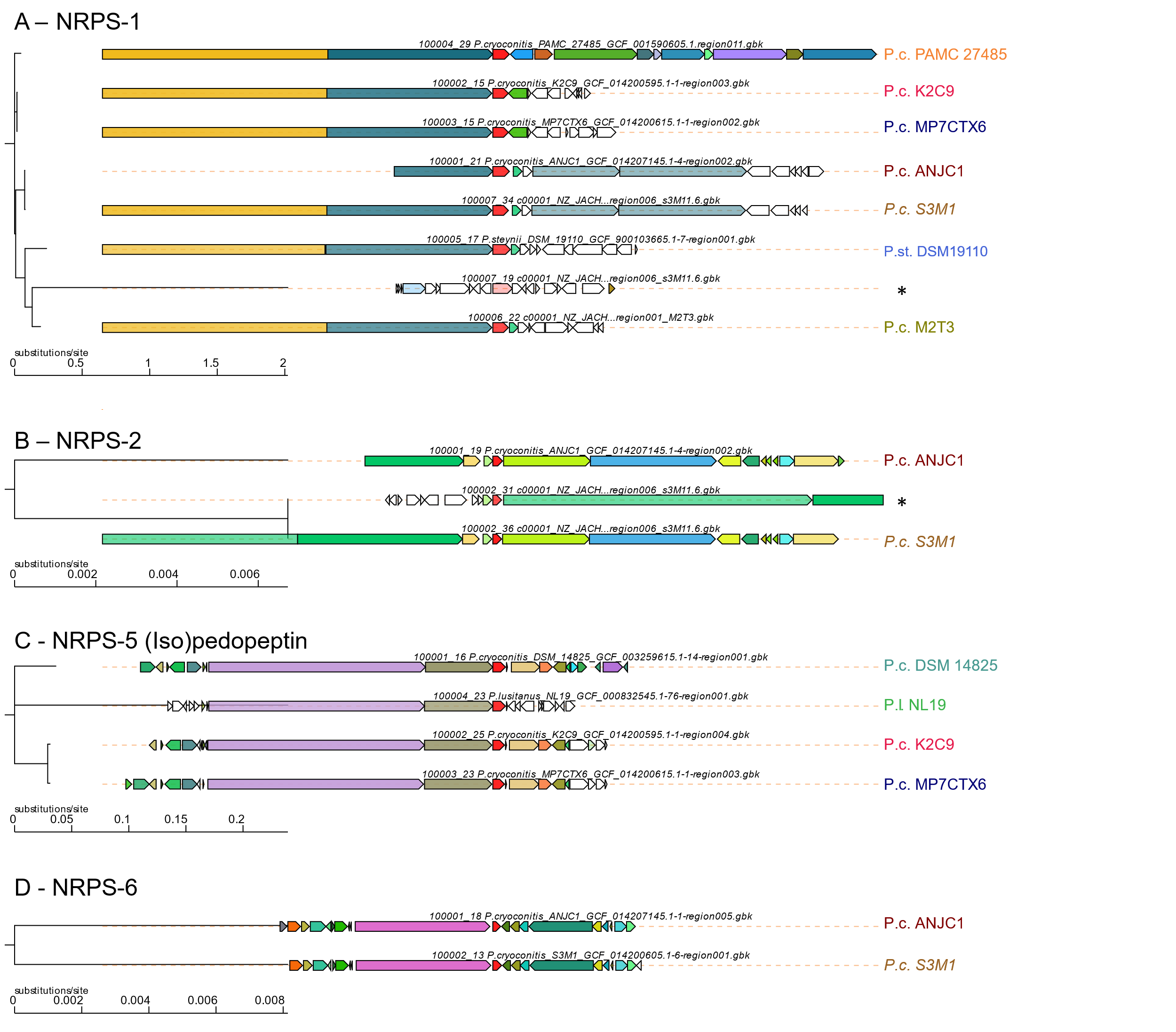** |
| --- |

## Figure S1-6. Corason Alignments of multimodular NRPS BGCs present in multiple strains. Corason was run in default settings, query genes are marked in red. BGC-containing strains are labeled at the ends of the clusters. NRPS-1 and NRPS-2 (A, B) Show fragments of clusters resulting from the fused, collectively detected BGCs by antiSMASH (marked with *). (C) Corason Alignment of Pedopeptin and putative Isopedopeptin clusters. The Pedopeptin BGC of *P. lusitanus* NL19^T^ is less similar to the others. Coloured genes indicate more modifying genes in the *P. cryoconitis* strains.

| 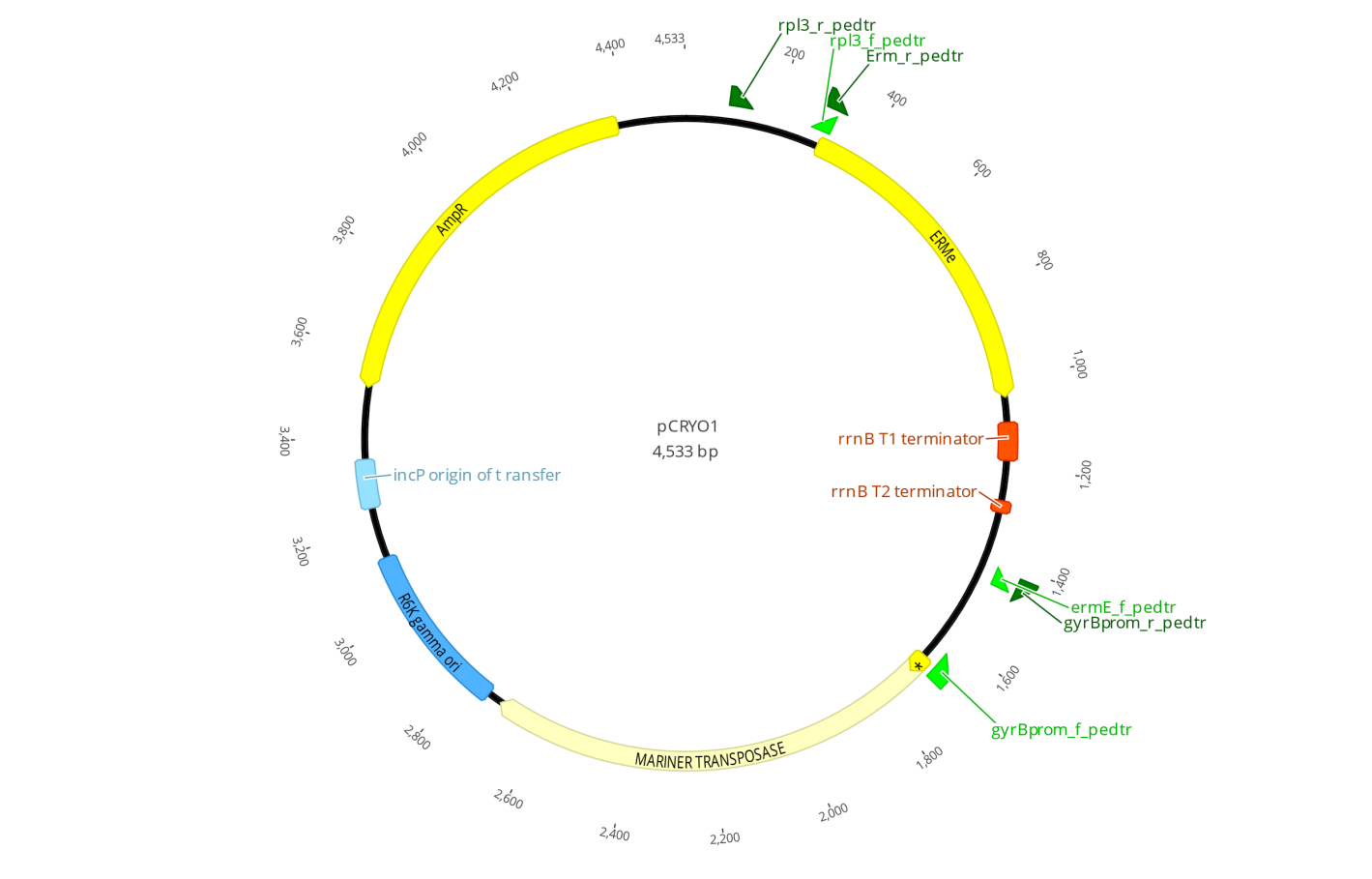 |
| --- |

## Figure S2. pCRYO1 transposon mutagenesis plasmid map. Transposase, Ampicillin (*ampR*) and Erythromycin (*ermE*) resistance Genes are represented by yellow arrows. Used primers are indicated in green. Plasmid maintenance and transfer elements are colored blue.

| 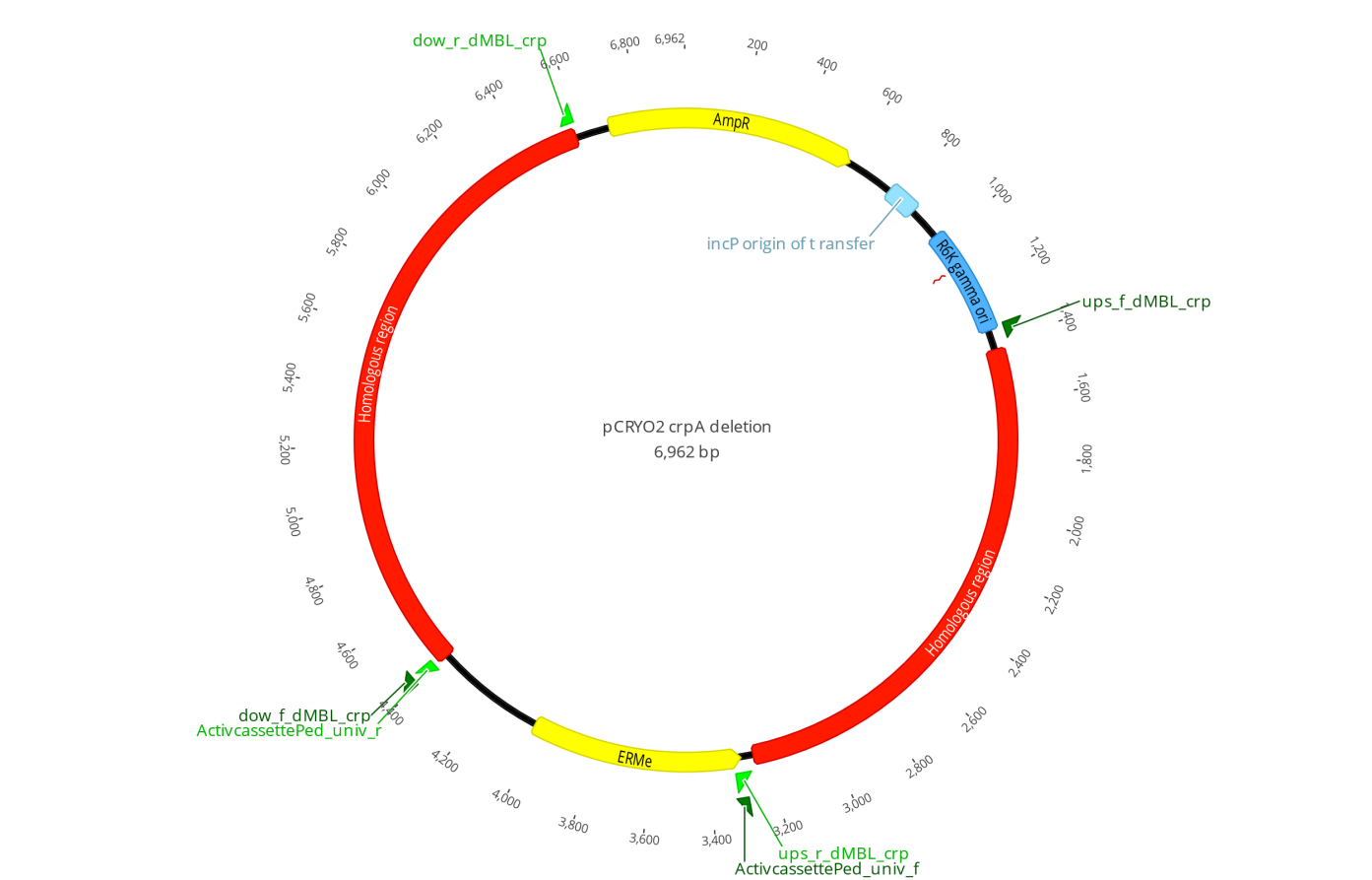 |
| --- |

## Figure S3-1. Plasmid map of pCRYO2 used for *crpA* deletion. Ampicillin (*ampR*) and Erythromycin (*ermE*) resistance genes are represented by yellow arrows. Region homologous to with *P. cryoconitis* cryopeptin BGC are colored red. Plasmid maintenance and transfer elements are colored blue. Used primers are indicated in green.

| 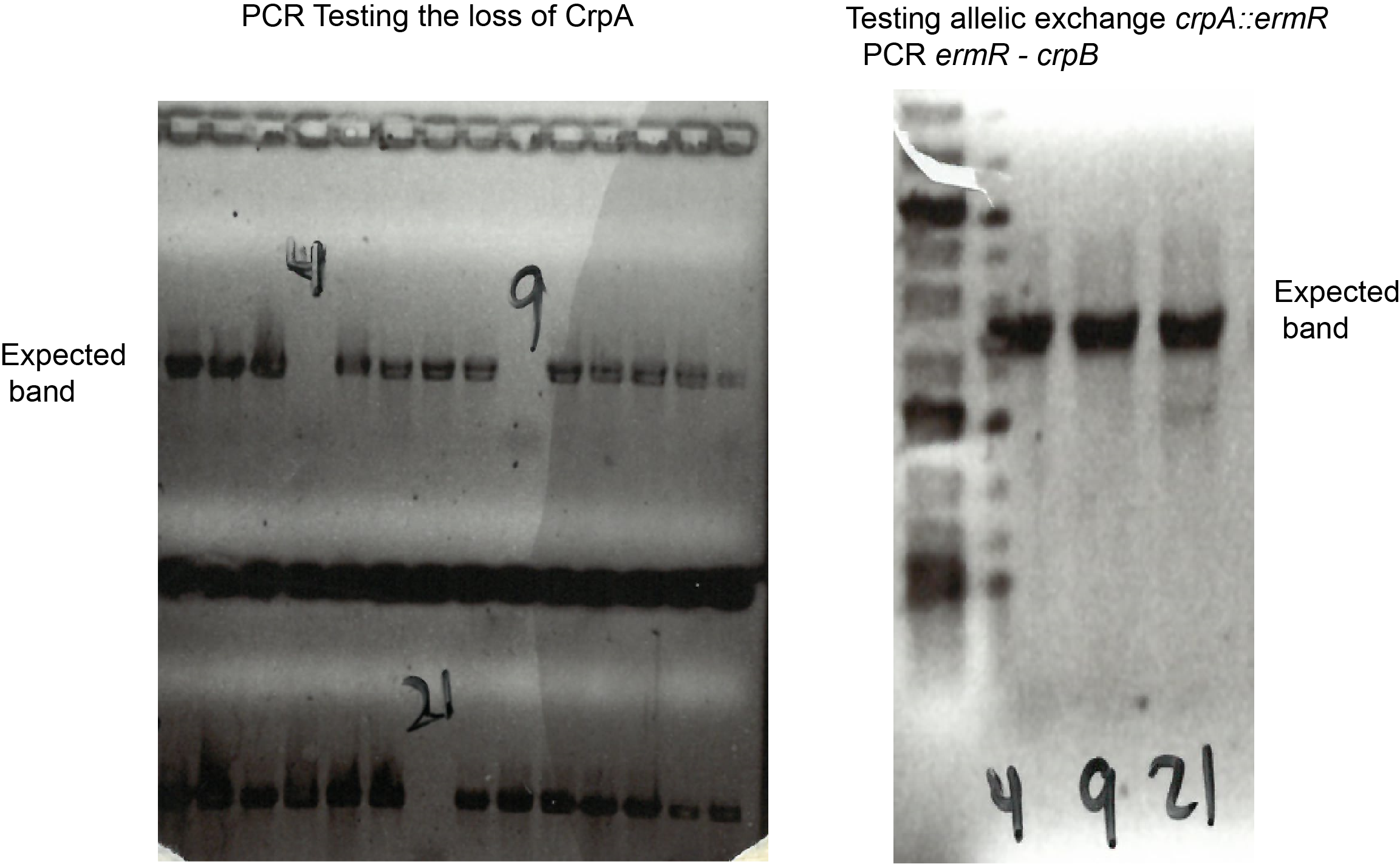 |
| --- |

## Figure S3-2. PCR of conjugant *P.cryoconitis* PAMC 27485 after allelic exchange *crpA::ermR* . PCR testing the loss of *crpA* in *P.cryoconitis* PAMC 27485 (primers target internal region of crpA).

| 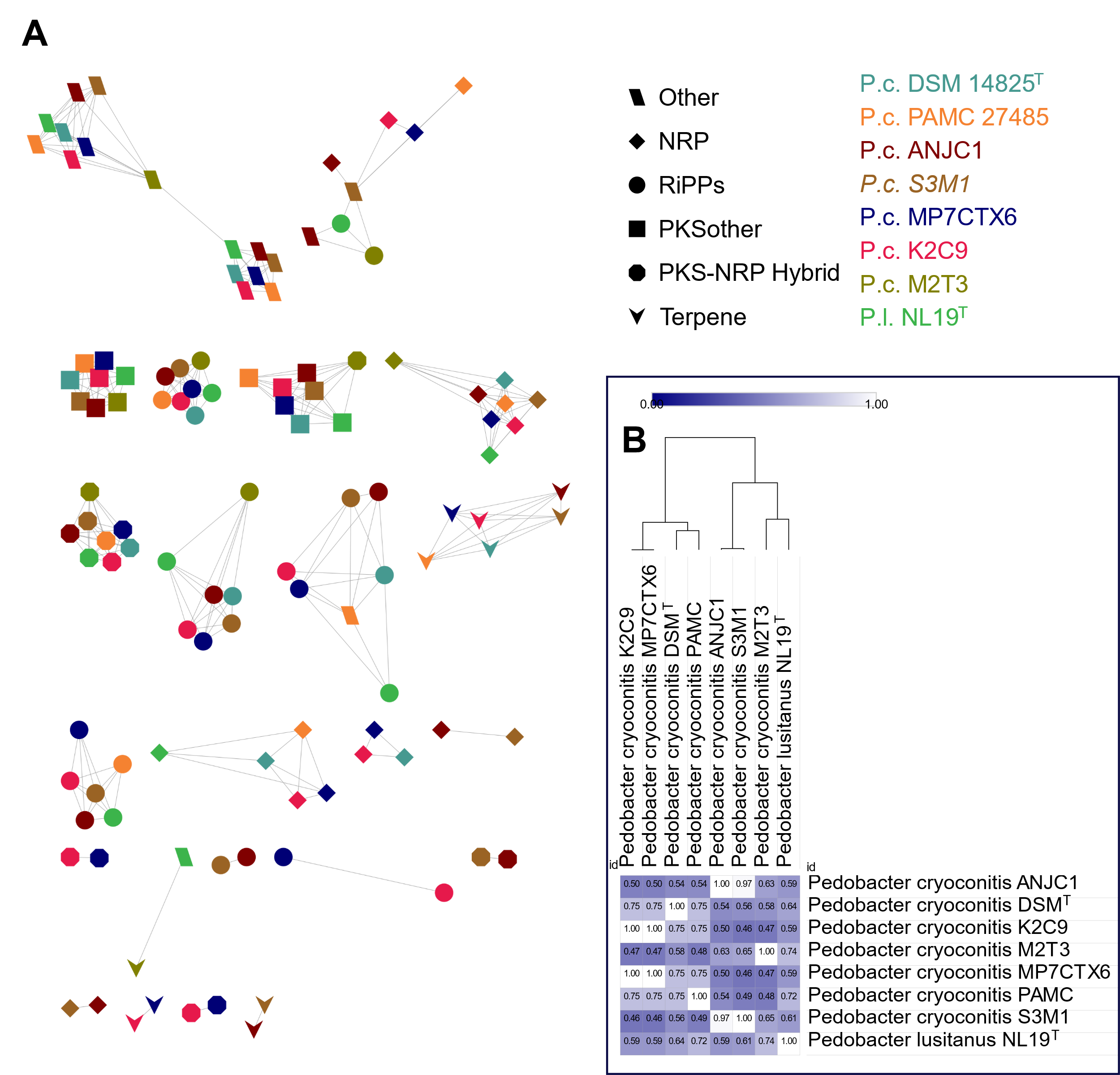 |
| --- |

## Figure S4. A BiG-SCAPE analysis of anti-SMASH detected BGCS in the 8 strains associated to the *P. cryoconitis* clade (Cutoffs for GCFs: 0.6). The node shape indicates the BGC type, and the node color is the strain in the BGC that was detected. Numbers at nodes describe the Big-SCAPE-determined gene cluster family. Besides the shown clusters, 48 singletons were determined. No BGC automatically clusters with any known MiBiG Reference cluster. B Heat Map illustrating the cosine similarity of the overall BGC composition in the *P. cryoconitis* branch. The calculated tree is based on that similarity and highlights the similar BGC composition in the groups containing for one K2C9 and MP7CTX6 and for another ANJC1 and S3M1.

| 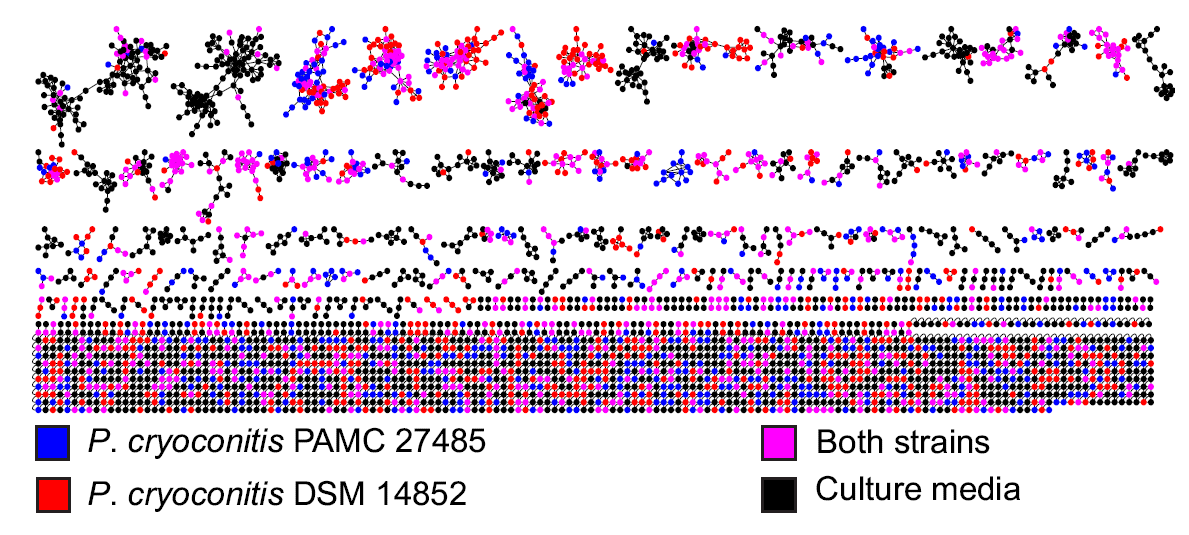 |
| --- |

## Figure S5. Molecular networking analysis of a *P. cryoconitis* PAMC_27485 and *P. cryoconitis* DSM 14825^T^ extracts using GNPS. Both strains were cultured in 6 different media (NB, LB, R2A, POM, MYE, TSB) and extracted using 3 different methods (butanol and ethyl acetate liquid-liquid extraction and C18 resin solid phase extraction. Blue and red circles indicate ions that are unique for *P. cryoconitis* PAMC_27485 and *P. cryoconitis* DSM 14825^T^ respectively. Pink circles indicated ions that are common for both strains. Black circles represent ions found in any of the culture media. Cryopeptin containing clusters are squared.

| 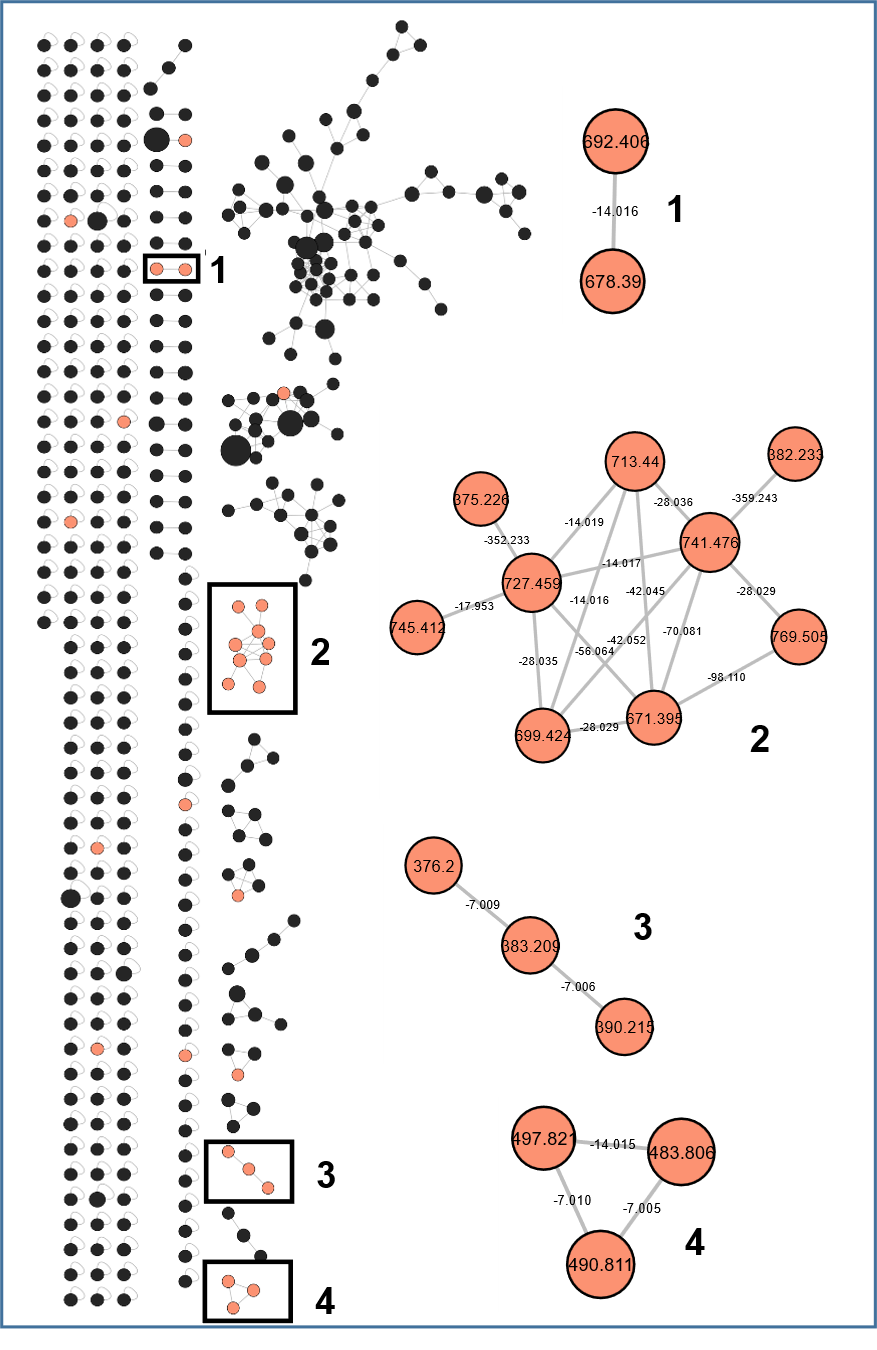 |
| --- |

## Figure S6. Molecular network analysis of a *P. cryoconitis* PAMC 27485 Wild type and ∆*crpA* strains extracts. Highlighted in orange ions that are present only in WT and not in the deletion mutant. Cryopeptin-containing clusters are numbered and zoomed. Heptapeptidic cryopeptins (K-N) were identified in cluster 4 ([M+2H]^2+^). Cryopeptins A, C, D, E, F, G, H ([M+H]^+^) were identified in cluster 2. Cluster 1 and 3 ions were not structurally characterized. Black circles represent ions that are common for both WT and ∆*crpA* strains.

|  |
| --- |
|  |

| 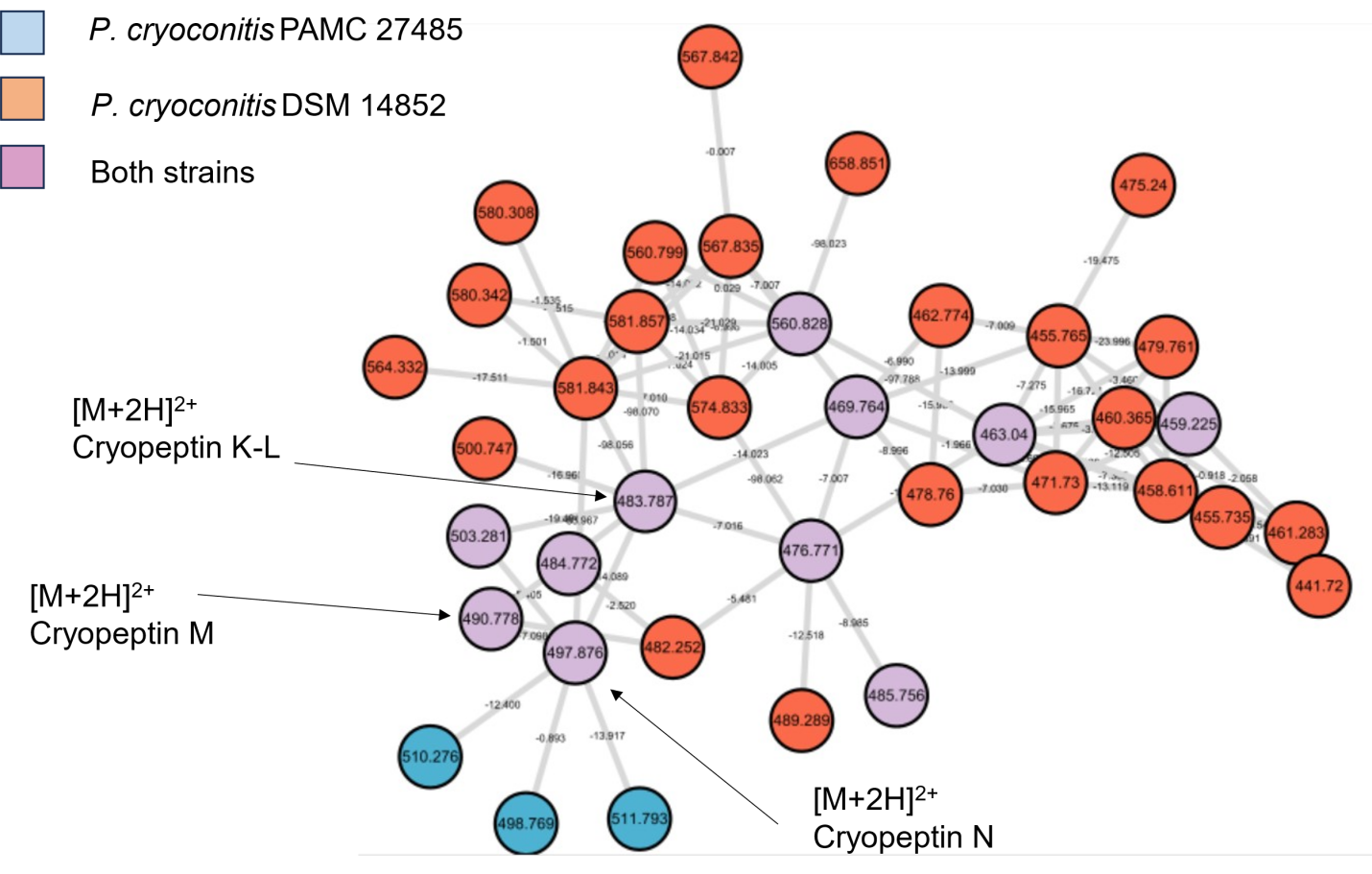 |
| --- |

## Figure S7. Molecular network containing heptapeptidic cryopeptins from both *P. cryoconitis* PAMC_27485 and *P. cryoconitis* DSM 14825^T^ extracts. Both strains were cultured in 6 different media (NB, LB, R2A, POM, MYE, TSB) and extracted using 3 different methods (butanol and ethyl acetate liquid-liquid extraction and C18 resin solid phase extraction. Blue and orange circles indicate ions that are unique for *P. cryoconitis* PAMC_27485 and *P. cryoconitis* DSM 14825^T^ respectively. Purple circles indicated ions that are common for both strains. Structurally characterized cryopeptins are indicated with arrows.

| 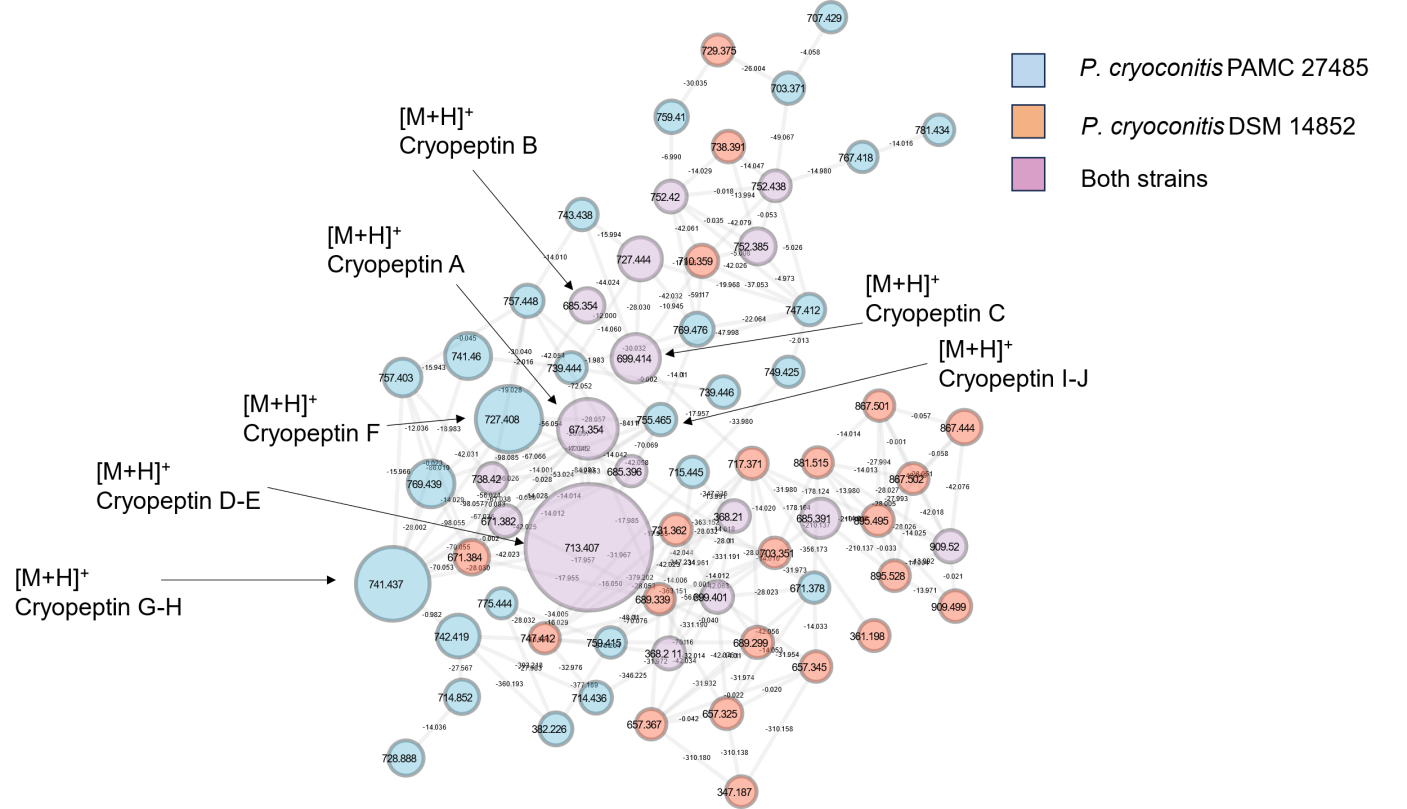 |
| --- |
|  |

## Figure S8. Molecular network containing pentapeptidic cryopeptins from both *P. cryoconitis* PAMC 27485 and *P. cryoconitis* DSM 14825^T^ extracts. Both strains were cultured in 6 different media (NB, LB, R2A, POM, MYE, TSB) and extracted using 3 different methods (butanol and ethyl acetate liquid-liquid extraction and C18 resin solid phase extraction. Blue and red circles indicate ions that are unique for *P. cryoconitis* PAMC_27485 and *P. cryoconitis* DSM 14825^T^ respectively. Purple circles indicated ions that are common for both strains. Structurally characterized cryopeptins are indicated with arrows.

| 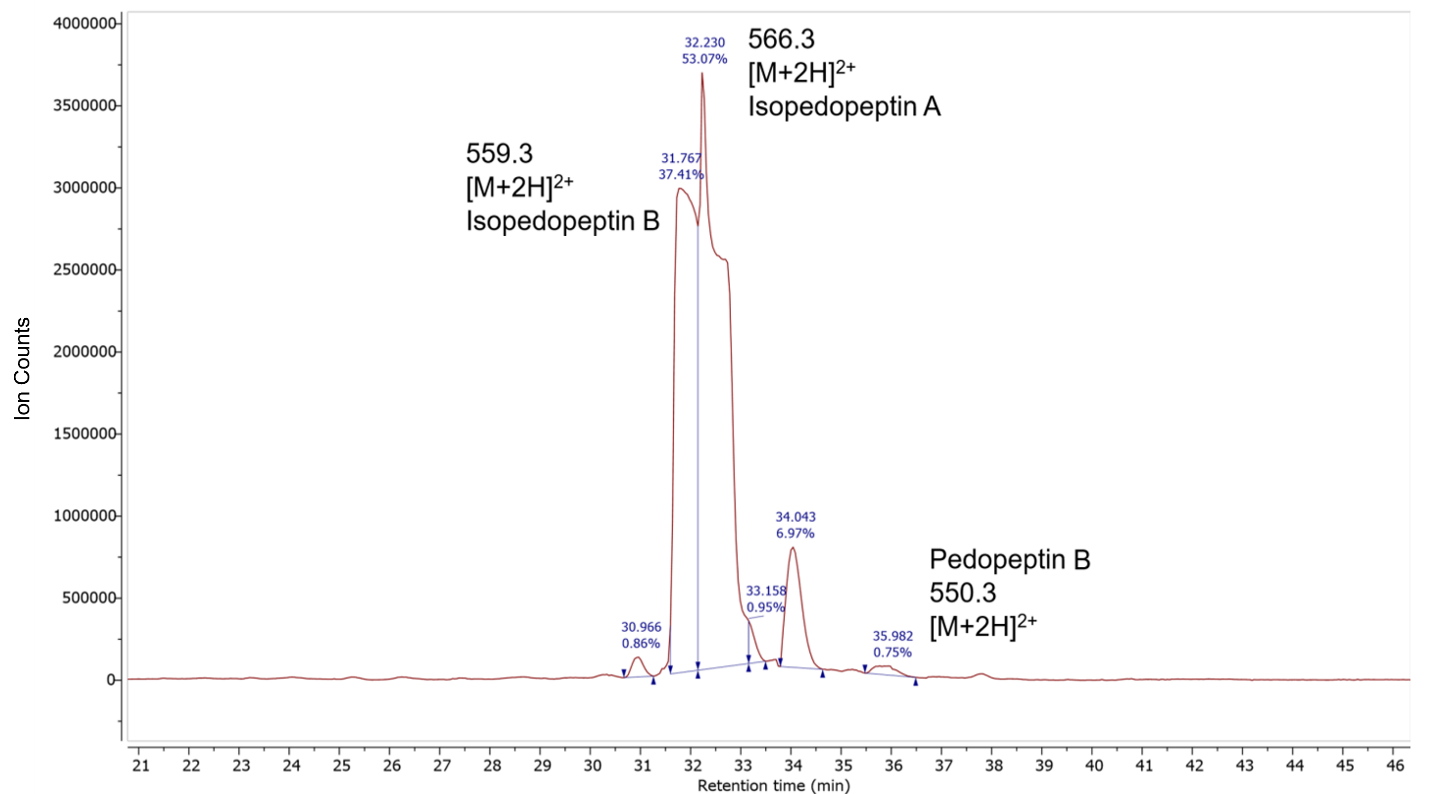 |
| --- |

## Figure S9. UPLC-HR-ESI-MS analysis of a *P. cryoconitis* DSM 14825^T^ extract. Extracted ion chromatograms of pedopeptin B and isopedopeptins A and B showing the respective compound peaks with their m/z value [M+2H]^2+^. All Δ m/z ≤ 0.051


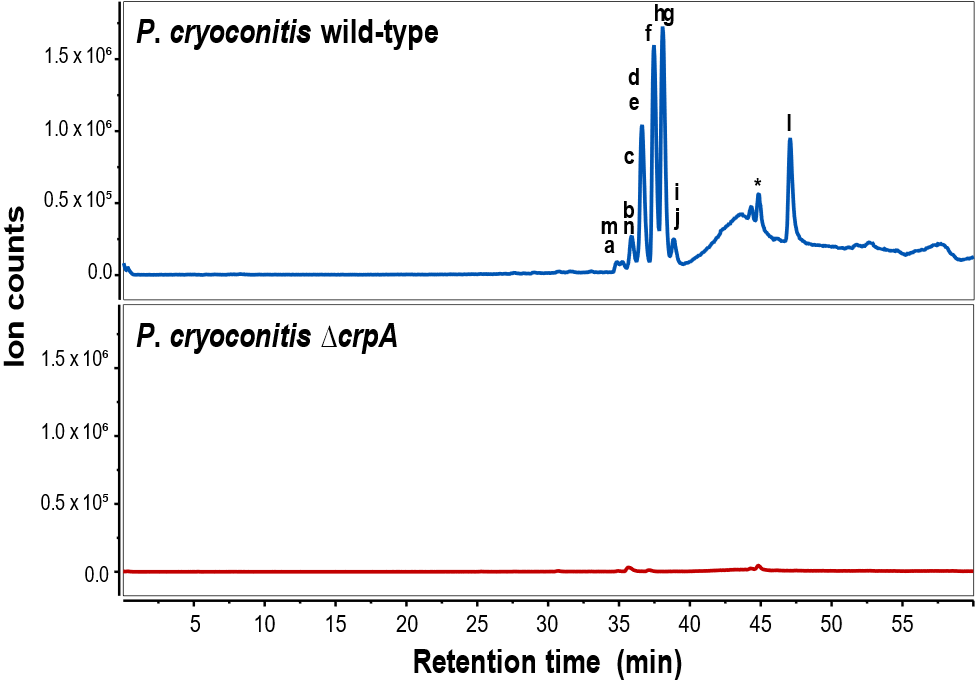


## Figure S10. UPLC-HR-ESI-MS analysis of *P. cryoconitis* PAMC 27485 wild type *(blue)* and *P. cryoconitis* PAMC 27485 *ΔcrpA* (red)extracts. Extracted ion chromatograms of cryopeptins A-N are shown.


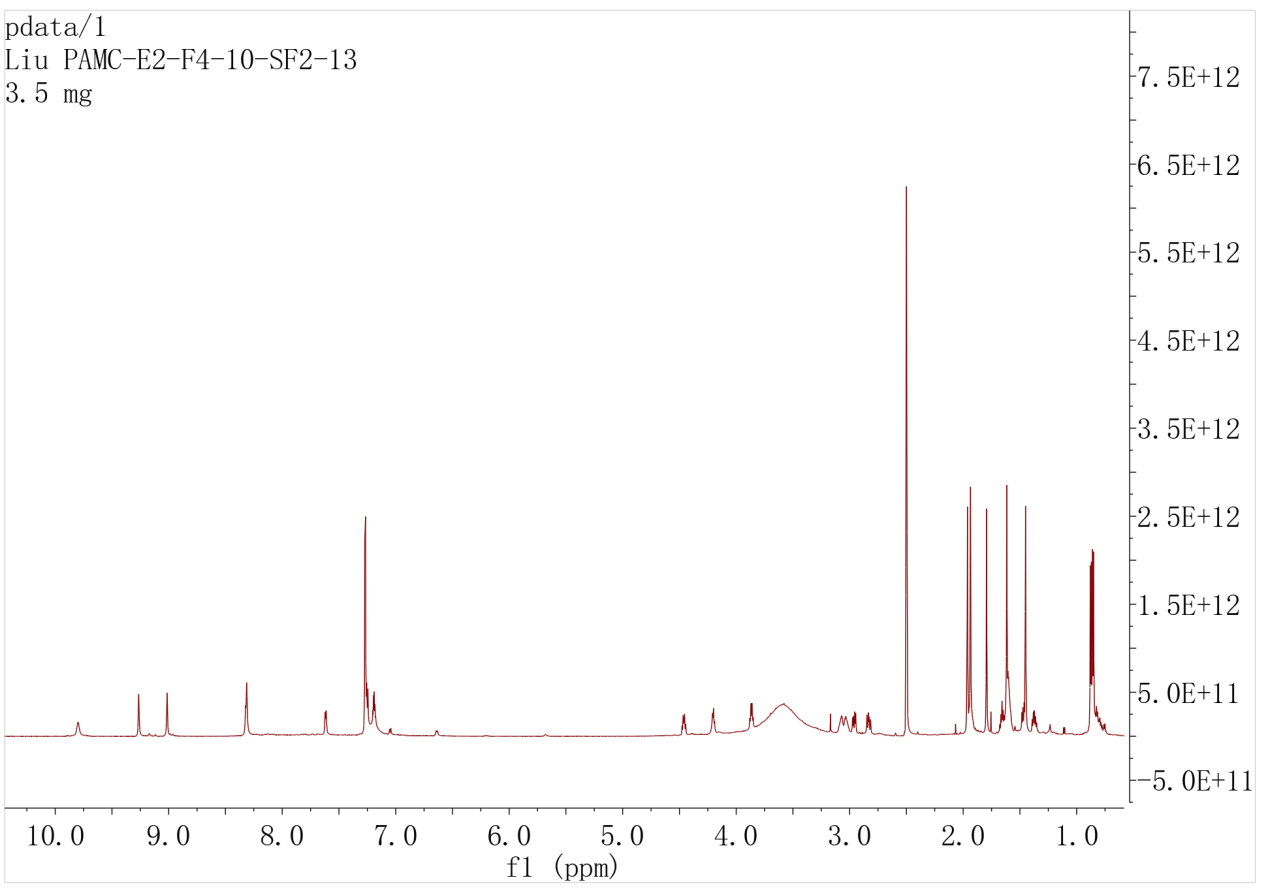


## Figure S11-1. ^1^H NMR spectrum of 1 (DMSO-*d*_6_,700MHz)


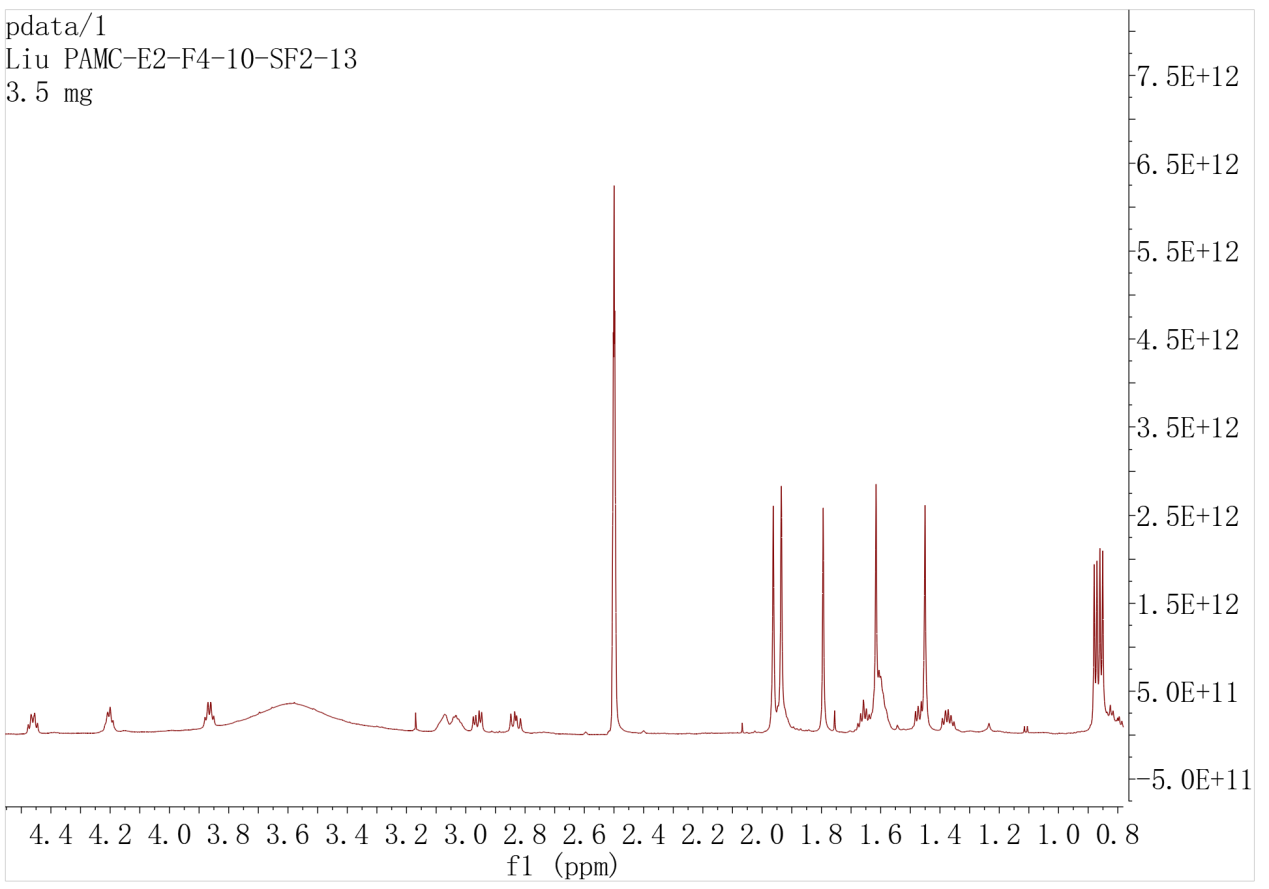


## Figure S11-1-1. ^1^H NMR spectrum of 1 with expansion


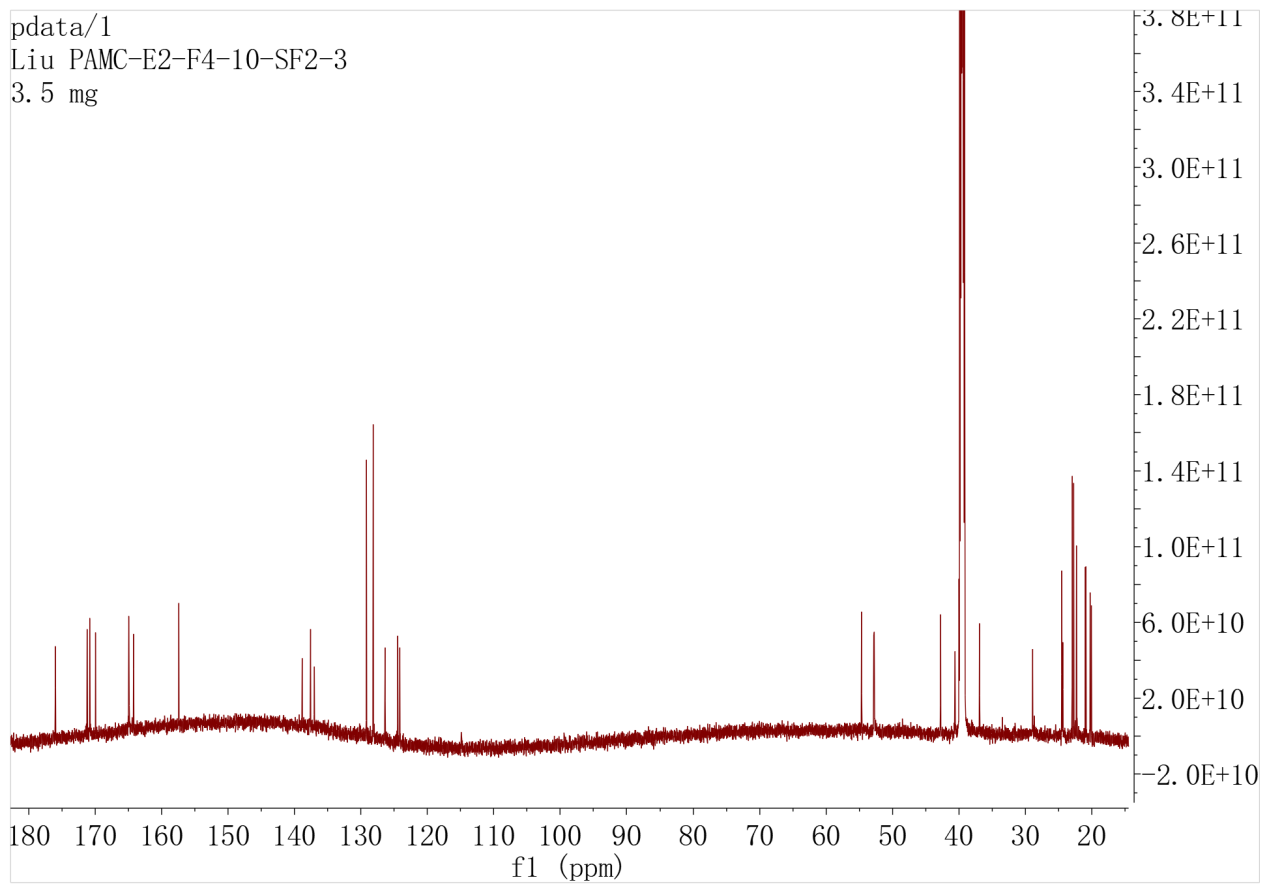


## Figure S11-2. ^13^C NMR spectrum of 1 (DMSO-*d*_6_, 176MHz)


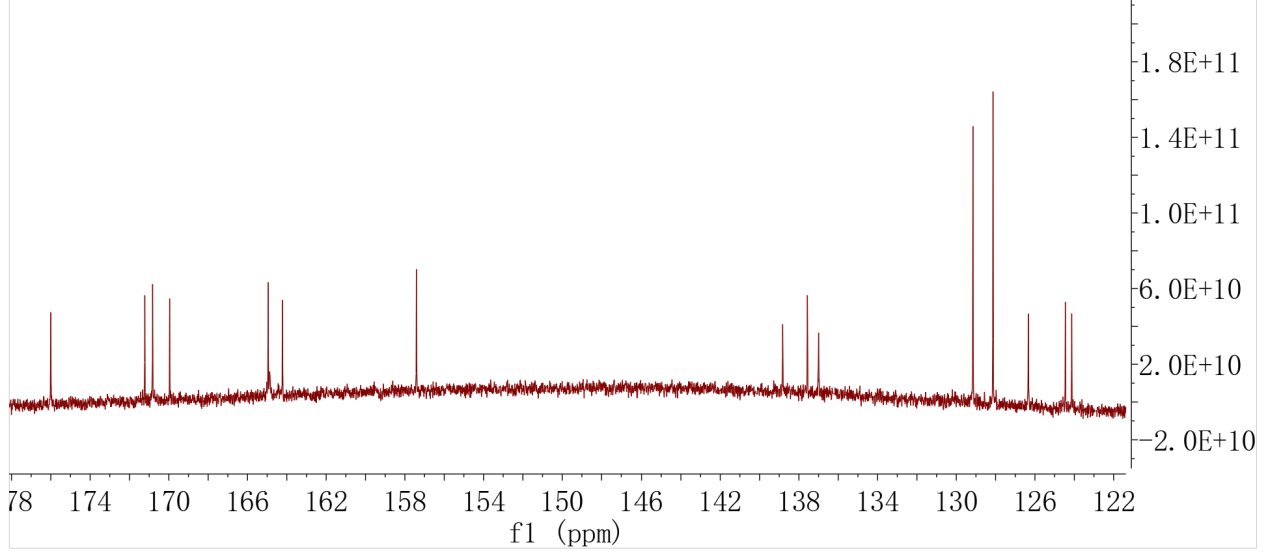


## Figure S11-2-1. ^13^C NMR spectrum of 1 with expansion


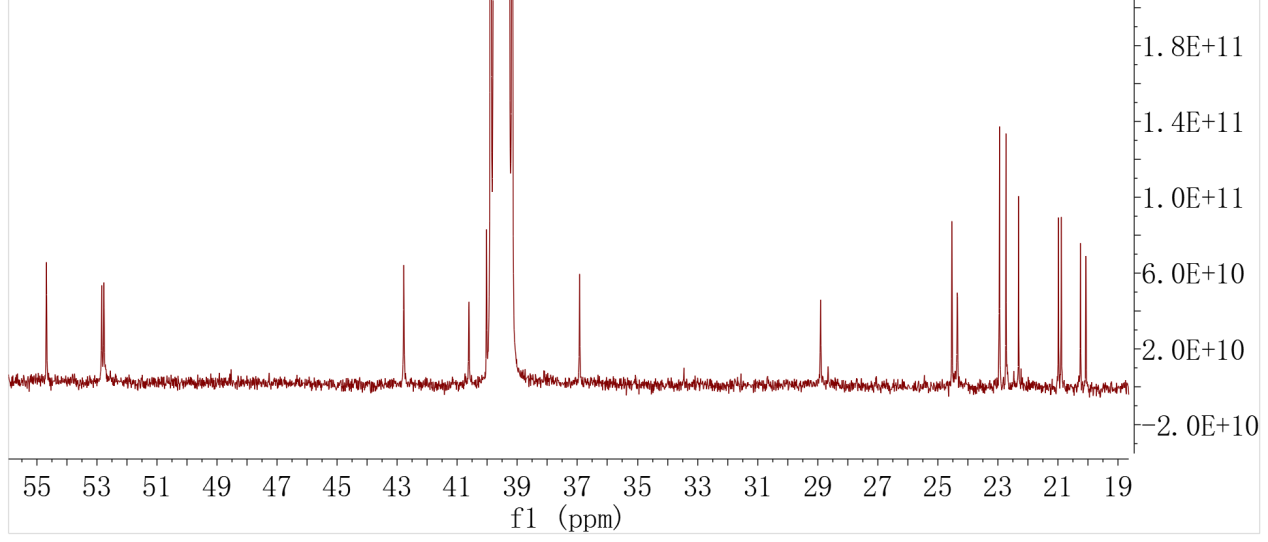


## Figure S11-2-2. ^13^C NMR spectrum of 1 with expansion


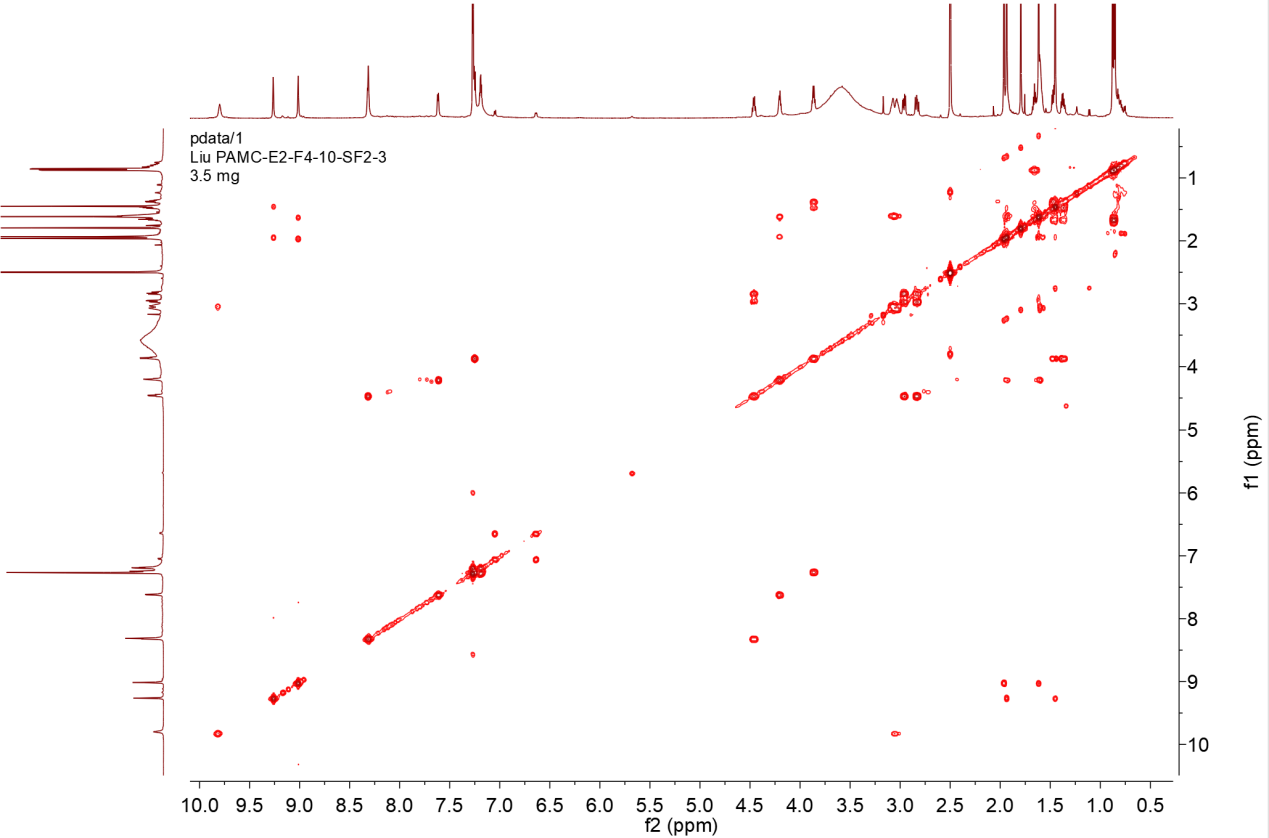


## Figure S11-3. COSY spectrum of 1


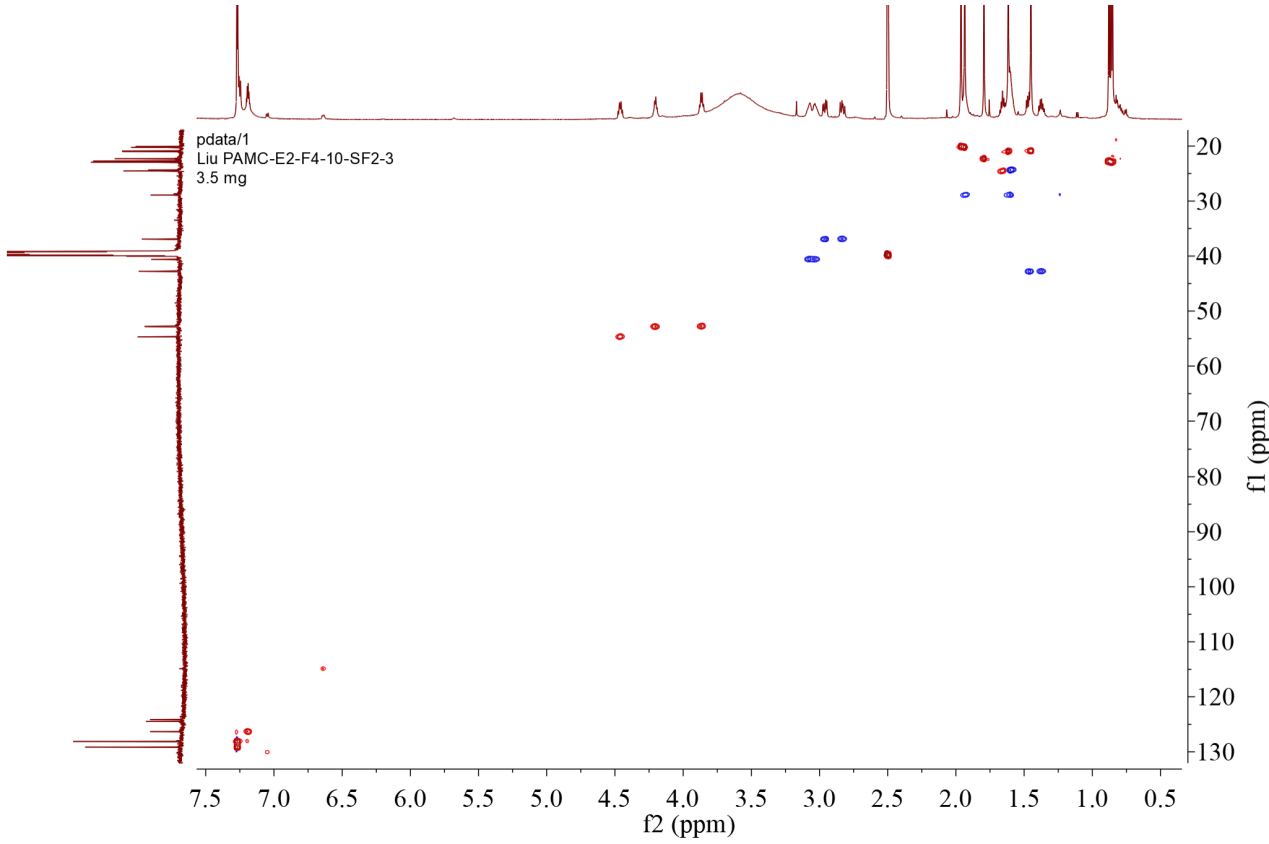


## Figure S11-4. Multiplicity-edited HSQC spectrum of 1


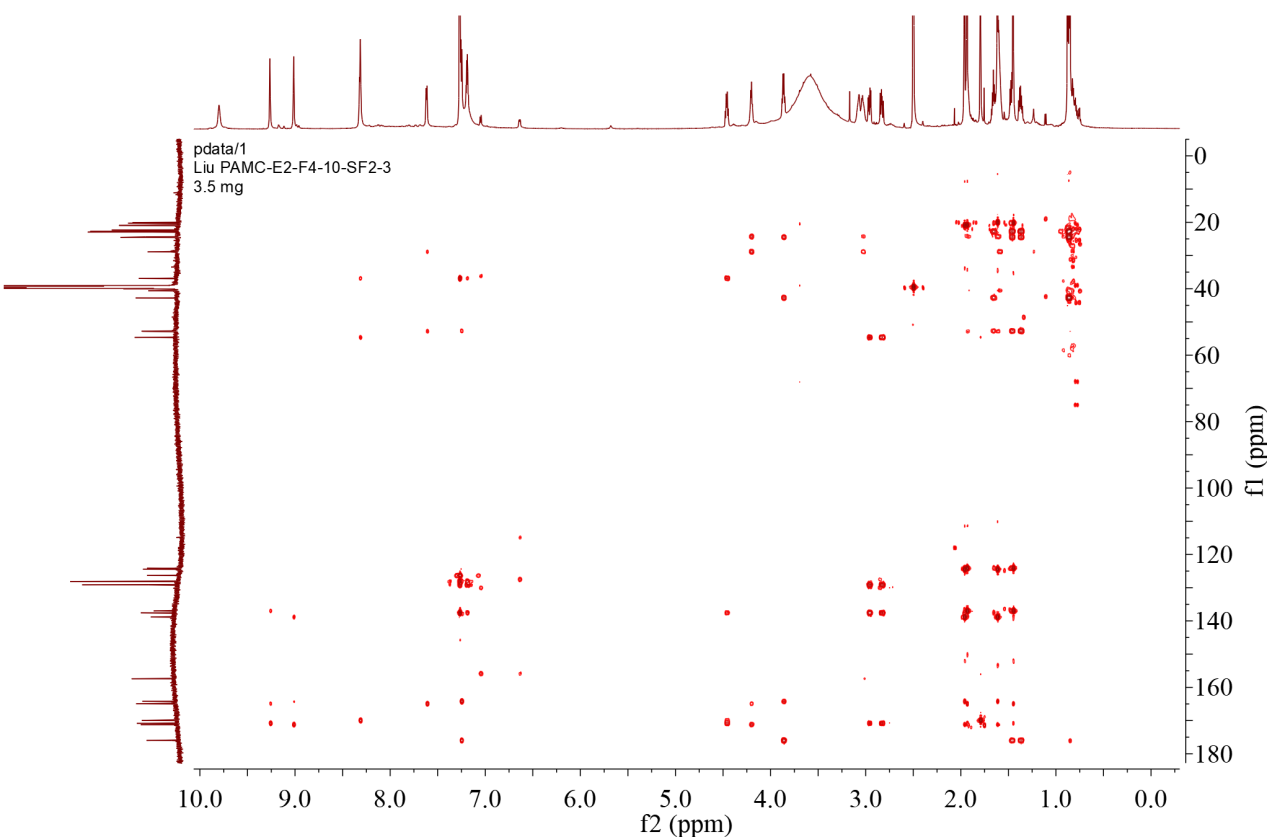


## Figure S11-5. HMBC spectrum of 1


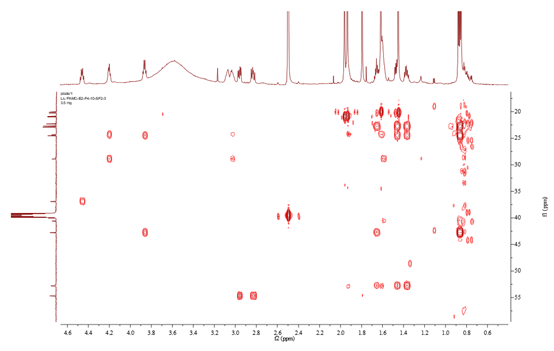


## Figure S11-5-1. HMBC spectrum of 1 with expansion


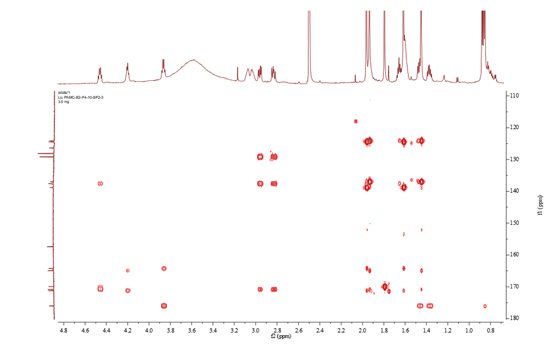


## Figure S11-5-2. HMBC spectrum of 1 with expansion


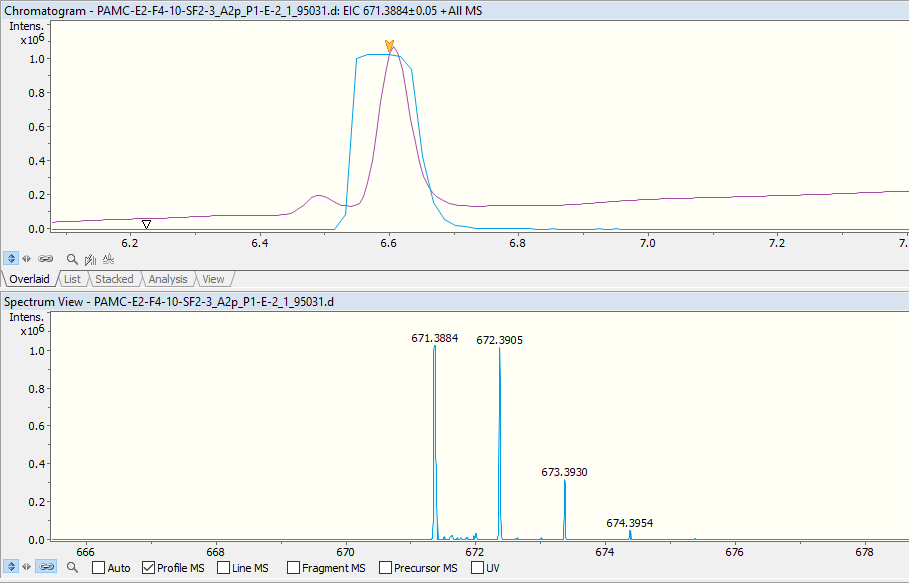


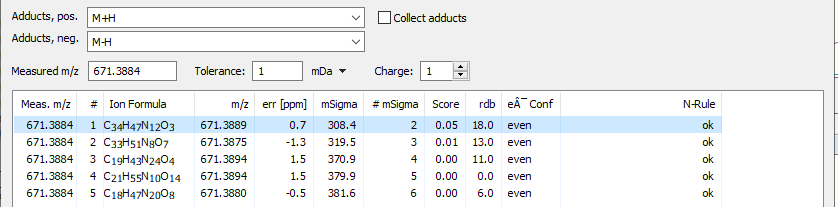


## Figure S11-6. UPLC-HR-ESI-MS spectrum of 1


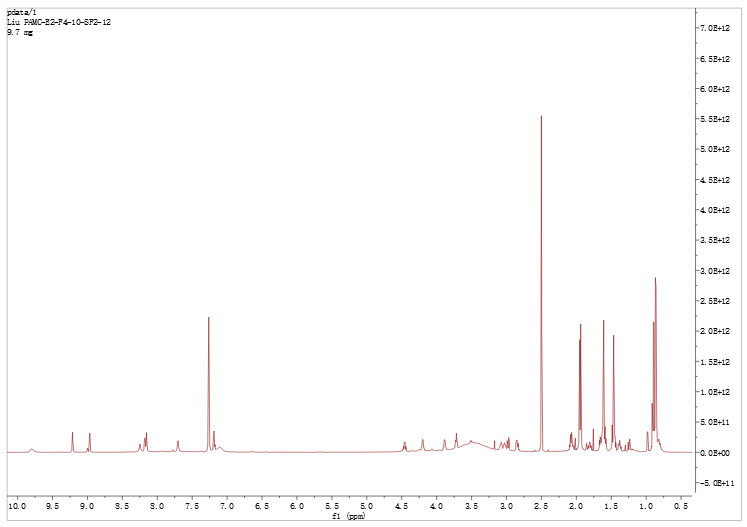


## Figure S12-1. ^1^H NMR spectrum of 2 (DMSO-*d*_6_,700MHz)


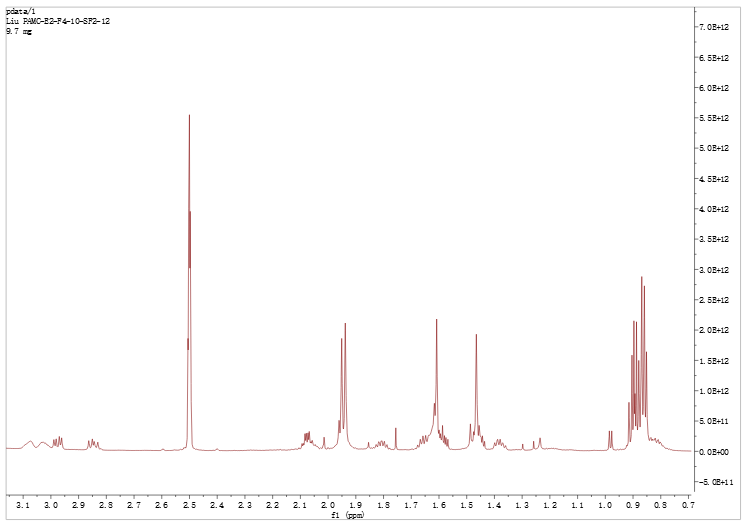


## Figure S12-1-1. ^1^H NMR spectrum of 2 with expansion


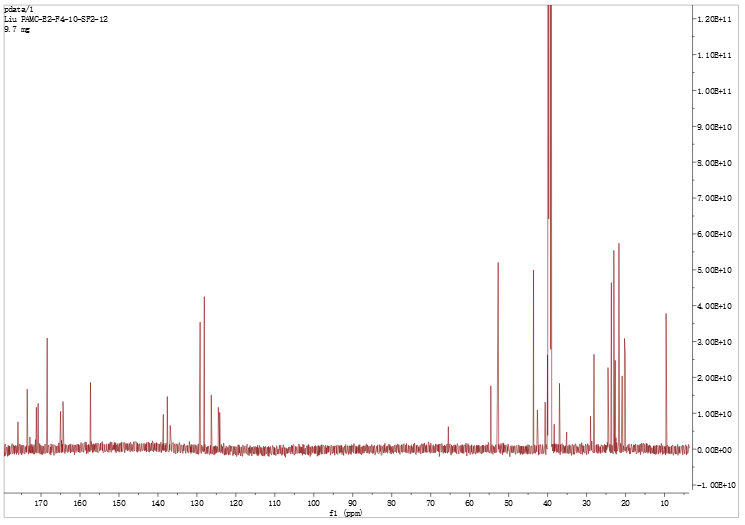


## Figure S12-2. ^13^C NMR spectrum of 2 (DMSO-*d*_6_, 176MHz)


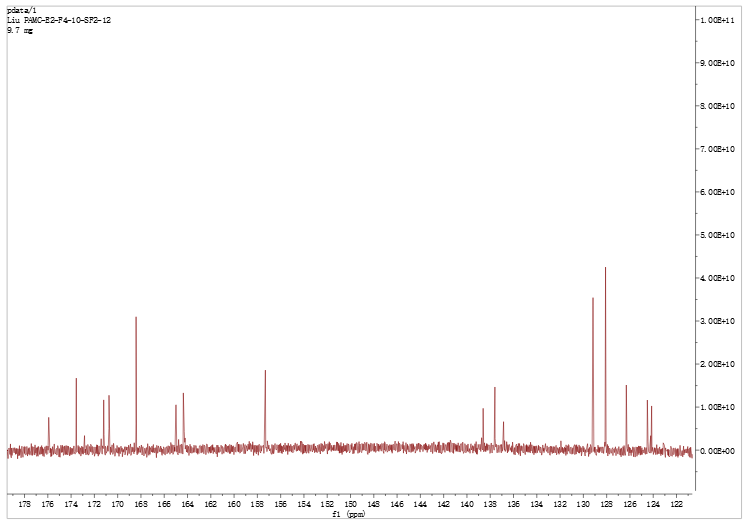


## Figure S12-2-1. ^13^C NMR spectrum of 2 with expansion


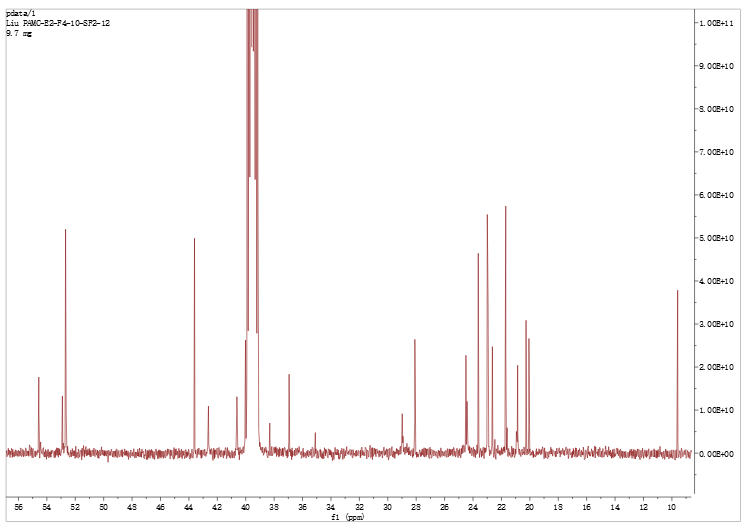


## Figure S12-2-2. ^13^C NMR spectrum of 2 with expansion


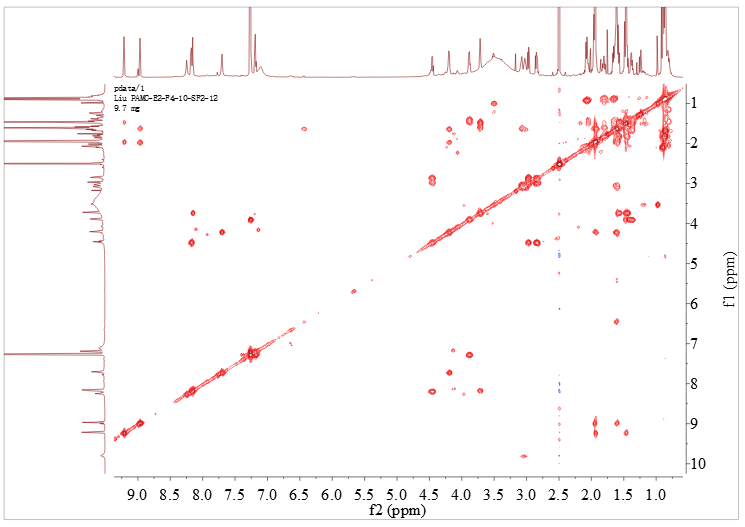


## Figure S12-3. COSY spectrum of 2


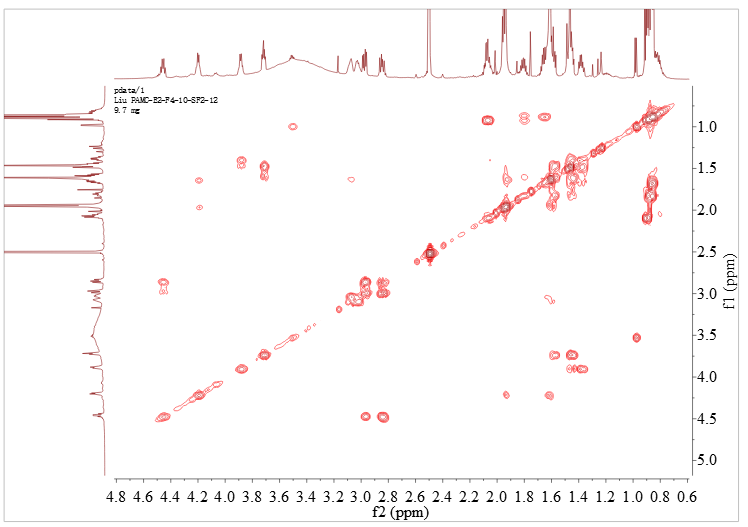


## Figure S12-3-1. COSY spectrum of 2 with expansion


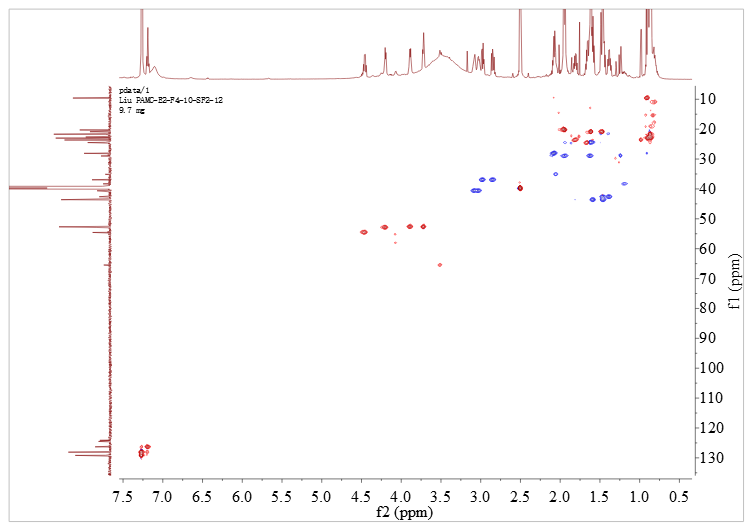


## Figure S12-4. Multiplicity-edited HSQC spectrum of 2


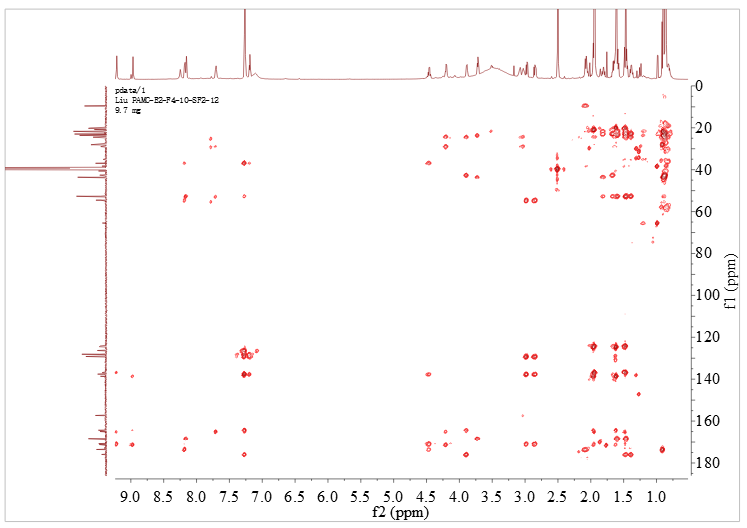


## Figure S12-5. HMBC spectrum of 2


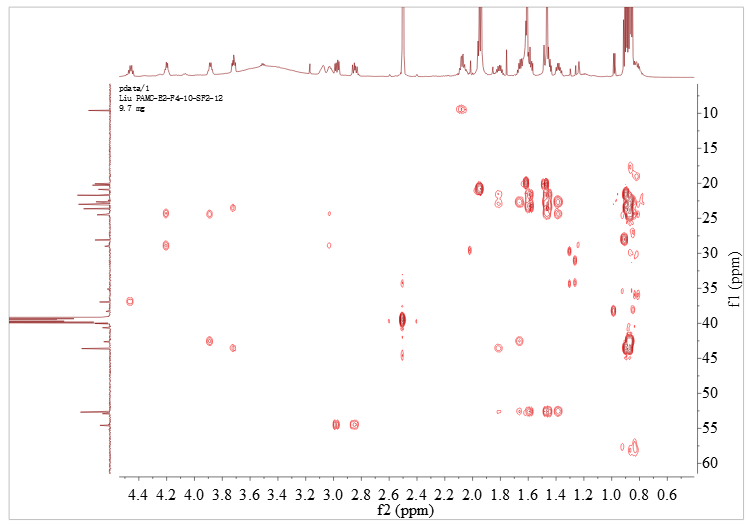


## Figure S12-5-1. HMBC spectrum of 2 with expansion


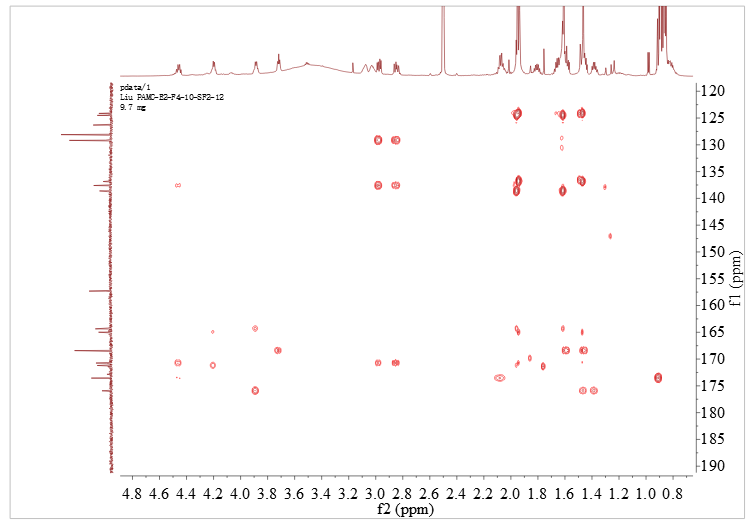


## Figure S12-5-2. HMBC spectrum of 2 with expansion


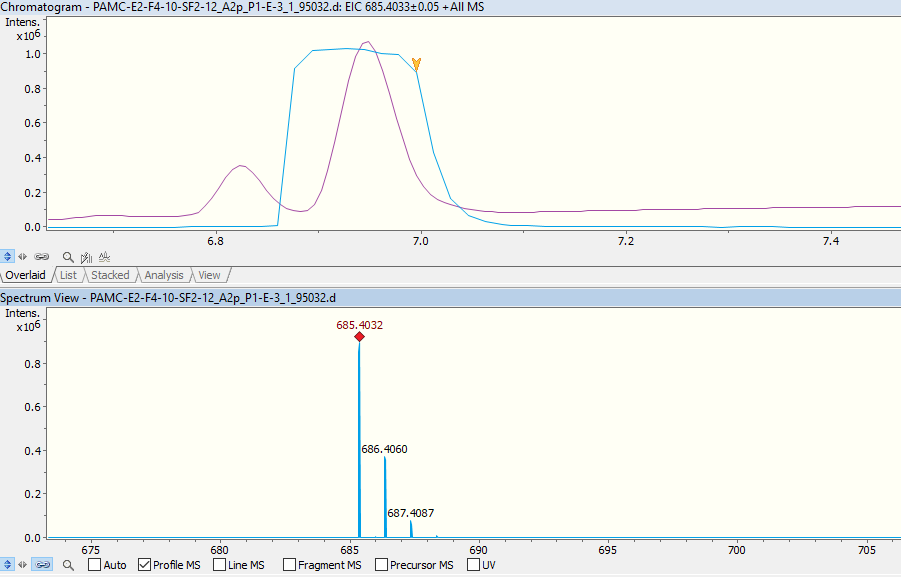


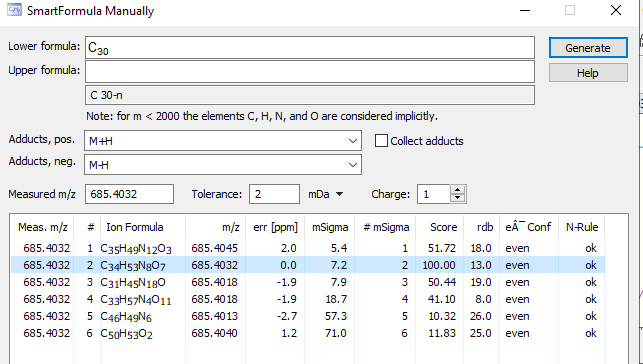


## Figure S12-6. UPLC-HR-ESI-MS spectrum of 2


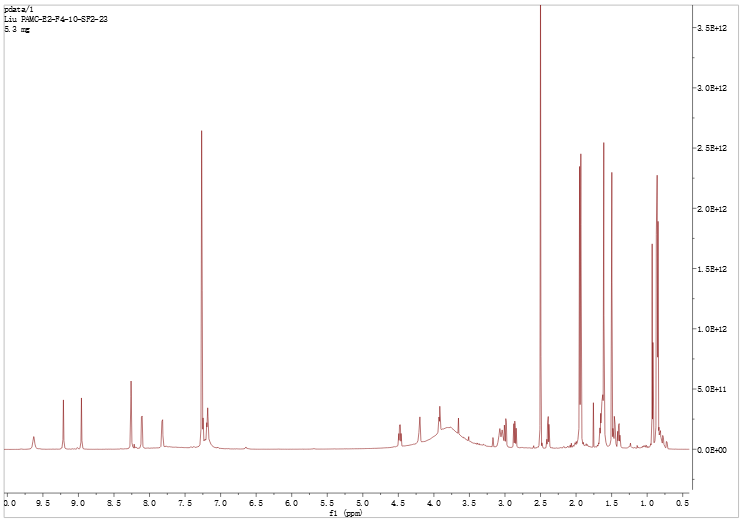


## Figure S13-1. ^1^H NMR spectrum of 3 (DMSO-*d*_6_,700MHz)


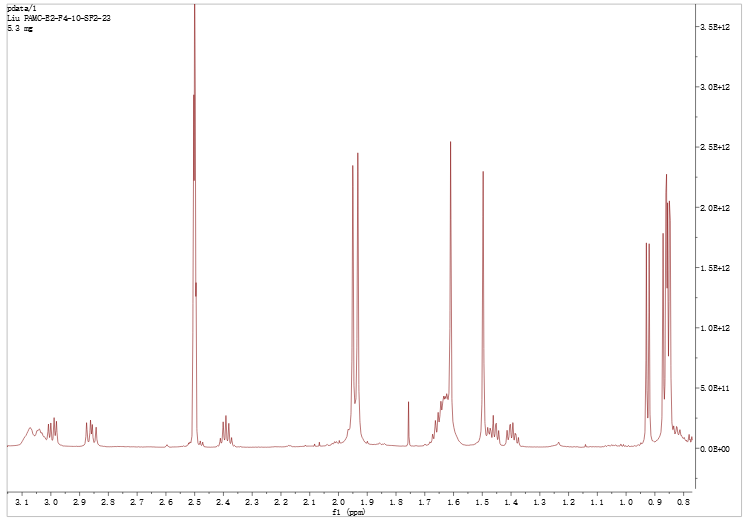


## Figure S13-1-1. ^1^H NMR spectrum of 3 with expansion


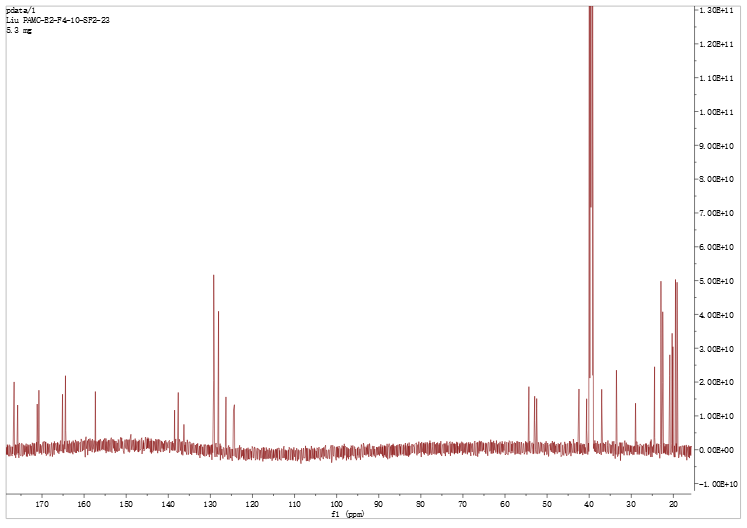


## Figure S13-2. ^13^C NMR spectrum of 3 (DMSO-*d*_6_, 176MHz)


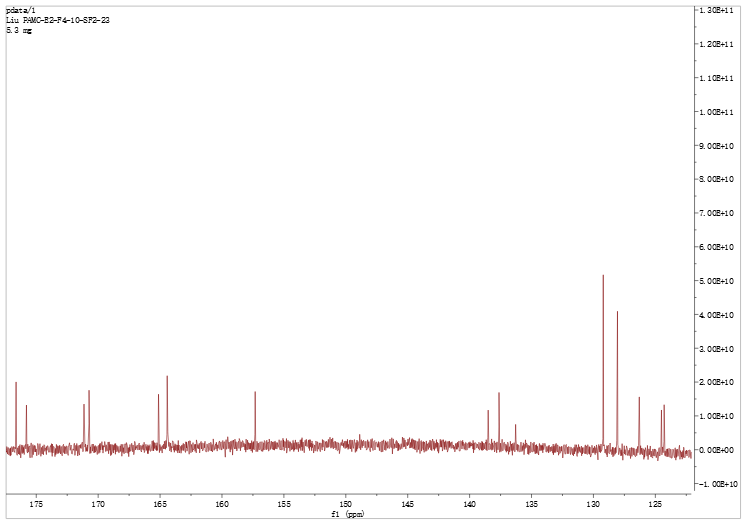


## Figure S13-2-1. ^13^C NMR spectrum of 3 with expansion


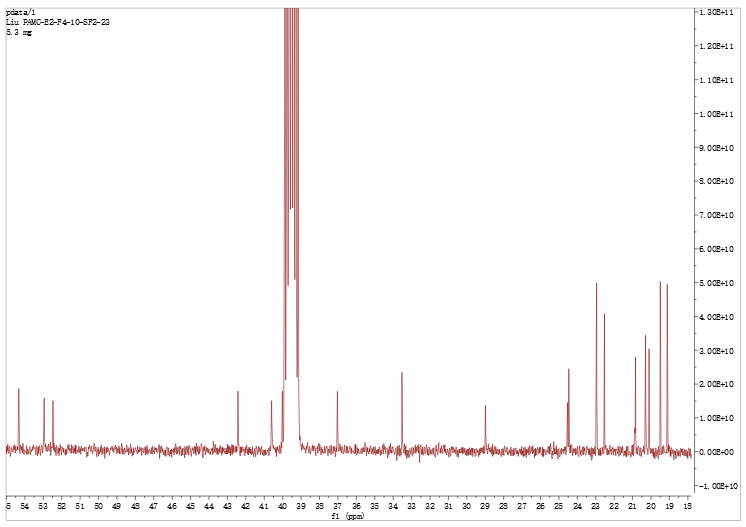


## Figure S13-2-2. ^13^C NMR spectrum of 3 with expansion


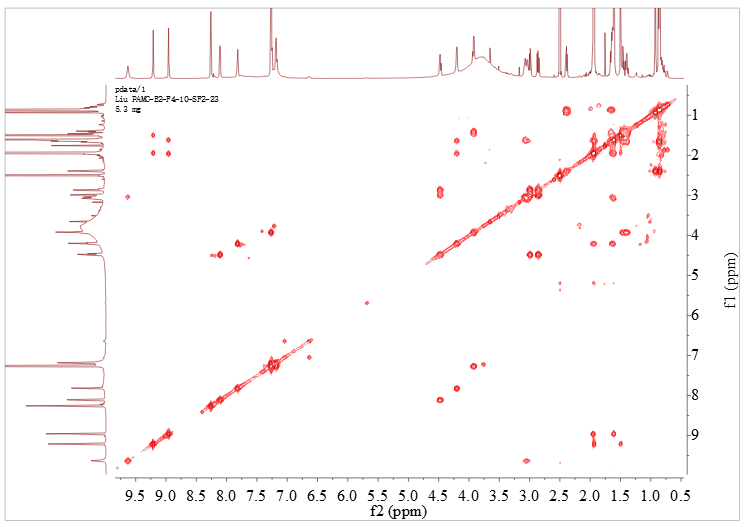


## Figure S13-3. COSY spectrum of 3


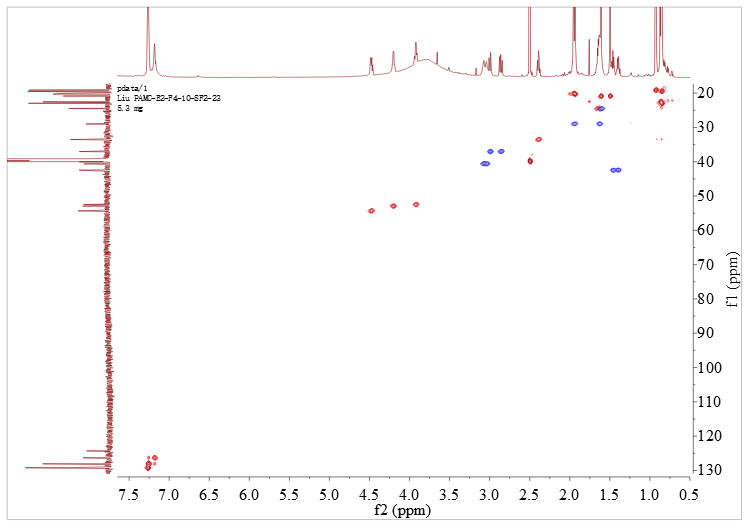


## Figure S13-4. Multiplicity-edited HSQC spectrum of 3


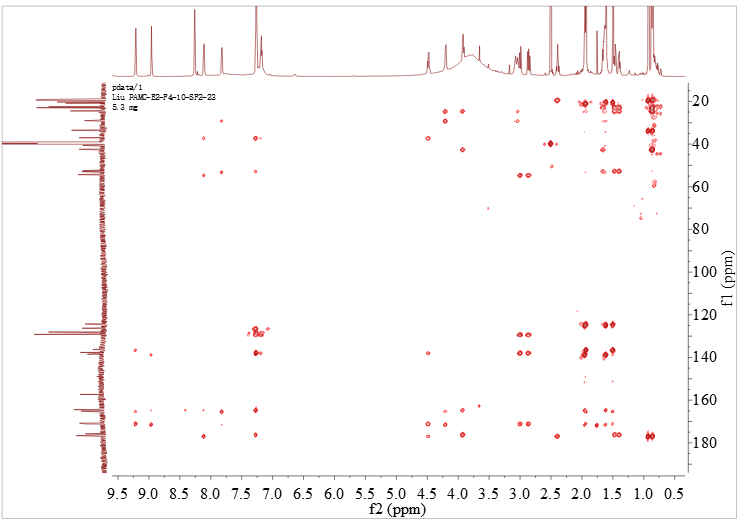


## Figure S13-5. HMBC spectrum of 3


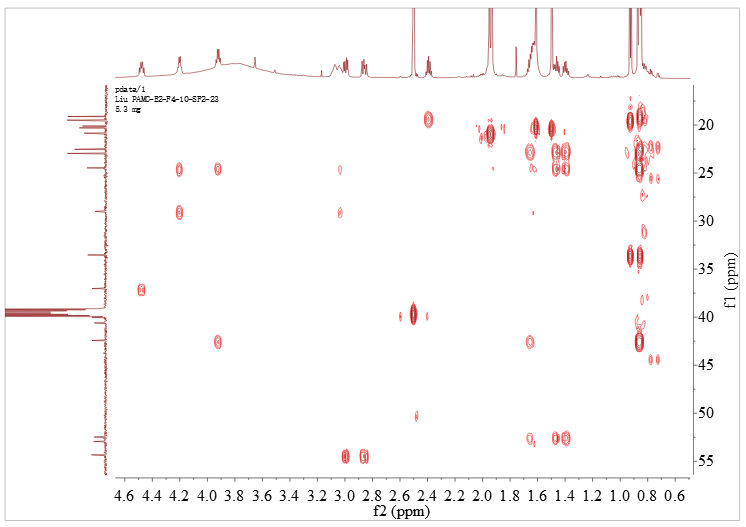


## Figure S13-5-1. HMBC spectrum of 3 with expansion


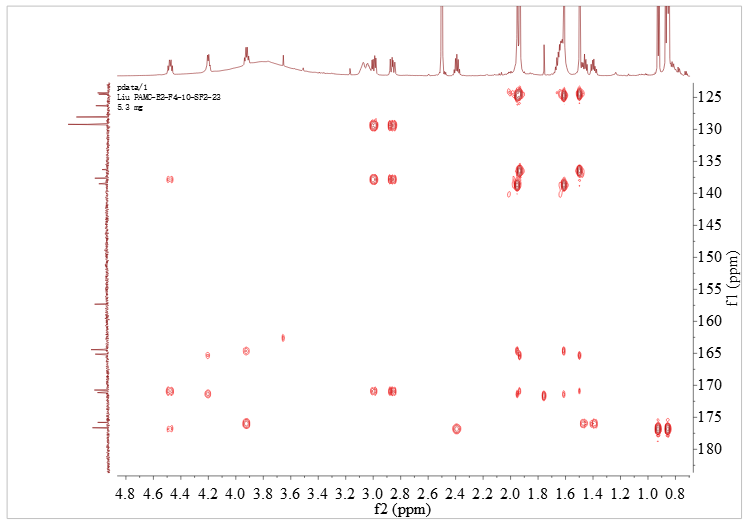


## Figure S13-5-2. HMBC spectrum of 3 with expansion


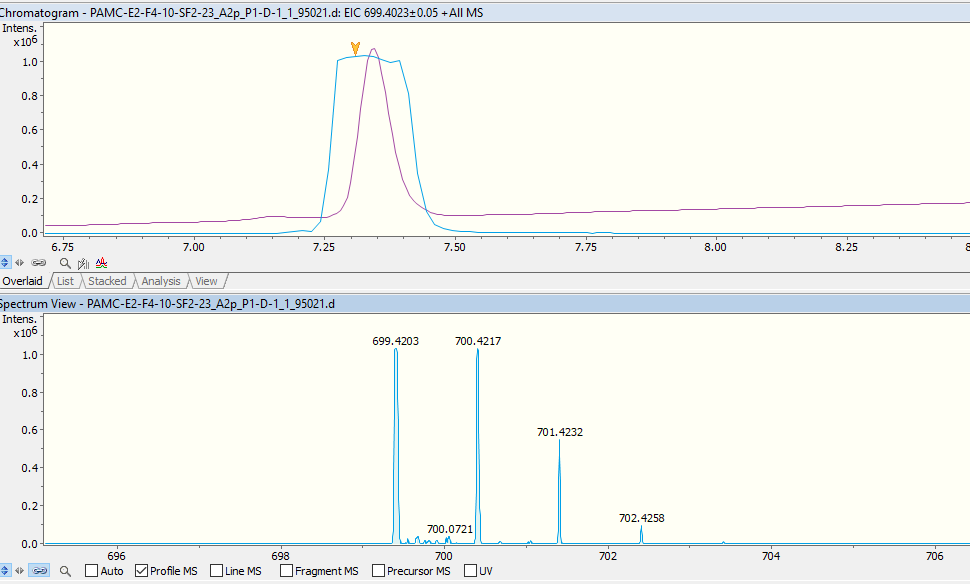


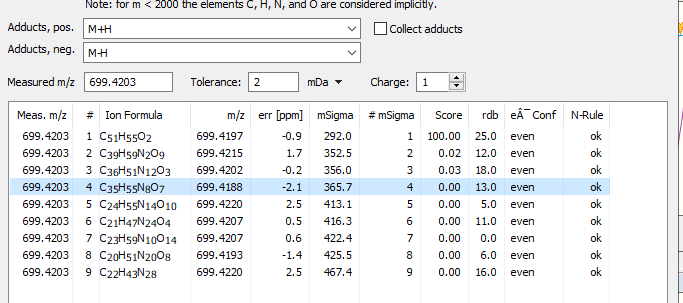


## Figure S13-6. UPLC-HR-ESI-MS spectrum of 3


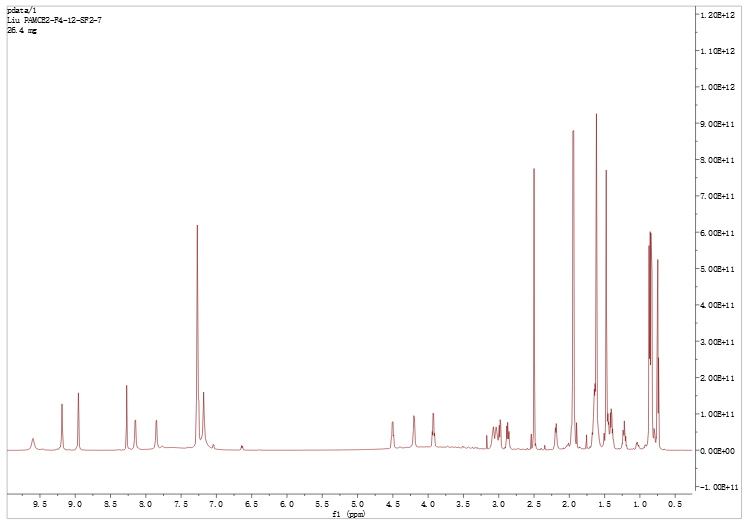


## Figure S14-1. ^1^H NMR spectrum of 4 (DMSO-*d*_6_,700MHz)


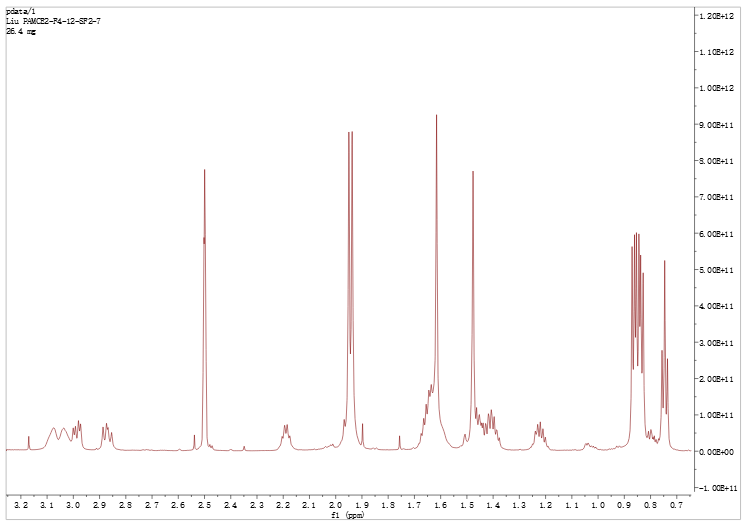


## Figure S14-1-1. ^1^H NMR spectrum of 4 with expansion


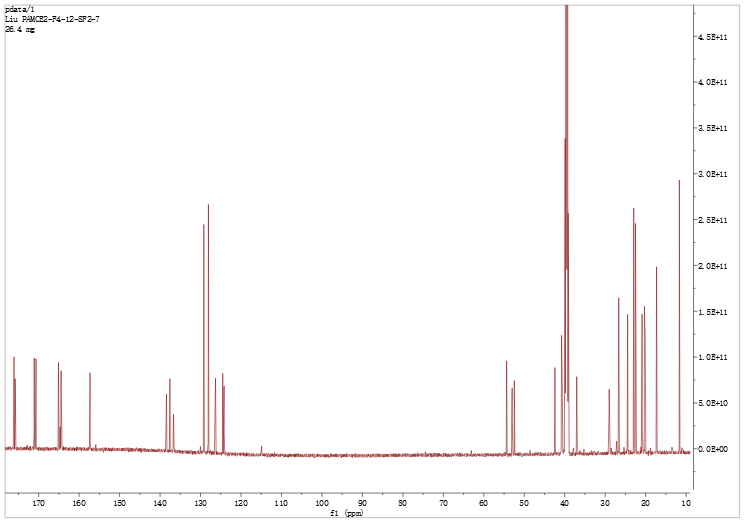


## Figure S14-2. ^13^C NMR spectrum of 4 (DMSO-*d*_6_, 176MHz)


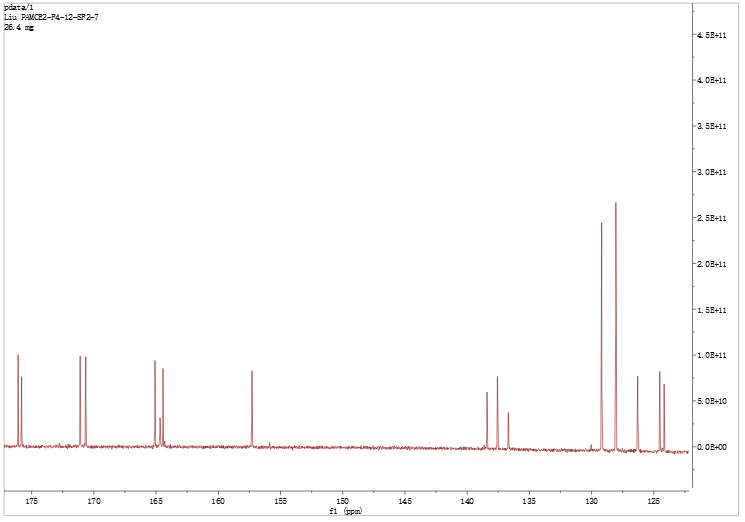


## Figure S14-2-1. ^13^C NMR spectrum of 4 with expansion


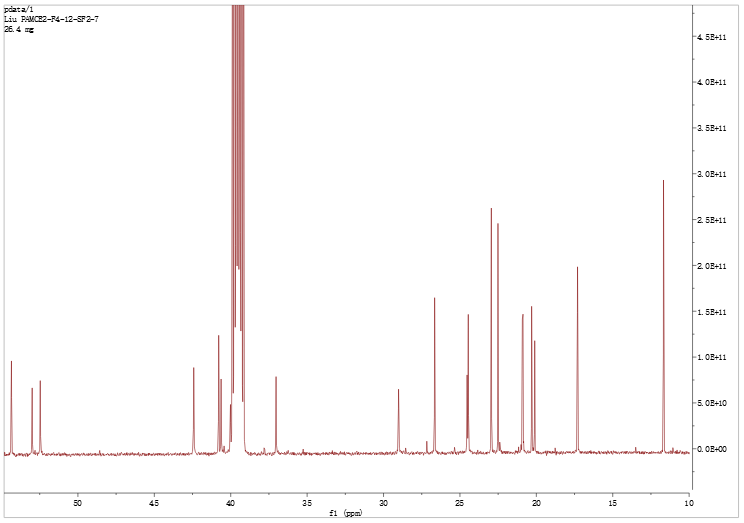


## Figure S14-2-2. ^13^C NMR spectrum of 4 with expansion


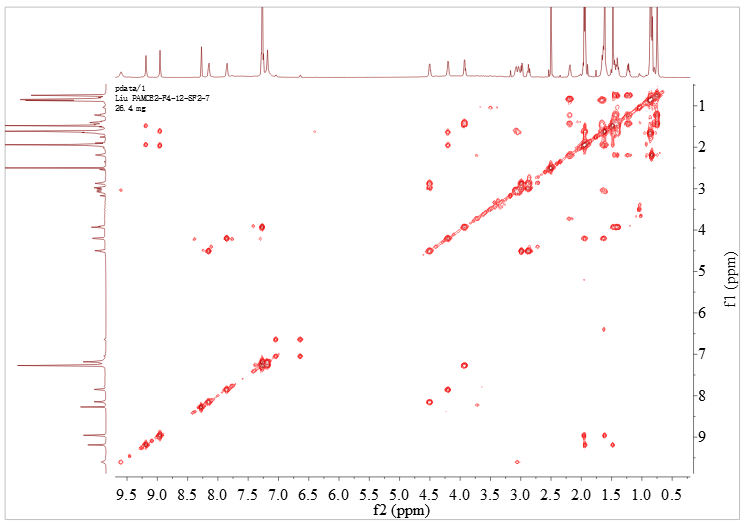


## Figure S14-3. COSY spectrum of 4


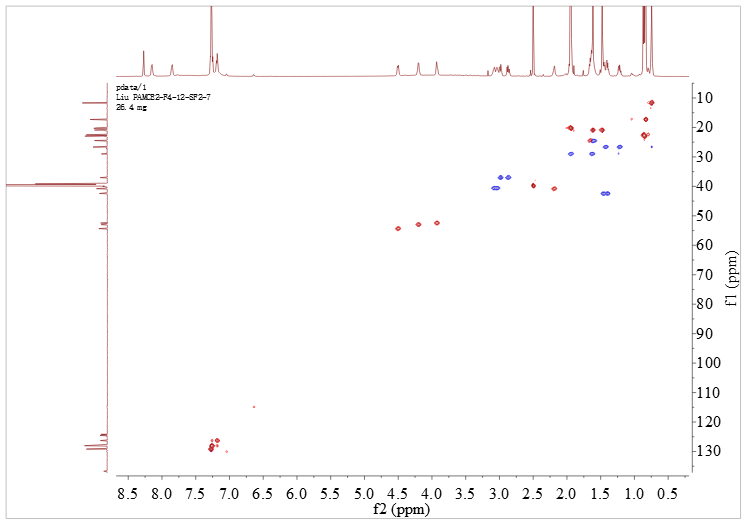


## Figure S14-4. Multiplicity-edited HSQC spectrum of 4


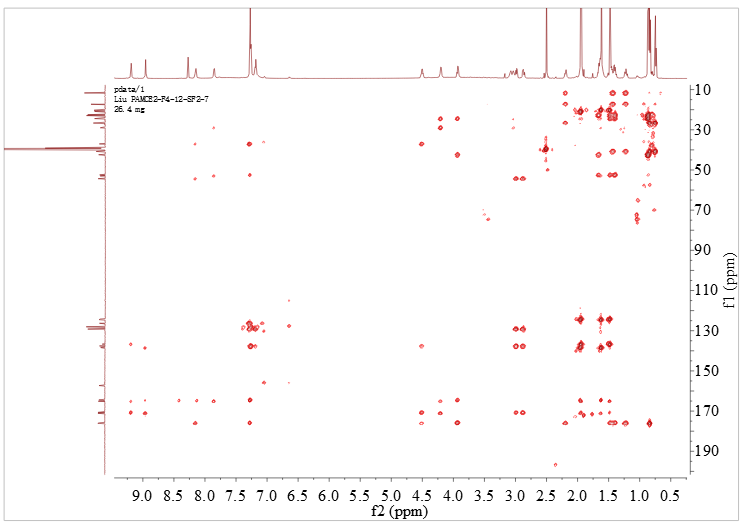


## Figure S14-5. HMBC spectrum of 4


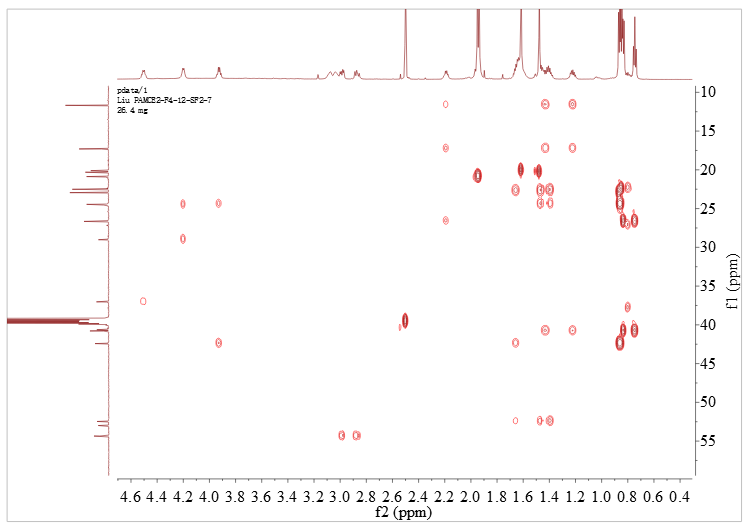


## Figure S14-5-1. HMBC spectrum of 4 with expansion


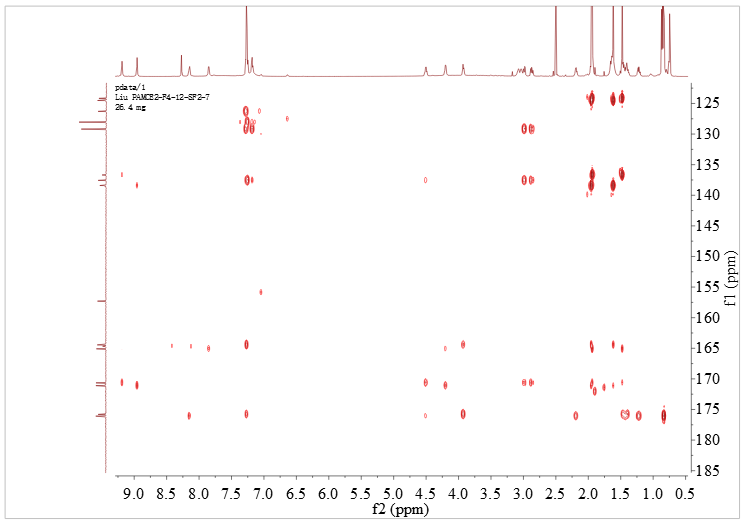


## Figure S14-5-2. HMBC spectrum of 4 with expansion


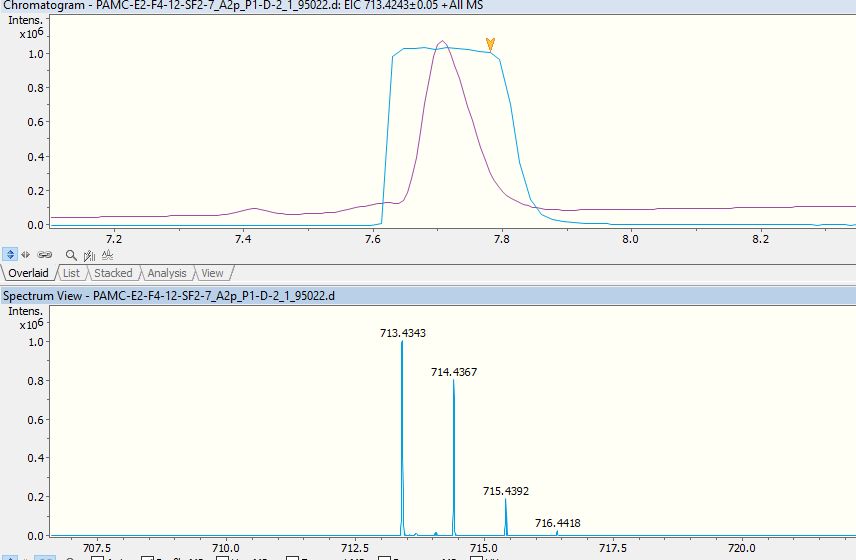


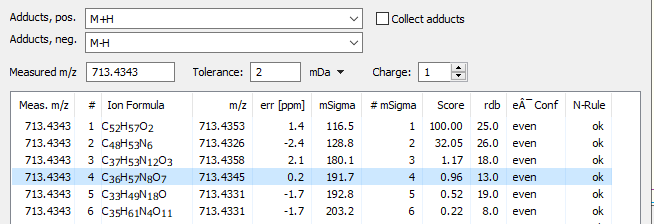


## Figure S14-6. UPLC-HR-ESI-MS spectrum of 4


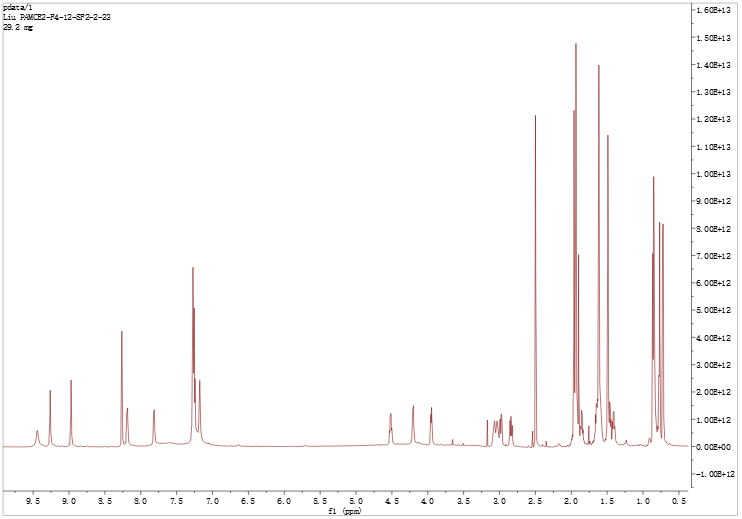


## Figure S15-1. ^1^H NMR spectrum of 5 (DMSO-*d*_6_,700MHz)


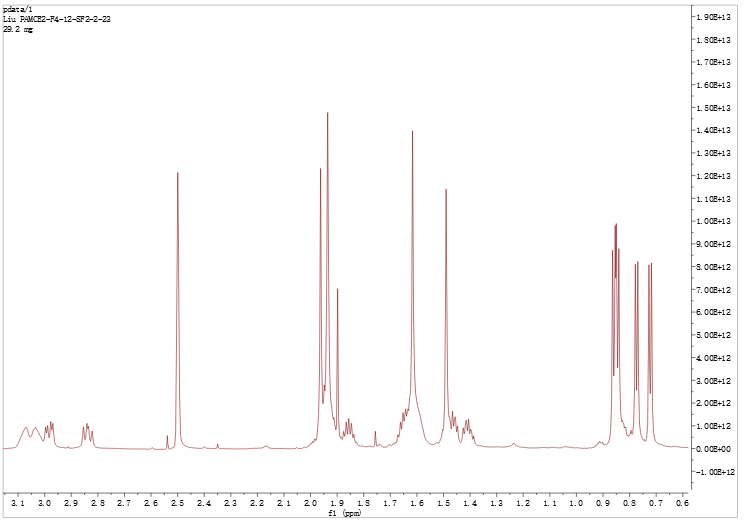


## Figure S15-1-1. ^1^H NMR spectrum of 5 with expansion


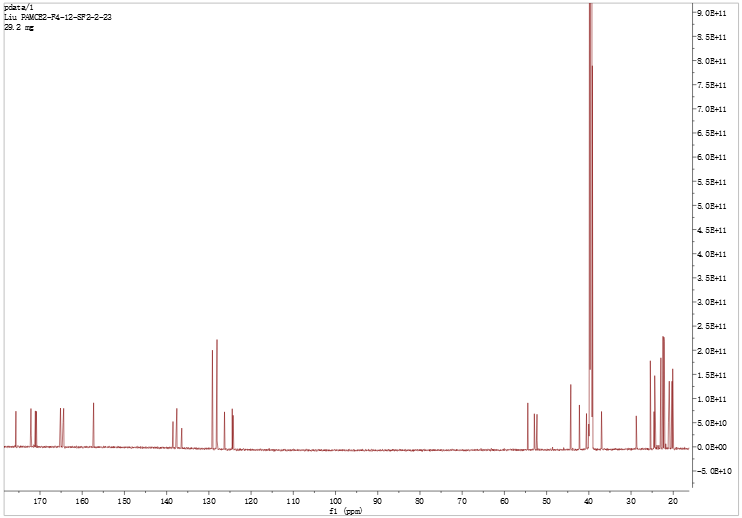


## Figure S15-2. ^13^C NMR spectrum of 5 (DMSO-*d*_6_, 176MHz)


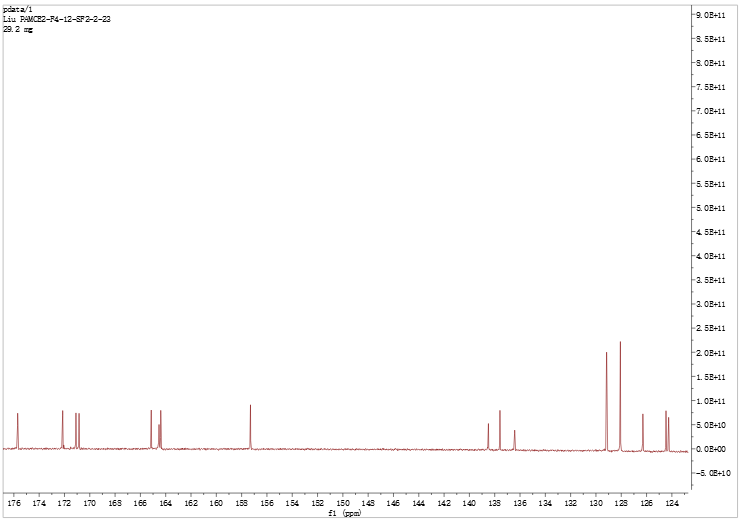


## Figure S15-2-1. ^13^C NMR spectrum of 5 with expansion


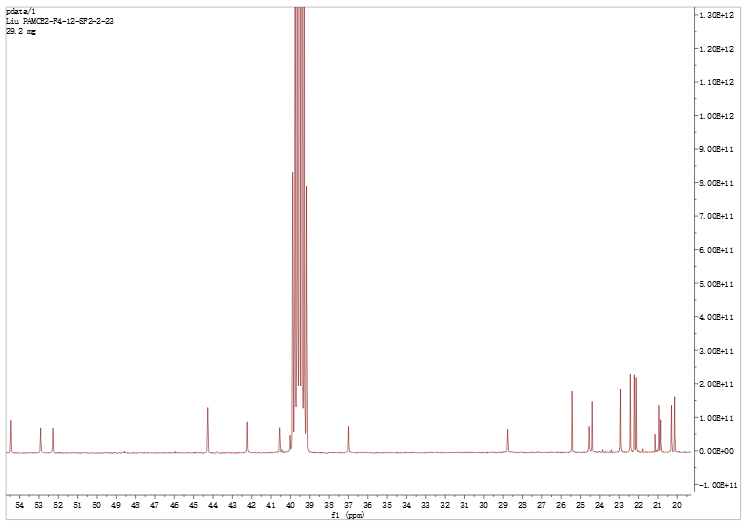


## Figure S15-2-2. ^13^C NMR spectrum of 5 with expansion


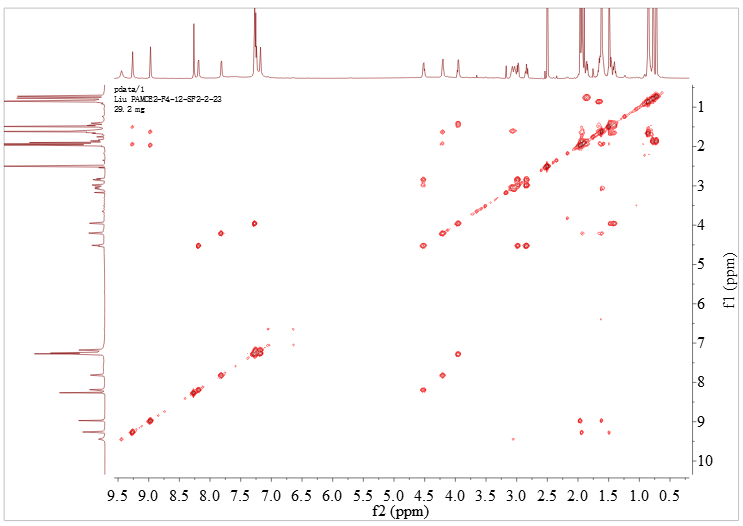


## Figure S15-3. COSY spectrum of 5.


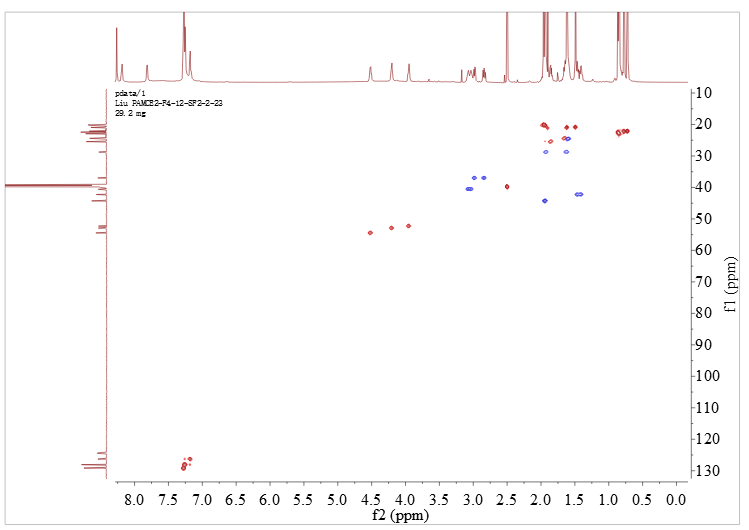


## Figure S15-4. Multiplicity-edited HSQC spectrum of 5


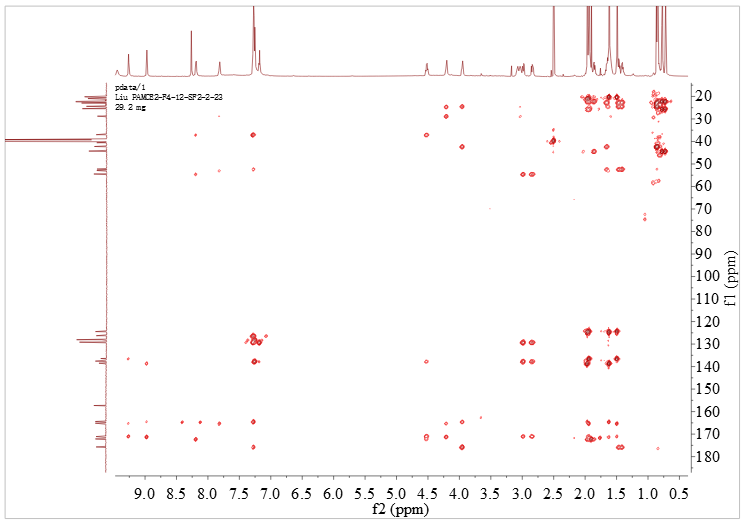


## Figure S15-5. HMBC spectrum of 5


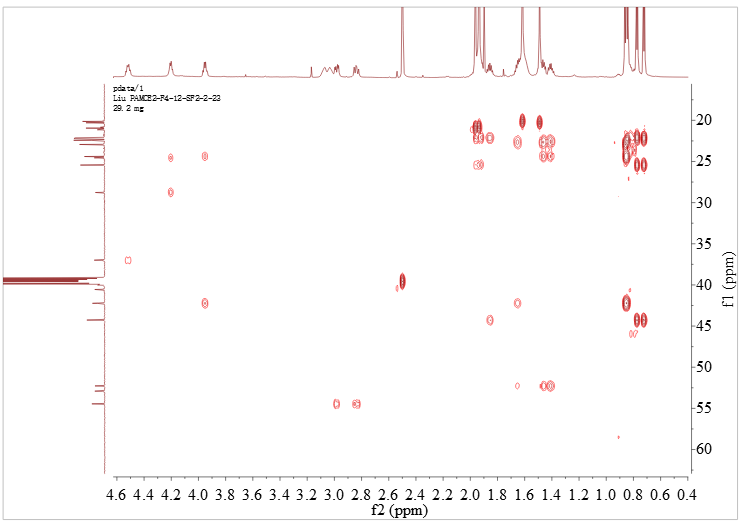


## Figure S15-5-1. HMBC spectrum of 5 with expansion


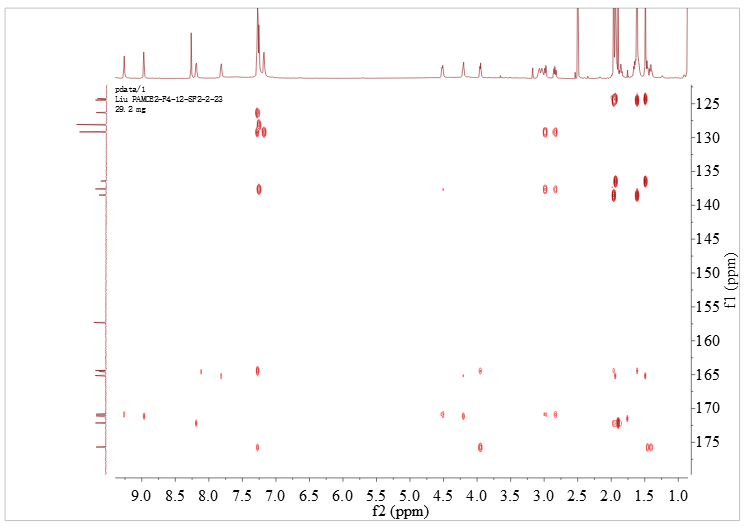


## Figure S15-5-2. HMBC spectrum of 5 with expansion


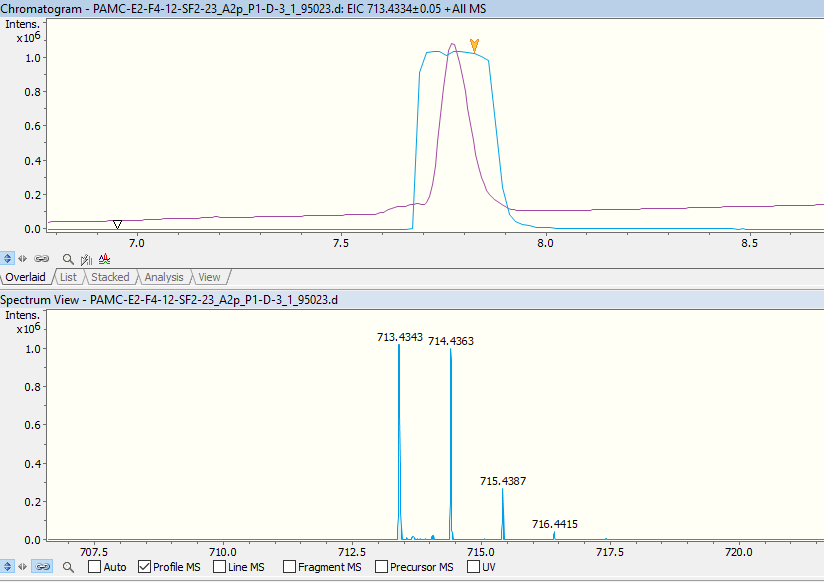


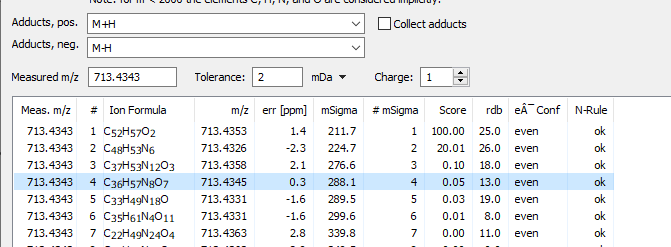


## Figure S15-6. UPLC-HR-ESI-MS spectrum of 5


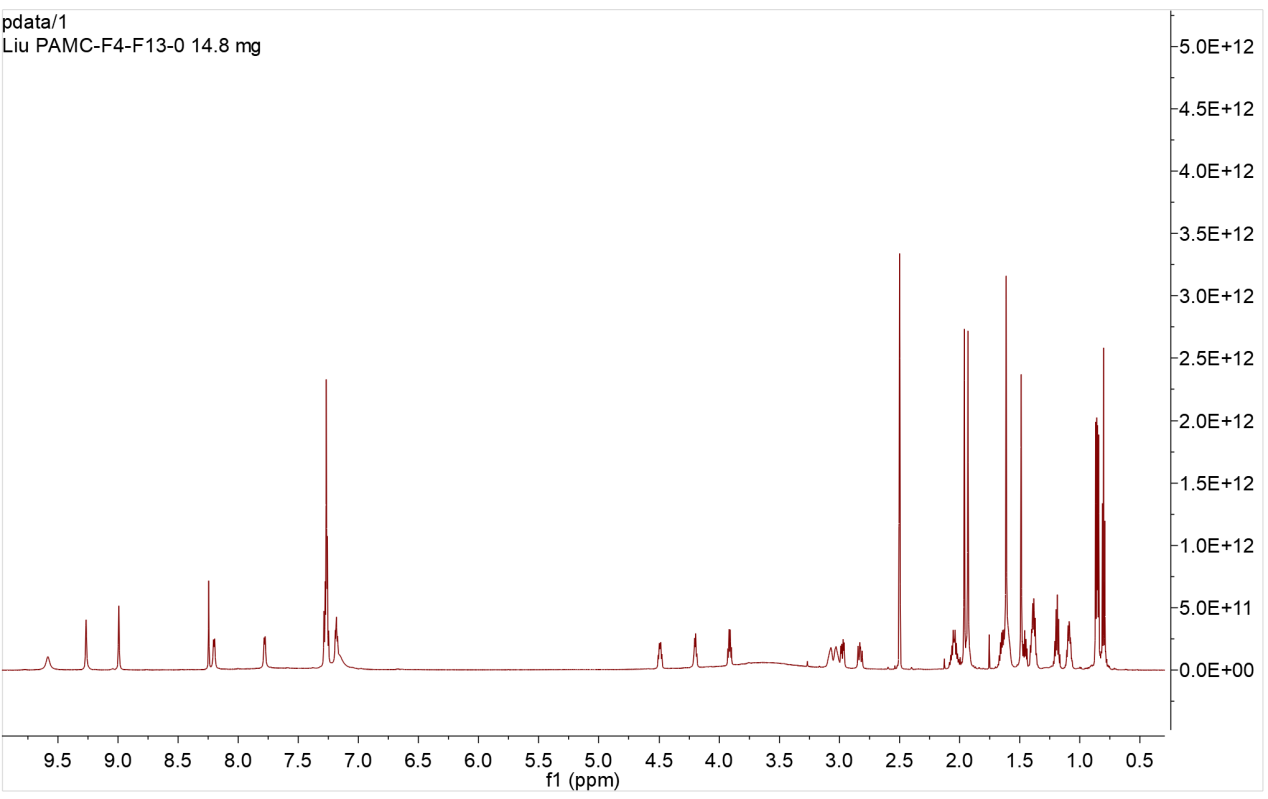


## Figure S16-1. ^1^H NMR spectrum of 6 (DMSO-*d*_6_,700MHz)


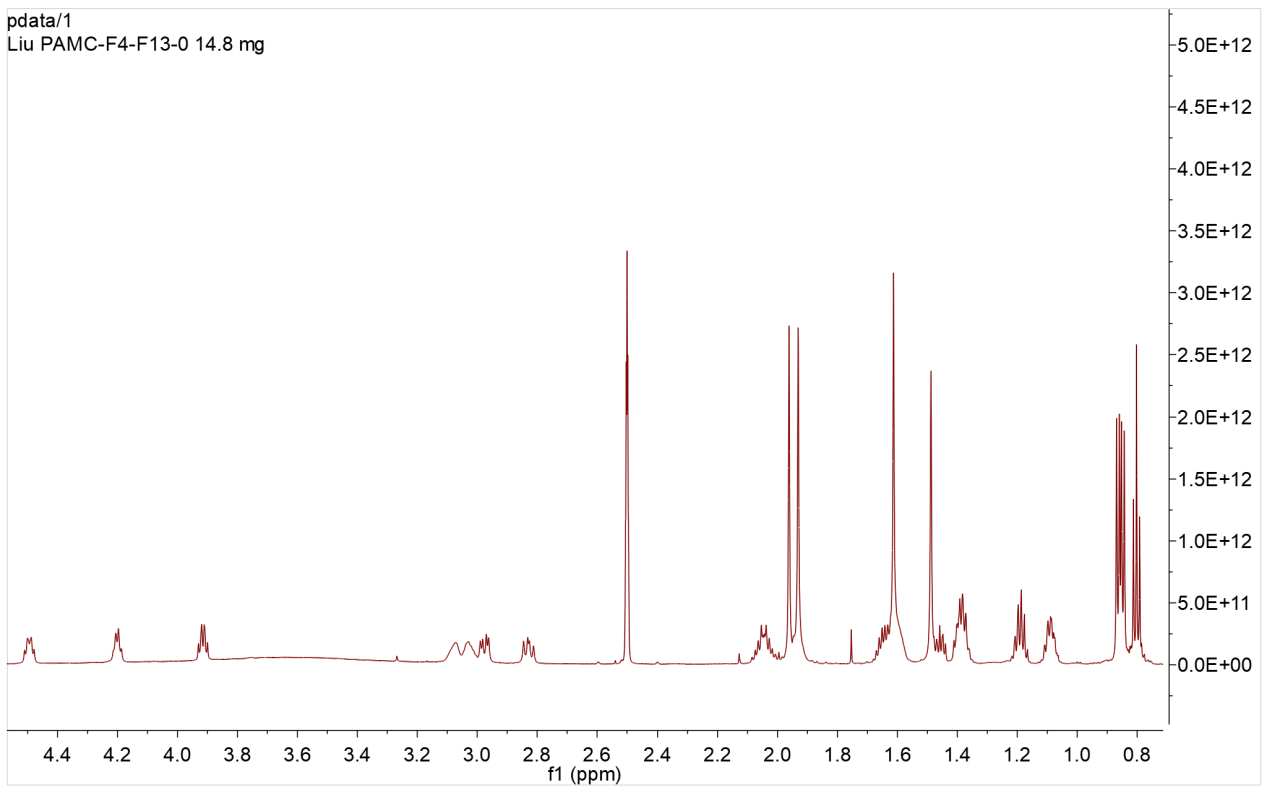


## Figure S16-1-1. ^1^H NMR spectrum of 6 with expansion


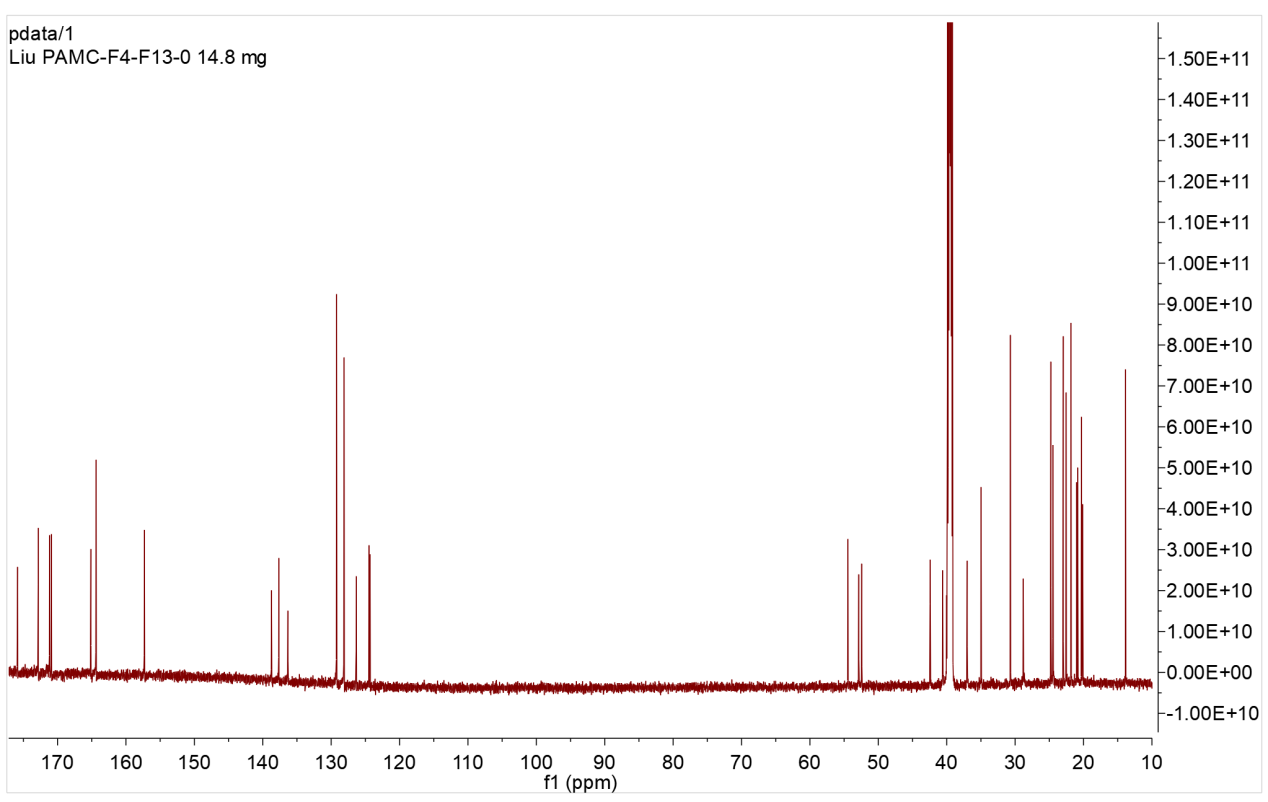


## Figure S16-2. ^13^C NMR spectrum of 6 (DMSO-*d*_6_, 176MHz)


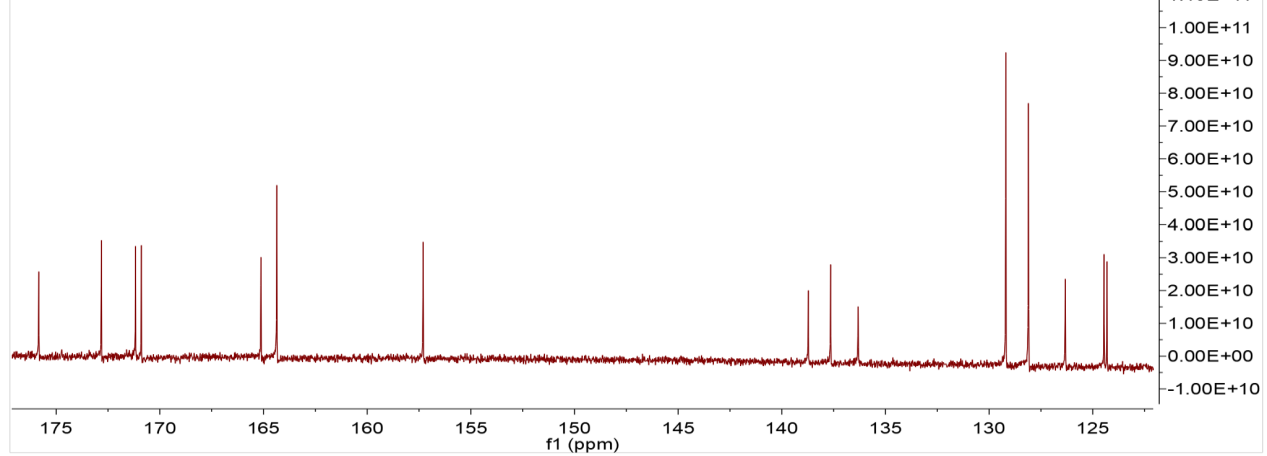


## Figure S16-2-1. ^13^C NMR spectrum of 6 with expansion


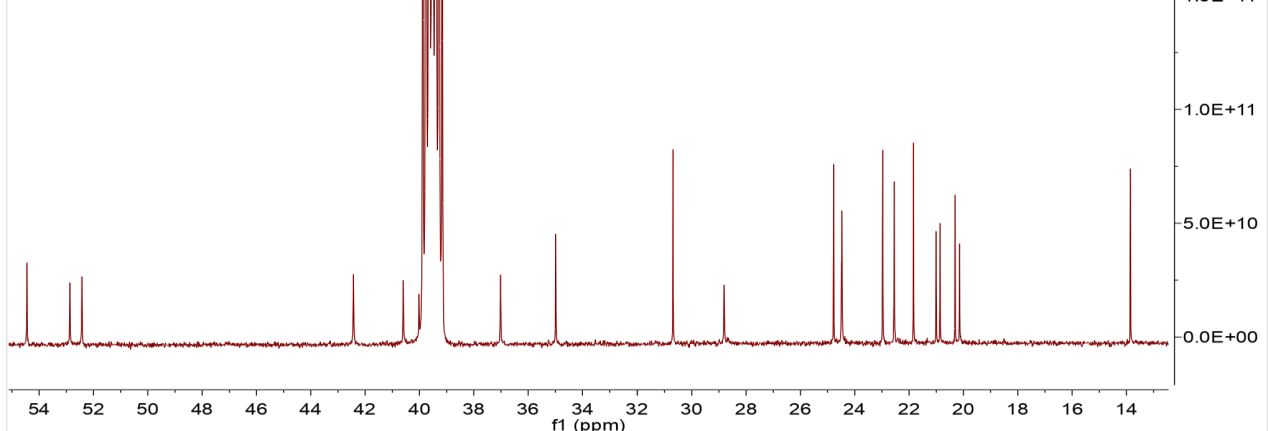


## Figure S16-2-2. ^13^C NMR spectrum of 6 with expansion


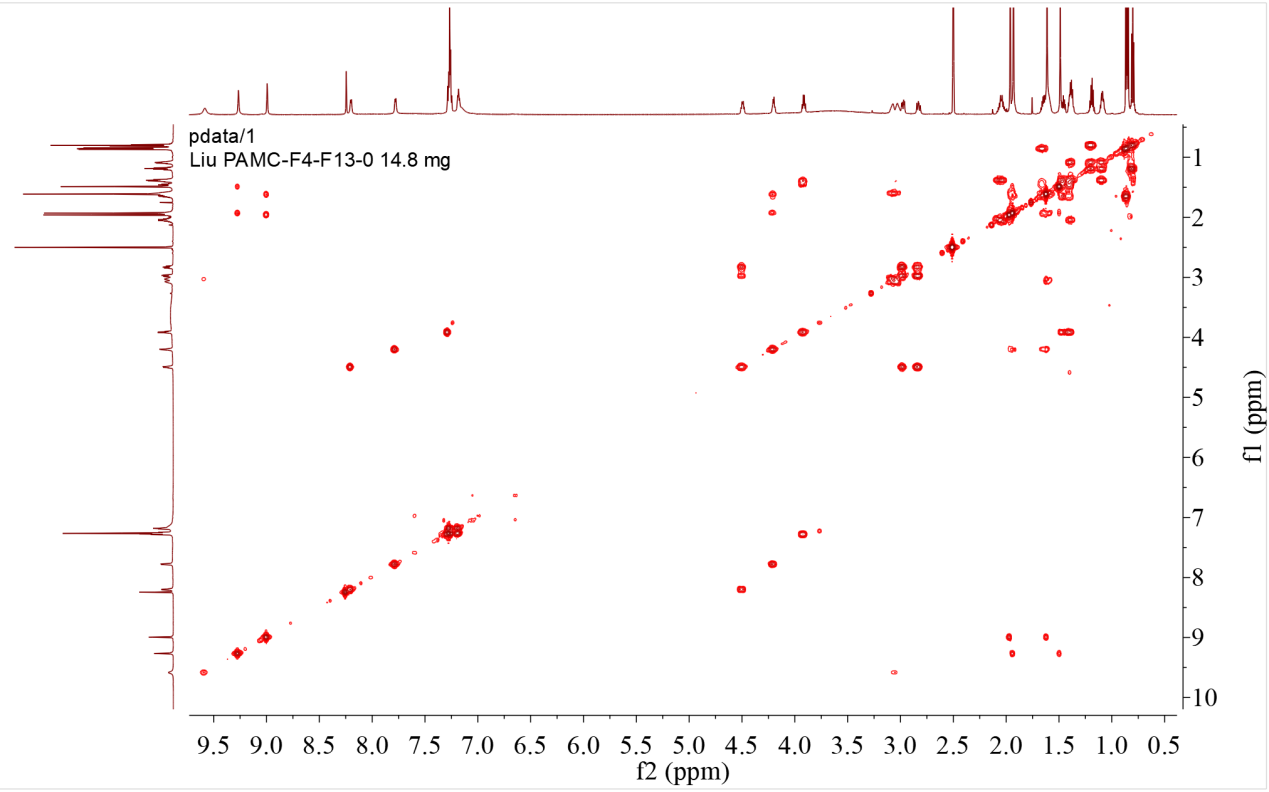


## Figure S16-3. COSY spectrum of 6


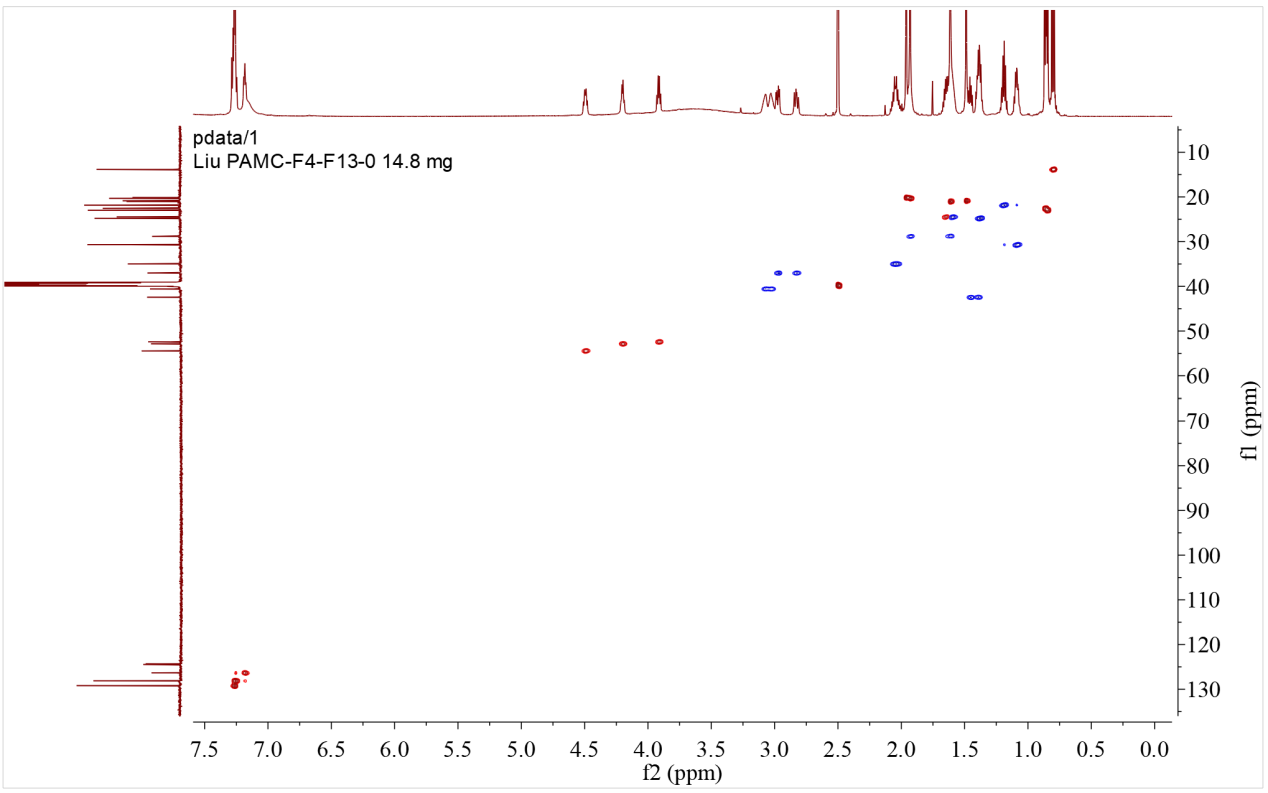


## Figure S16-4. Multiplicity-edited HSQC spectrum of 6


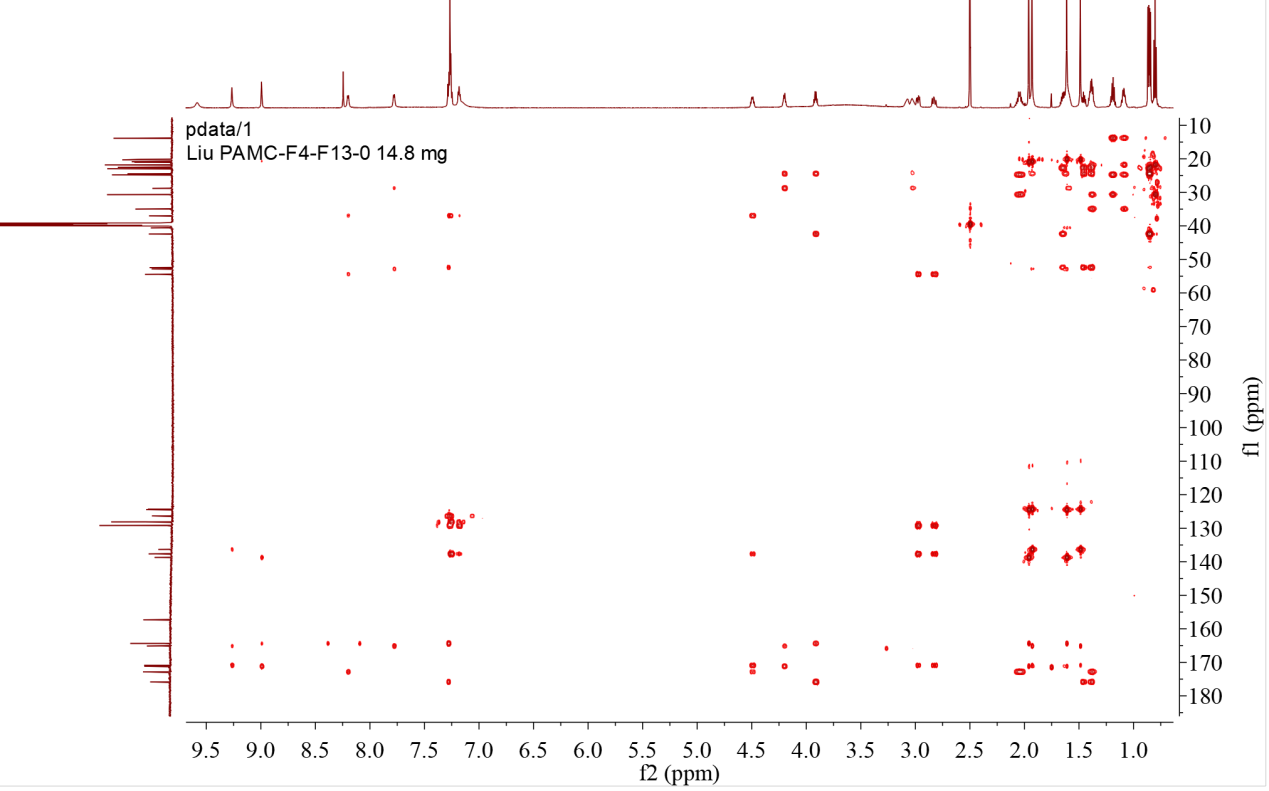


## Figure S16-5. HMBC spectrum of 6


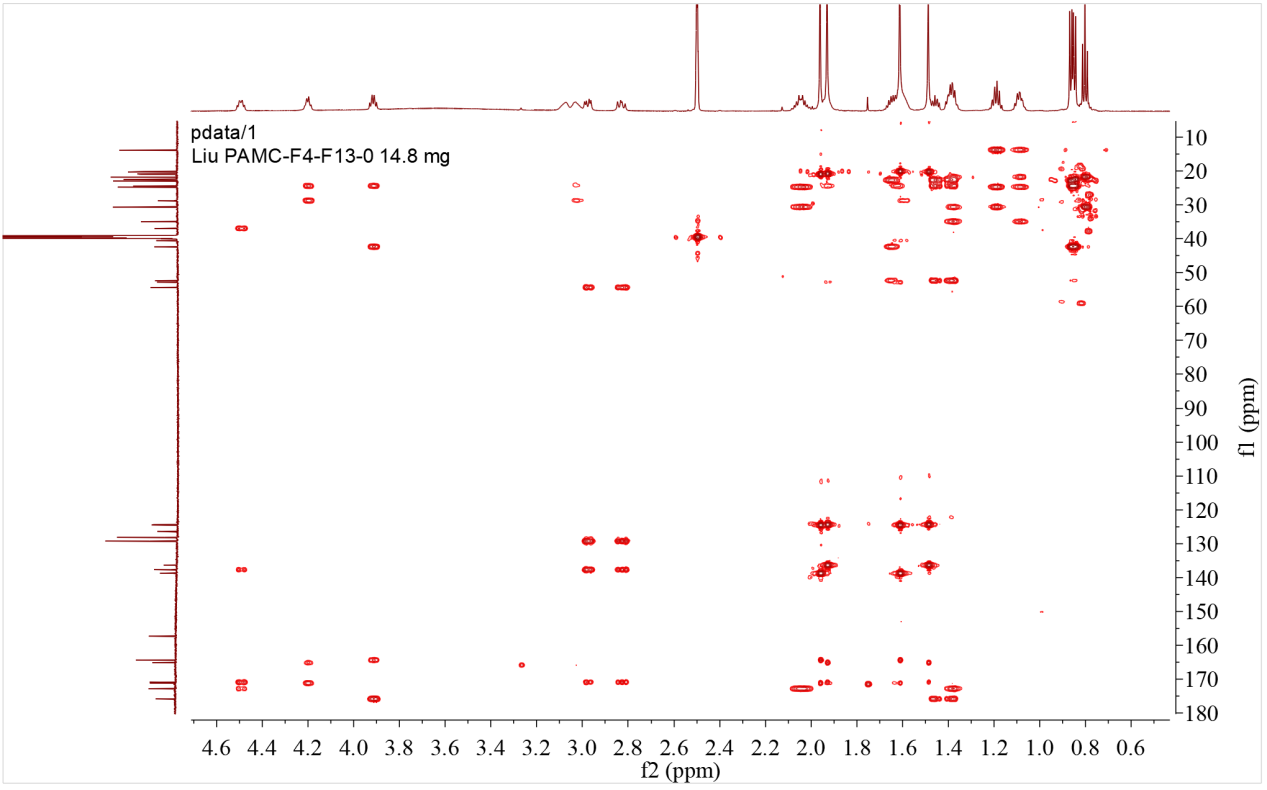


## Figure S16-5-1. HMBC spectrum of 6 with expansion


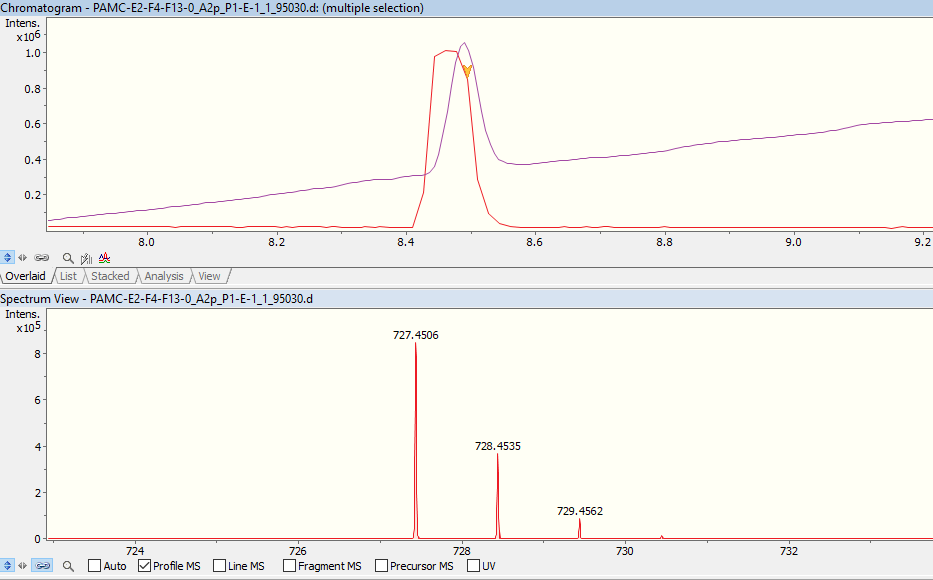


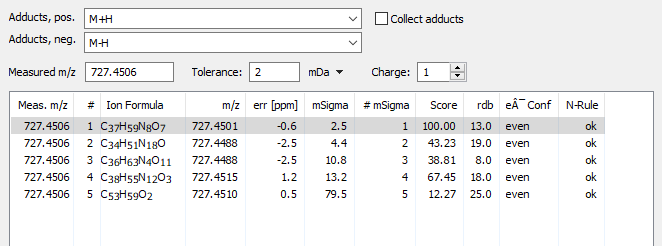


## Figure S16-6. UPLC-HR-ESI-MS spectrum of 6


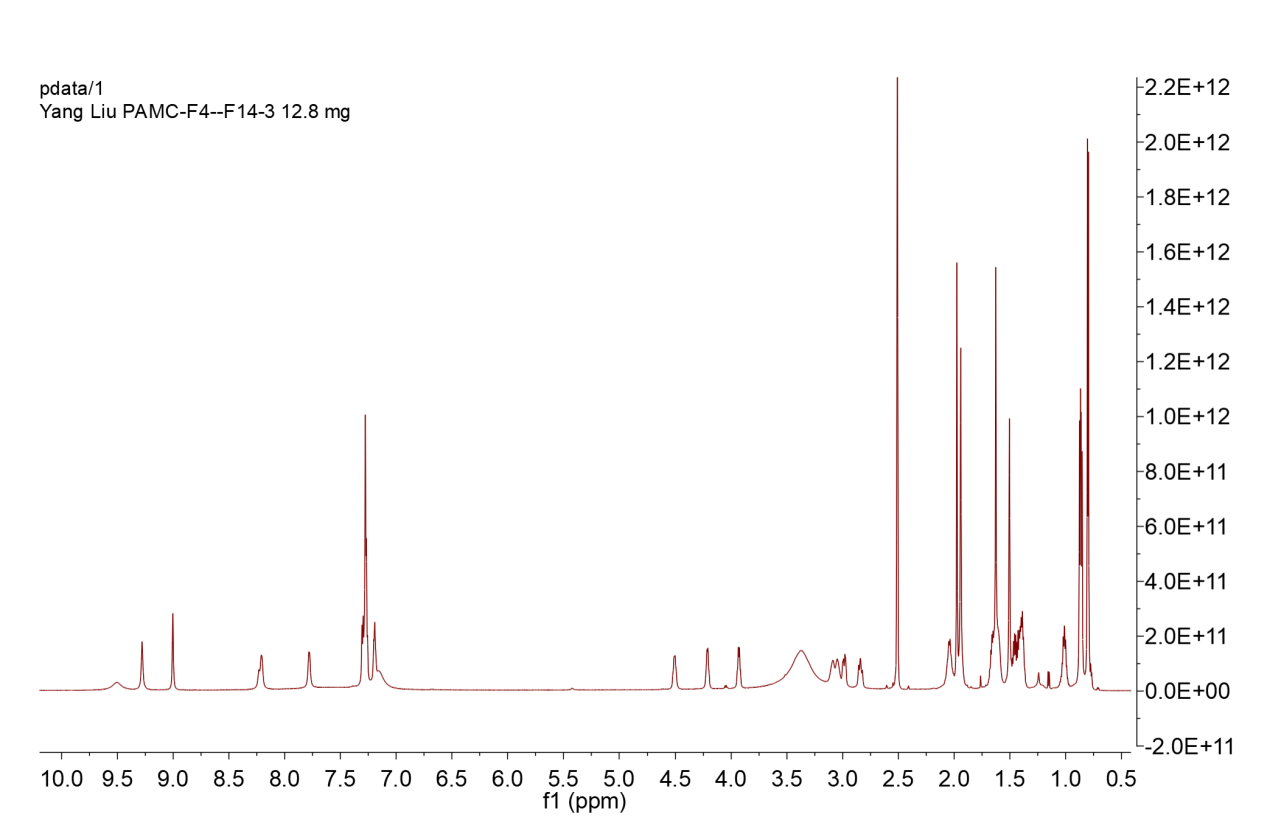


## Figure S17-1. ^1^H NMR spectrum of 7 (DMSO-*d*_6_,700MHz)


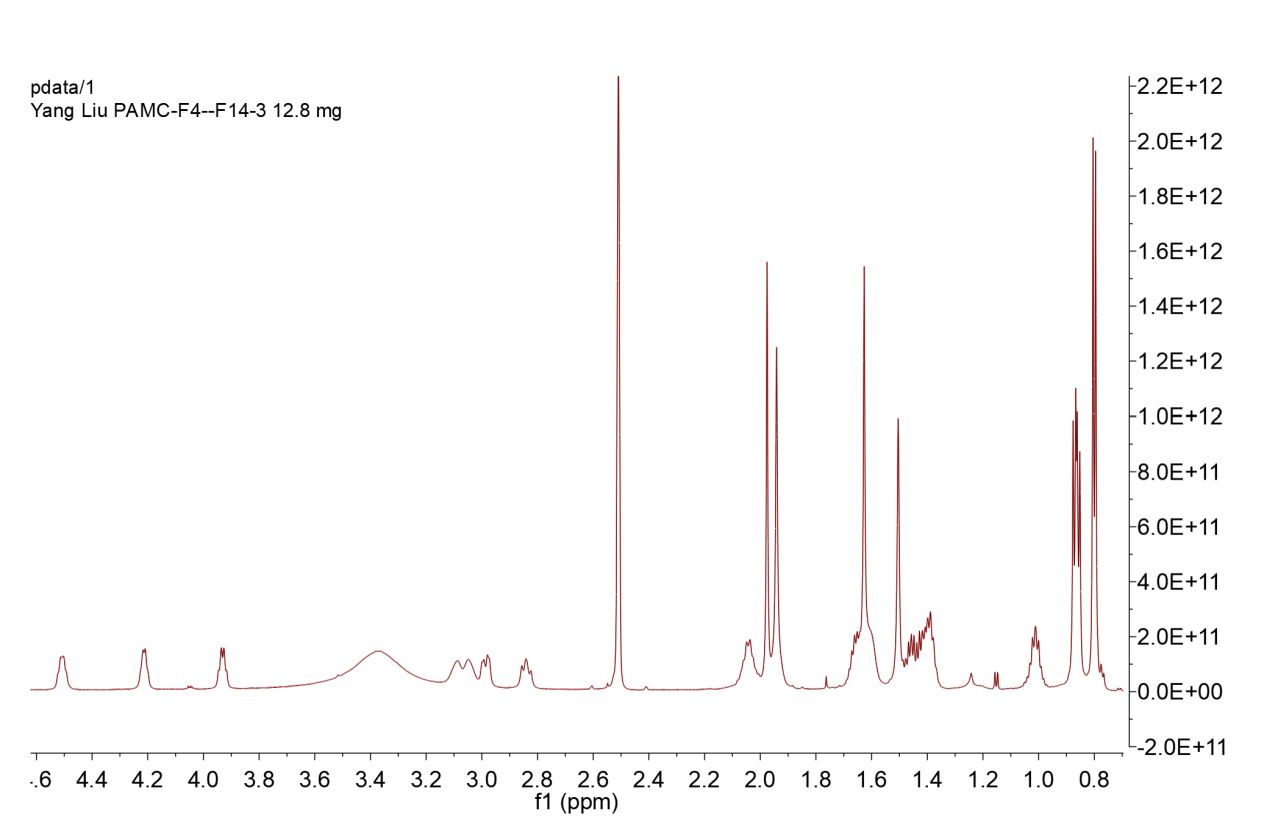


## Figure S17-1-1. ^1^H NMR spectrum of 7 with expansion


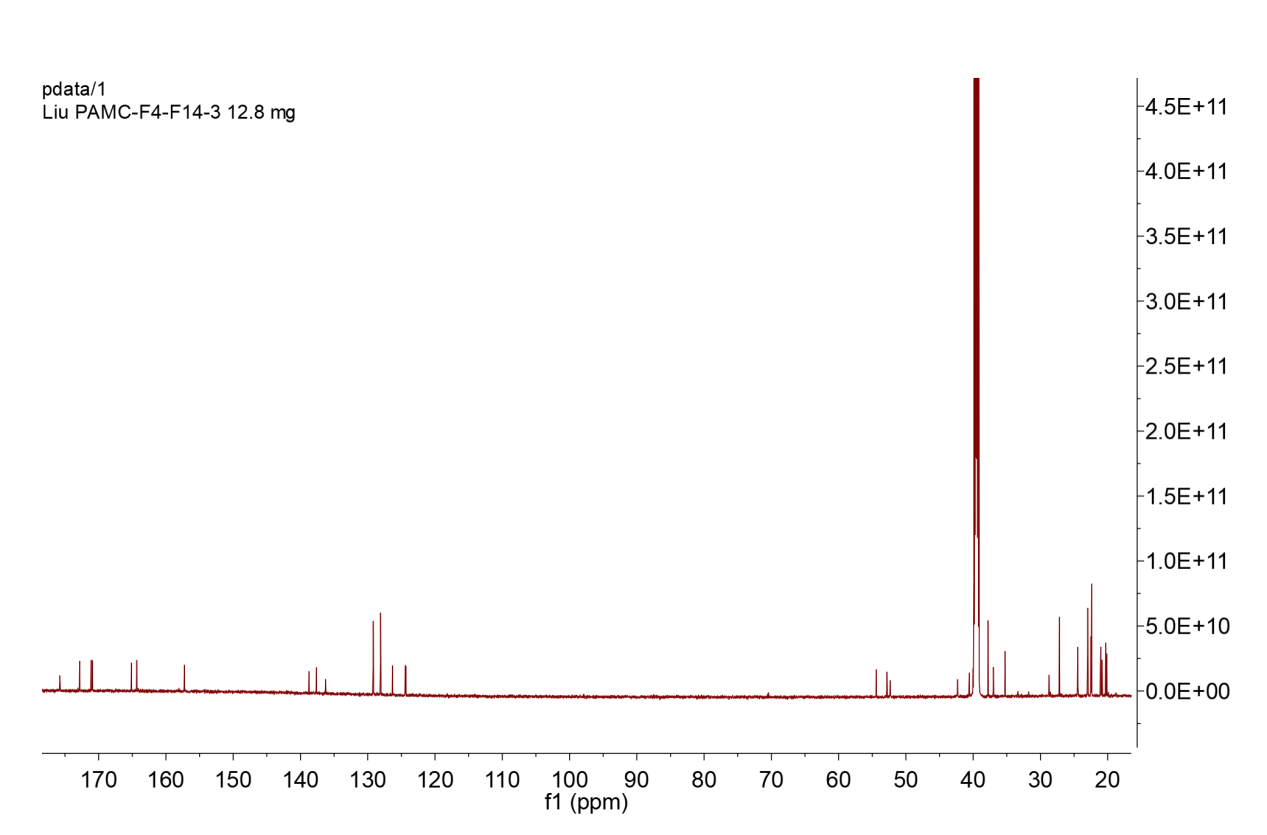


## Figure S17-2. ^13^C NMR spectrum of 7 (DMSO-*d*_6_, 176MHz)


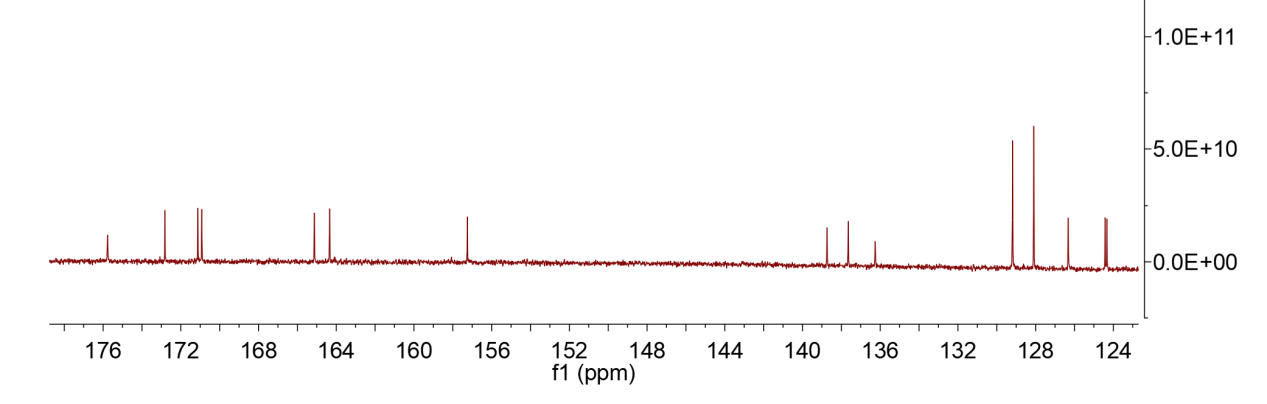


## Figure S17-2-1. ^13^C NMR spectrum of 7 with expansion


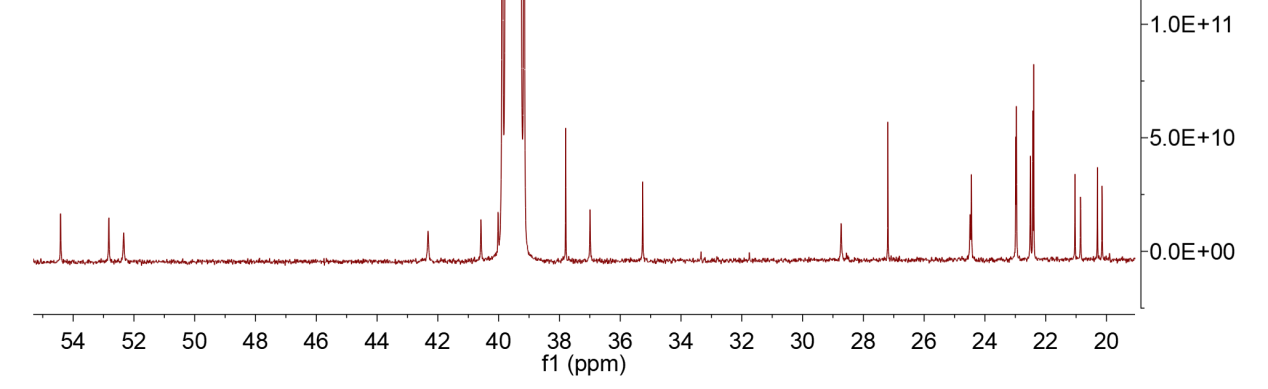


## Figure S17-2-2. ^13^C NMR spectrum of 7 with expansion


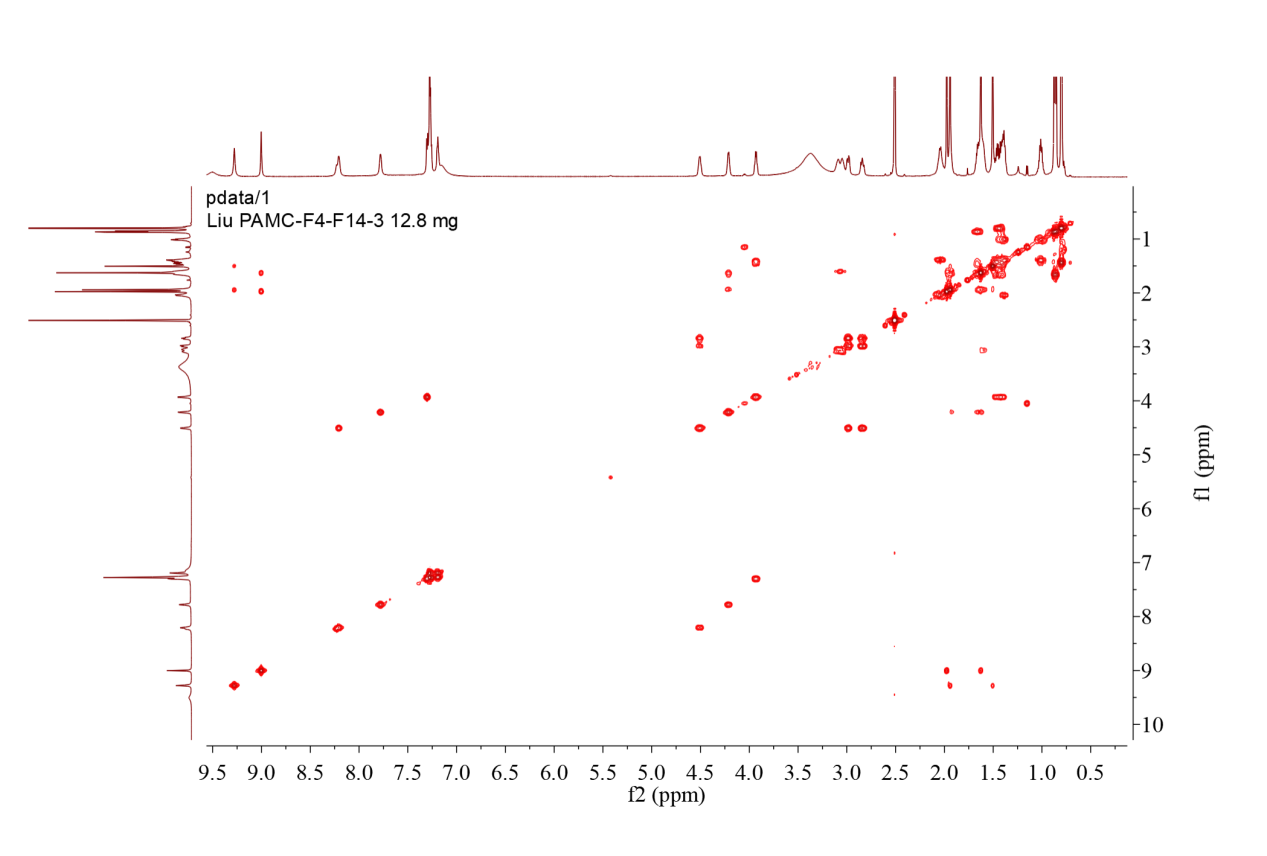


## Figure S17-3. COSY spectrum of 7


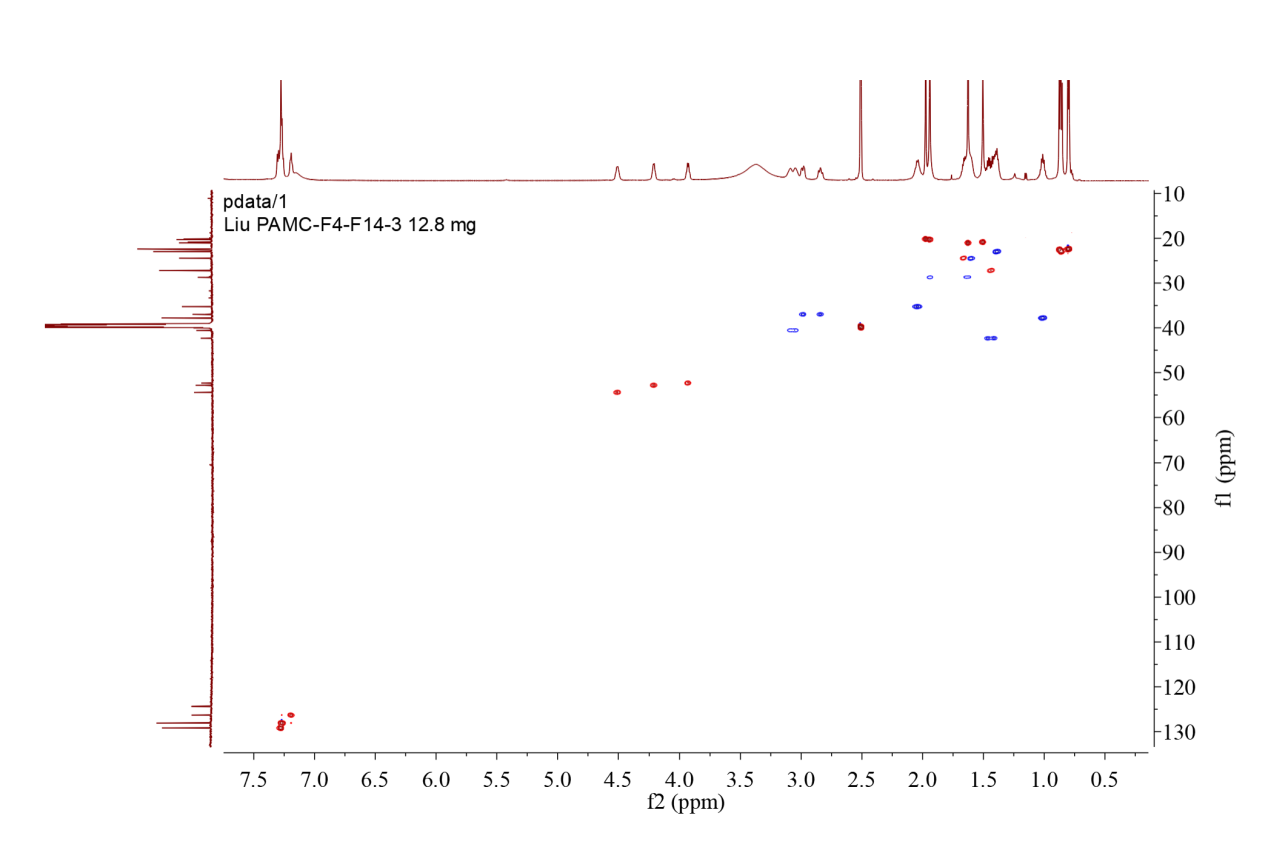


## Figure S17-4. Multiplicity-edited HSQC spectrum of 7


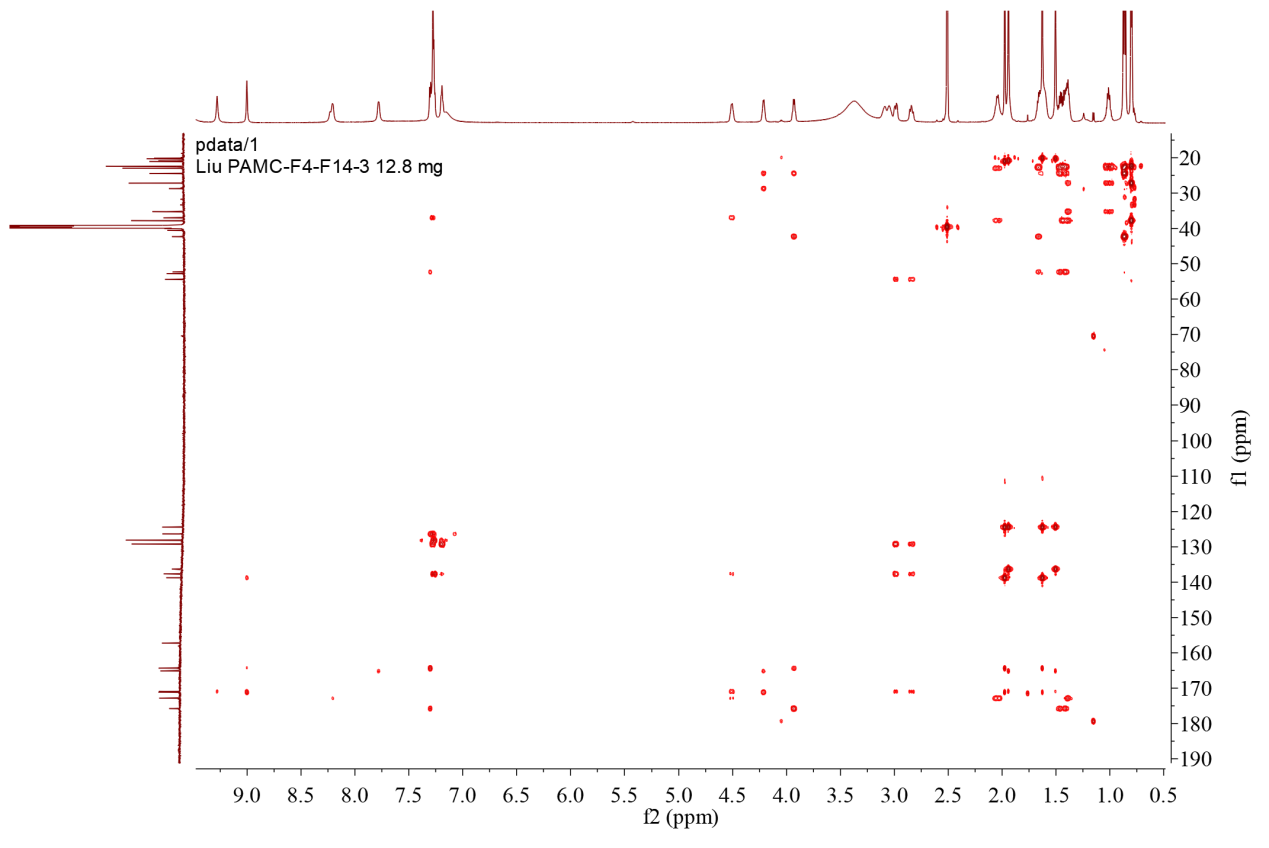


## Figure S17-5. HMBC spectrum of 7


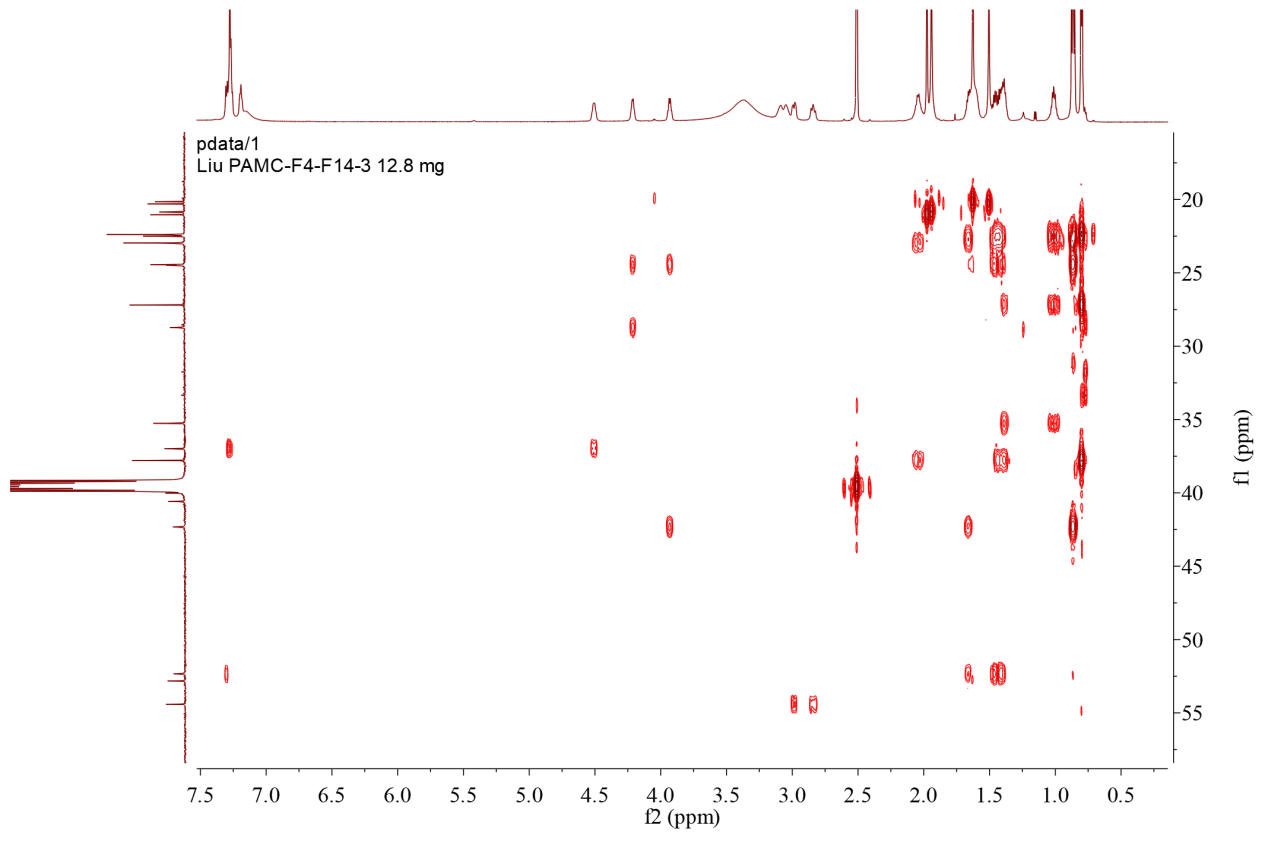


## Figure S17-5-1. HMBC spectrum of 7 with expansion


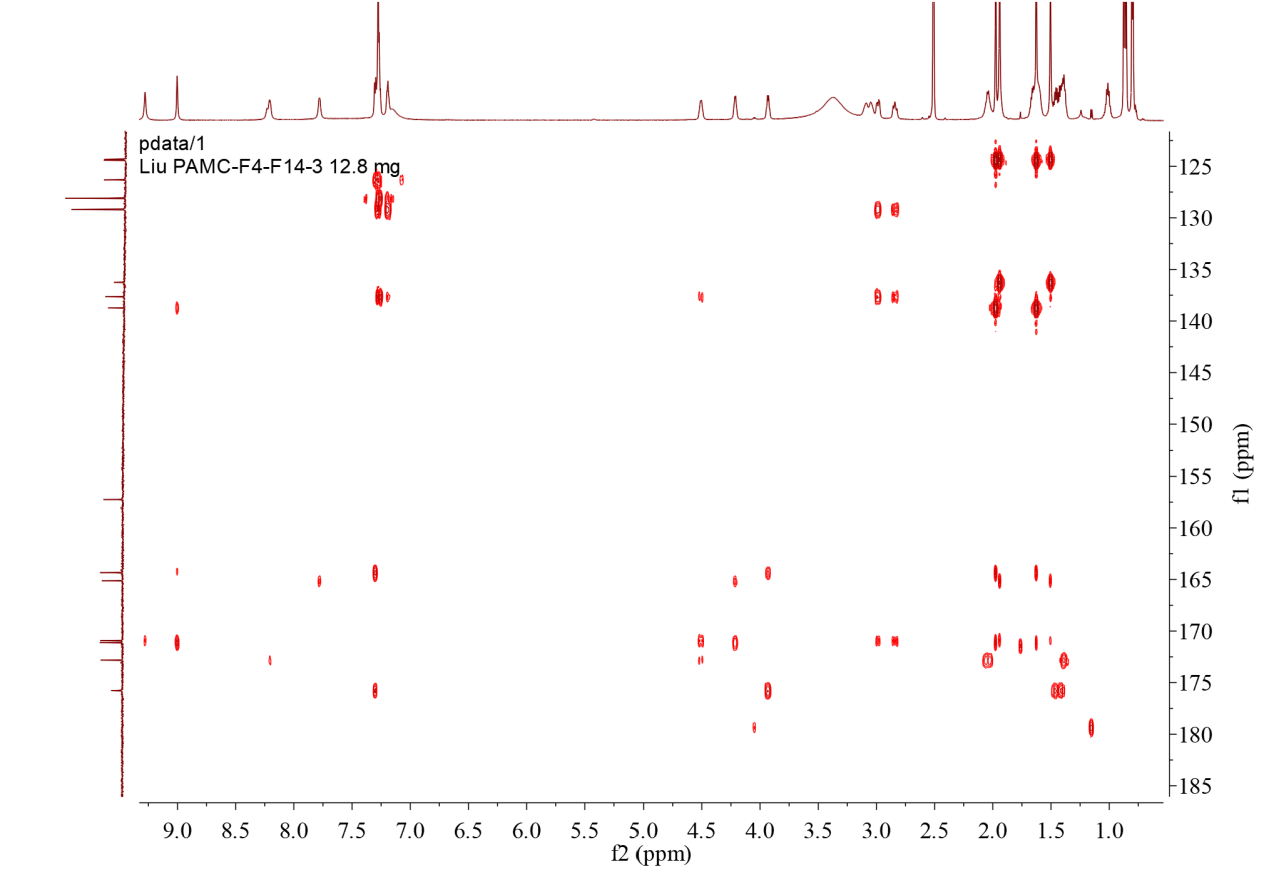


## Figure S17-5-2. HMBC spectrum of 7 with expansion


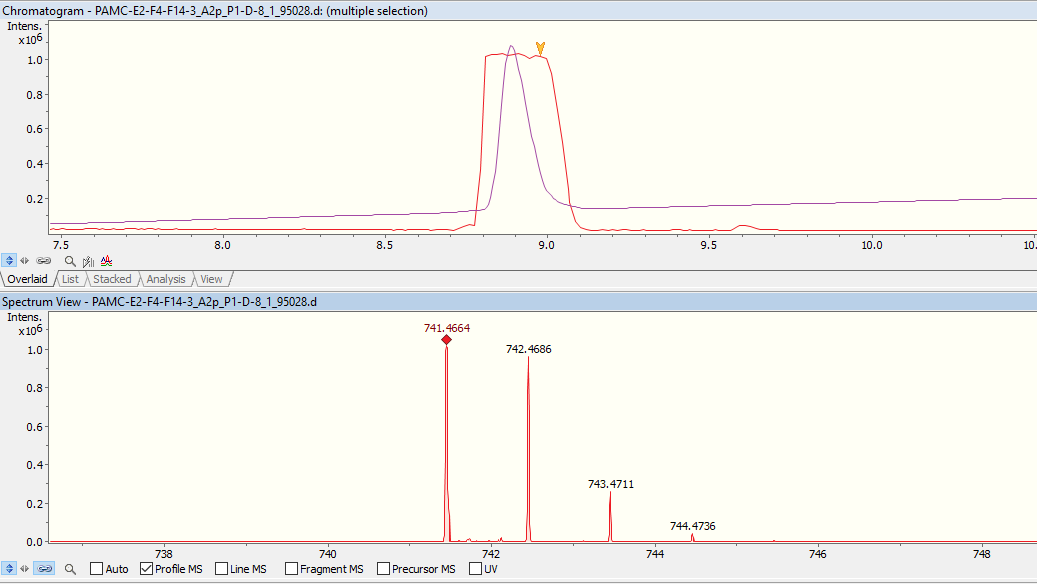


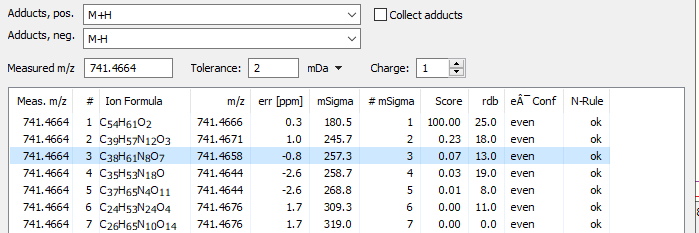


## Figure S17-6. UPLC-HR-ESI-MS spectrum of 7

## Figure S18-1. ^1^H NMR spectrum of 8 (DMSO-*d*_6_,700MHz)

## Figure S18-1-1. ^1^H NMR spectrum of 8 with expansion

## Figure S18-2. ^13^C NMR spectrum of 8 (DMSO-*d*_6_, 176MHz)

## Figure S18-2-1. ^13^C NMR spectrum of 8 with expansion

## Figure S18-2-2. ^13^C NMR spectrum of 8 with expansion

## Figure S18-3. COSY spectrum of 8.

## Figure S18-4. Multiplicity-edited HSQC spectrum of 8

## Figure S18-5. HMBC spectrum of 8

## Figure S18-5-1. HMBC spectrum of 8 with expansion

## Figure S18-5-2. HMBC spectrum of 8 with expansion

## Figure S18-6. UPLC-HR-ESI-MS spectrum of 8

## Figure S19-1. ^1^H NMR spectrum of 9 (DMSO-*d*_6_,700MHz)

## Figure S19-1-1. ^1^H NMR spectrum of 9 with expansion

## Figure S19-2. ^13^C NMR spectrum of 9 (DMSO-*d*_6_, 176MHz)

## Figure S19-2-1. ^13^C NMR spectrum of 9 with expansion

## Figure S19-2-2. ^13^C NMR spectrum of 9 with expansion

## Figure S19-3. COSY spectrum of 9

## Figure S19-4. Multiplicity-edited HSQC spectrum of 9

## Figure S19-5. HMBC spectrum of 9

## Figure S19-5-1. HMBC spectrum of 9 with expansion

## Figure S19-5-2. HMBC spectrum of 9 with expansion

## Figure S19-6. UPLC-HR-ESI-MS spectrum of 9

## Figure S20-1. ^1^H NMR spectrum of 10 (DMSO-*d*_6_,700MHz)

## Figure S20-1-1. ^1^H NMR spectrum of 10 with expansion

## Figure S20-2. ^13^C NMR spectrum of 10 (DMSO-*d*_6_, 176MHz)

## Figure S20-2-1. ^13^C NMR spectrum of 10 with expansion

## Figure S20-2-2. ^13^C NMR spectrum of 10 with expansion

## Figure S20-3. COSY spectrum of 10

## Figure S20-4. Multiplicity-edited HSQC spectrum of 10

## Figure S20-5. HMBC spectrum of 10

## Figure S20-5-1. HMBC spectrum of 10 with expansion

## Figure S20-5-2. HMBC spectrum of 10 with expansion

## Figure S20-6. UPLC-HR-ESI-MS spectrum of 10

## Figure S21-1. ^1^H NMR spectrum of 11 (DMSO-*d*_6_,700MHz)

## Figure S21-1-1. ^1^H NMR spectrum of 11 with expansion

## Figure S21-2. ^13^C NMR spectrum of 11 (DMSO-*d*_6_, 176MHz)

## Figure S21-2-1. ^13^C NMR spectrum of 11 with expansion

## Figure S21-2-2. ^13^C NMR spectrum of 11 with expansion

## Figure S21-3. COSY spectrum of 11.

## Figure S21-4. Multiplicity-edited HSQC spectrum of 11

## Figure S21-5. HMBC spectrum of 11

## Figure S21-5-1. HMBC spectrum of 11 with expansion

## Figure S21-5-2. HMBC spectrum of 11 with expansion

## Figure S21-6. UPLC-HR-ESI-MS spectrum of 11

## Figure S21-7. MS/MS fragmentation pattern of 11

## Figure S22-1. ^1^H NMR spectrum of 12 (DMSO-*d*_6_,700MHz)

## Figure S22-1-1. ^1^H NMR spectrum of 12 with expansion

## Figure S22-2. ^13^C NMR spectrum of 12 (DMSO-*d*_6_, 176MHz)

## Figure S22-2-1. ^13^C NMR spectrum of 12 with expansion

## Figure S22-2-2. ^13^C NMR spectrum of 12 with expansion

## Figure S22-3. COSY spectrum of 12

## Figure S22-4. Multiplicity-edited HSQC spectrum of 12

## Figure S22-5. HMBC spectrum of 12

## Figure S22-5-1. HMBC spectrum of 12 with expansion

## Figure S22-5-2. HMBC spectrum of 12 with expansion

## Figure S22-6. UPLC-HR-ESI-MS spectrum of 12

## Figure S22-7. MS/MS fragmentation pattern of 12

## Figure S23-1. ^1^H NMR spectrum of 13 (DMSO-*d*_6_,700MHz)

## Figure S23-1-1. ^1^H NMR spectrum of 13 with expansion

## Figure S23-2. ^13^C NMR spectrum of 13 (DMSO-*d*_6_, 176MHz)

## Figure S23-2-1. ^13^C NMR spectrum of 13 with expansion

## Figure S23-2-2. ^13^C NMR spectrum of 13 with expansion

## Figure S23-3. COSY spectrum of 13

## Figure S23-4. Multiplicity-edited HSQC spectrum of 13

## Figure S23-5. HMBC spectrum of 13

## Figure S23-5-1. HMBC spectrum of 13 with expansion

## Figure S23-5-2. HMBC spectrum of 13 with expansion

## Figure S23-6. UPLC-HR-ESI-MS spectrum of 13

## Figure S24-1. ^1^H NMR spectrum of 14 (DMSO-*d*_6_,700MHz)

## Figure S24-1-1. ^1^H NMR spectrum of 14 with expansion

## Figure S24-2. ^13^C NMR spectrum of 14 (DMSO-*d*_6_, 176MHz)

## Figure S24-2-1. ^13^C NMR spectrum of 14 with expansion

## Figure S24-2-2. ^13^C NMR spectrum of 14 with expansion

## Figure S24-3. COSY spectrum of 14.

## Figure S24-4. Multiplicity-edited HSQC spectrum of 14

## Figure S24-5. HMBC spectrum of 14

## Figure S24-5-1. HMBC spectrum of 14 with expansion

## Figure S24-5-2. HMBC spectrum of 14 with expansion

## Figure S24-6. UPLC-HR-ESI-MS spectrum of 14

## Figure S25. Structures of cryopeptins 1-5

## Figure S26. Key COSY and HMBC correlations of cryopeptins 1-5

## Figure S27. Structures of cryopeptins 6-10

## Figure S28. Key COSY and HMBC correlations of cryopeptins 6-10

## Figure S29. Structures of cryopeptins 11-14

## Figure S30. Key COSY and HMBC correlations of cryopeptins 11-14

## Figure S31. UPLC-HR-ESI-MS analysis of D/L-FDVA-Leu

## Figure S32. UPLC-HR-ESI-MS analysis of D/L-FDVA-Phe

## Figure S33. UPLC-HR-ESI-MS analysis of D/L-FDVA-Arg

## Figure S34. Structures of R- and S-NAc-valines (3.6, 3.7)

## Figure S35-1-1. ^1^H NMR spectra of 3.10 in CDCl_3_.

## Figure S35-1-2. ^13^C NMR spectra of 3.10 in CDCl_3_.

## Figure S35-2-1. ^1^H NMR spectra of 3.11 in CDCl_3_.

## Figure S35-2-2. ^13^C NMR spectra of 3.11 in CDCl_3_.

## Figure S35-3-1. ^1^H NMR spectra of 3.6 in DMSO-*d*_6_.

## Figure S35-3-2. ^13^C NMR spectra of 3.6 in DMSO-*d*_6_.

## Figure S35-4-1. ^1^H NMR spectra of 3.7 in DMSO-*d*_6_.

## Figure S35-4-1. ^13^C NMR spectra of 3.7 in DMSO-*d*_6_.

## Figure S36-1-1. ^1^H NMR spectra of 4.4 in MeOD.

## Figure S36-1-2. ^13^C NMR spectra of 4.4 in MeOD.

## Figure S36-2-1. HMBC spectra of 4.4 in MeOD.

## Figure S36-2-2. HMBC spectra of 4.4 in MeOD.

## Figure S37. MS/MS fragmentation of 4.4 and fragmentation pattern.

## Figure S38-1. ^1^H NMR spectrum of natural isolated compound 4 in MeOD (600 MHz, 150 MHz).

## Figure S38-1-1. ^1^H NMR spectrum (expansion) of natural isolated compound 4 in MeOD (600 MHz, 150 MHz).

## Figure S38-2. ^13^C NMR spectrum of natural isolated compound 4 in MeOD (600 MHz, 150 MHz).

## Figure S38-3. COSY spectrum of natural isolated compound 4 in MeOD

## Figure S38-4. Multiplicity-edited HSQC spectrum of natural isolated compound 4 in MeOD

## Figure S38-5. HMBC spectrum of natural isolated compound 4 in MeOD

## Figure S38-5-1. HMBC spectrum (expansion) of natural isolated compound 4 in MeOD

## Figure S38-5-2. HMBC spectrum (expansion) of natural isolated compound 4 in MeOD
